# Enhanced Energetic State and Protection from Oxidative Stress in Human Myoblasts Overexpressing BMI1

**DOI:** 10.1016/j.stemcr.2017.06.009

**Published:** 2017-07-20

**Authors:** Silvia Dibenedetto, Maria Niklison-Chirou, Claudia P. Cabrera, Matthew Ellis, Lesley G. Robson, Paul Knopp, Francesco Saverio Tedesco, Martina Ragazzi, Valentina Di Foggia, Michael R. Barnes, Aleksandar Radunovic, Silvia Marino

**Affiliations:** 1 Blizard Institute, Barts and The London School of Medicine and Dentistry, Queen Mary University of London, 4 Newark Street, London E1 2AT, UK; 2 Centre for Translational Bioinformatics, William Harvey Research Institute, Barts and The London School of Medicine and Dentistry, Queen Mary University of London, London EC1M 6BQ, UK; 3 Division of Neuropathology, the National Hospital for Neurology and Neurosurgery, Queen Square, London WC1N 3BG, UK; 4 Department of Cell and Developmental Biology, University College London, 21 University Street, London WC1X 0JS, UK; 5 Neuroscience and Trauma Centre, Barts Health NHS Trust, Whitechapel, London E1 1BB, UK

**Keywords:** Polycomb gene, satellite cells, muscle regeneration, myopathy, DMD, myoblasts, oxidative phosphorylation

## Abstract

The Polycomb group gene *BMI1* is essential for efficient muscle regeneration in a mouse model of Duchenne muscular dystrophy, and its enhanced expression in adult skeletal muscle satellite cells ameliorates the muscle strength in this model. Here, we show that the impact of mild BMI1 overexpression observed in mouse models is translatable to human cells. In human myoblasts, BMI1 overexpression increases mitochondrial activity, leading to an enhanced energetic state with increased ATP production and concomitant protection against DNA damage both *in vitro* and upon xenografting in a severe dystrophic mouse model. These preclinical data in mouse models and human cells provide a strong rationale for the development of pharmacological approaches to target BMI1-mediated mitochondrial regulation and protection from DNA damage to sustain the regenerative potential of the skeletal muscle in conditions of chronic muscle wasting.

## Highlights

• BMI1 overexpression enhances energetic state in human dystrophic myoblasts
• It protects the cells from DNA damage both *in vitro* and *in vivo* in a dystrophic model
• BMI1 expression is reduced in chronic muscle wasting independently of its etiology

## Teaser

In this article, Marino and colleagues show that BMI1 overexpression in human myoblasts enhanced their energetic state while conferring protection from DNA damage both *in vitro* and *in vivo* upon xenografting in a dystrophic mouse model. These preclinical data provide a strong rationale for the development of pharmacological approaches to target BMI1-mediated mitochondrial regulation and protection from DNA damage to sustain the regenerative potential of the skeletal muscle.

## Introduction

Myopathies are a heterogeneous group of conditions with diverse etiologies, which affect the muscle without involving the nervous system or the neuromuscular junction. The muscular dystrophies are the most common of such disorders. However, the range of myopathies is broad, they can be not only genetic but also acquired, they are prevalent in all ethnicities, and they range widely in severity. Most are progressive in nature, often leading to muscle weakness and disability; for most there are no effective treatments or cures (reviewed in Marino and Di Foggia, 2016).

Skeletal muscle is able to regenerate throughout the life of individuals mainly due to the function of satellite cells (the main skeletal muscle stem cells [Brack and Rando, 2012, Wang and Rudnicki, 2012, Yin et al., 2013]). Satellite cells are normally quiescent and located beneath the basal lamina of the muscle fibers. After muscle injury or in diseases the satellite cells become activated and re-enter the cell cycle to provide repair cells as well as repopulating the stem cell pool (Collins et al., 2005, Hill et al., 2003, Sambasivan et al., 2011). In primary chronic myopathies, such as the muscular dystrophies, there is widespread degeneration of the skeletal muscle, and changes in the ability of the satellite cells to repair the muscle contribute to the progressive worsening of the pathology (Dumont et al., 2015; reviewed in Chang et al., 2016).

Muscular dystrophies are predominantly caused by defects in proteins that are part of the dystrophin-glycoprotein complex which form the connections between muscle cells and their surrounding cellular structure (Rando, 2001). They are currently untreatable, with many forms leading to severe disabilities and the most severe forms leading to death in early adulthood. Novel therapies are currently being developed and comprise antisense oligonucleotide-mediated exon skipping or gene replacements to restore protein expression and stem cell transplantation or a combination of both approaches (Benedetti et al., 2013). Of particular interest, the recent application of the induced pluripotent stem cell technology to derive satellite cells and myoblasts from induced pluripotent/embryonic stem cells obtained from human fibroblasts as a tool to overcome limitations in the expansion of a sufficient number of myoblasts for transplantation (Chal et al., 2015, Loperfido et al., 2015, Maffioletti et al., 2015).

Polycomb group (PcG) proteins are essential regulators of stem cell function during normal development and in adult organs (Marino and Di Foggia, 2016). They form multiprotein chromatin-associated complexes that play an essential role in the genome-wide epigenetic-mediated remodeling of gene expression during the myogenic differentiation of satellite cells, mainly through post-translational modifications of histones (Asp et al., 2011). BMI1 and EZH2 play an essential role in adult satellite cell homeostasis and proliferation in response to muscle injury, an effect mediated at least in part by repression of the ink4a locus (Juan et al., 2011, Robson et al., 2011).

An emerging role for PcG proteins is their involvement in DNA repair (Facchino et al., 2010, Ginjala et al., 2011, Ismail et al., 2010, Liu et al., 2009, Pan et al., 2011) and in maintaining redox balance (Chen et al., 2015, Jin et al., 2014). BMI1^−/−^-derived cells show significant mitochondrial dysfunction accompanied by a sustained increase in the production of reactive oxygen species (ROS) that are sufficient to engage the DNA repair pathway (Liu et al., 2009), which is in turn impaired, thus leading to magnified cellular damage. The balance between intracellular ROS and antioxidant molecules is vital in determining the rate of oxidative damage accumulation and the impaired function of satellite cells in aging and in myopathies, where decreased antioxidative capacity has been documented (Fulle et al., 2005, Tidball and Wehling-Henricks, 2007, Whitehead et al., 2006).

In Duchenne muscular dystrophy (DMD) the overall number of satellite cells is not affected, but their proliferative capacity rapidly declines during progression of the disease (Blau et al., 1983, Endesfelder et al., 2000, Gnocchi et al., 2008, Mouly et al., 2005), an effect due at least in part to their increased sensitivity to oxidative stress injury leading to reduced and defective regeneration of the muscle (Blau et al., 1983, Blau et al., 1985, Disatnik et al., 2000). Moreover, enzymatic adaptations to exercise-induced production of ROS and free radical damage are significantly decreased in dystrophic compared with normal muscles (Faist et al., 1998, Faist et al., 2001). Overall, an impaired protection against ROS in dystrophic muscle appears to contribute to disease progression, as also indicated by the beneficial effect of antioxidants in ameliorating the skeletal muscle pathophysiology in DMD patients (Whitehead et al., 2008).

We have recently shown that BMI1 is essential for efficient muscle regeneration, especially after repeated muscle injury and in a mouse model of DMD, the *Mdx* mouse (Robson et al., 2011). Conditional overexpression of BMI1 in the satellite cells of the adult skeletal muscle enhances their regenerative capacity in this model, leading to improved muscle strength. BMI1 exerts this effect, at least in part, by protecting the satellite cells from oxidative stress-induced DNA and cellular damage via upregulation of metallothionein 1 (Di Foggia et al., 2014).

Here we show that the impact of mild BMI1 overexpression observed in mouse models is translatable to human cells. In human myoblasts, BMI1 overexpression increases mitochondrial activity, leading to an enhanced energetic state with increased ATP production. Concomitantly it protects the cells from DNA damage both *in vitro* and *in vivo* upon xenografting in a severe dystrophic mouse model.

## Results

### BMI1 Expression Is Reduced in Quiescent and Committed DMD Satellite Cells

**Figure 1:**
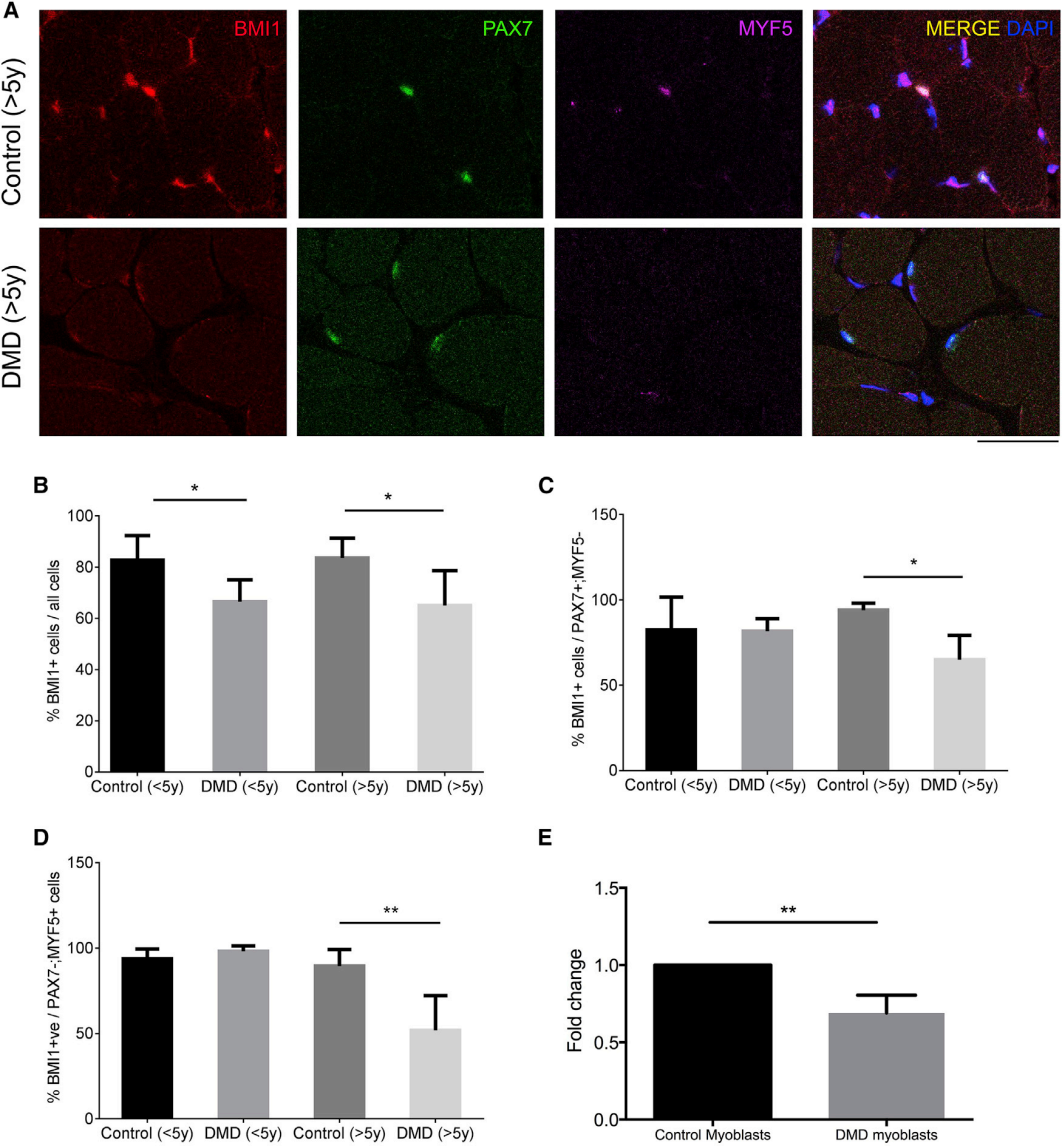
Depletion of BMI1^+^ Cells in Quiescent and Committed Satellite Cells in DMD Patients (A) Triple immunostaining for BMI1, PAX7, and MYF5 on frozen muscle transverse sections from DMD patients (n = 4 patients <5 years old; n = 4 patients >5 years old) and age-matched controls (n = 4 patients <5 years old; n = 4 patients >5 years old). Representative images of the staining on >5-year-old DMD and control muscles are shown. Scale bar, 125 μm. (B) Quantification of BMI1^+^ cells over the total number of cells (mean ± SD; ^∗^p < 0.05). (C and D) Quantification of BMI1^+^ cells among quiescent (PAX7^+^; MYF5^−^) (C) and committed (PAX7^−^; MYF5^+^) (D) satellite cells (mean ± SD; ^∗^p < 0.05, ^∗∗^p < 0.01). Quantification was carried out on at least 5 high-power fields (40×) for each case. (E) *BMI1* expression at the RNA level in DMD human primary myoblasts compared with normal human myoblasts (mean ± SD of three independent preparations; ^∗∗^p < 0.01).

We have previously shown that the expression of BMI1 is significantly reduced in quiescent satellite cells in muscle biopsies from DMD patients, a finding mirrored by the downregulation of BMI1 expression in the satellite cells of the *Mdx* mouse (Di Foggia et al., 2014). Here, we set out to further dissect the expression of BMI1 in muscle biopsies of DMD patients. To reflect the progression of the disease throughout aging, we divided patients into two groups: younger (n = 4) and older (n = 4) than 5 years old. Age-matched patients with a muscle biopsy without histological abnormalities were included in the study as controls (<5 years, n = 4; >5years, n = 4). Immunostaining for BMI1 confirmed the previously reported overall reduction of positive cells in both young and older DMD patients compared with the control group in this extended number of patients (Figures 1A and 1B). Co-immunostaining for BMI1, PAX7, and MYF5 showed that the decrease of BMI1^+^cells affected both PAX7^+^; MYF5^−^ quiescent and PAX7^−^; MYF5^+^ committed myoblasts in older DMD patients (Figures 1A, 1C, and 1D), while no difference was seen in younger patients (Figures 1A, 1C, and 1D). Immunostaining for EZH1 revealed an overall reduction of the EZH1^+^ cells (Figures S1A and S1B) and also of the EZH1 and BMI1 double-positive cells in both groups of DMD patients compared with their age-matched controls (Figures S1A and S1C). In this case, however, the difference was due to a reduction in the PAX7^−^ population rather than in the PAX7^+^ (Figures S1D and S1E).

In summary, a time-dependent depletion of quiescent and activated satellite cells expressing BMI1 but not EZH1 is noted in DMD, while an overall reduction in the myonuclei expressing BMI1 and EZH1 is detected, raising the possibility that fluctuation in the expression of BMI1 may be more relevant for satellite cells' biology.

### BMI1 Overexpression Increases Differentiation but Not Proliferation in DMD Myoblasts

**Figure 2:**
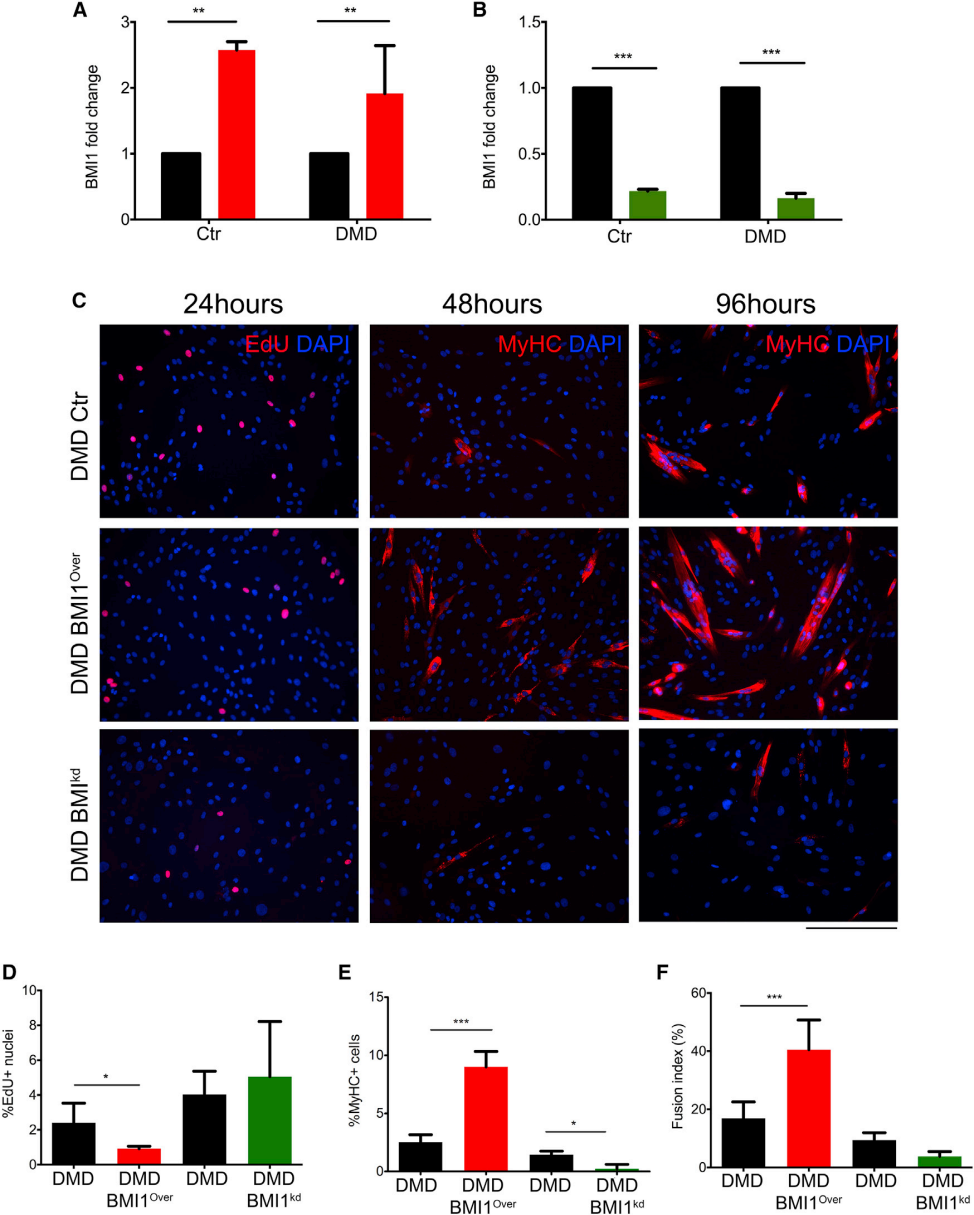
Increased Differentiation in DMD Human Myoblasts Overexpressing BMI1 (A and B) DMD and control human myoblasts are infected with a GFP (black bar) or BMI1 (red bar) encoding lentiviral particles (A) and with an SCR (black bar) or BMI1^−^ shRNA (green bar) lentiviral particles (B). Level of expression of *BMI1* upon BMI1 overexpression (A) or BMI1 knockdown (B) was assessed by qRT-PCR (mean ± SD of three independent preparations; ^∗∗^p < 0.01, ^∗∗∗^p < 0.001). (C–F) Representative images (C) of EdU staining and MyHC staining on DMD human myoblasts at 24, 48, and 96 hr after induction of differentiation. Quantification of the percentage of positive cells for EdU and MyHC over total number of cells is shown in (D) and (E). Differentiation rate at 96 hr is expressed as fusion index (F) (mean ± SD of three independent experiments; ^∗^p < 0.05, ^∗∗∗^p < 0.001). Quantification was carried out on at least 5 fields (20×) for each case. Scale bar, 250 μm.

Next we evaluated the impact of BMI1 modulation on human satellite cell function. Short-term cultures of human satellite cell-derived myoblasts isolated from DMD patients (n = 3) and control donors (n = 3) (Mamchaoui et al., 2011) were used for these studies. Reduced expression of *BMI1* was confirmed in DMD myoblasts compared with age-matched cultures at the RNA level (Figure 1E), in keeping with our observation in the muscle tissue (Di Foggia et al., 2014). Overexpression of BMI1 was achieved by lentiviral-mediated cell transduction and confirmed at the RNA and protein levels (Figure 2A and data not shown). Short hairpin RNA (shRNA)-mediated knockdown of BMI1 was also performed in these cultures and its efficacy confirmed as above (Figure 2B and data not shown).

In normal myoblasts, BMI1 overexpression induced increased proliferation, as assessed by 5-ethynyl-2′-deoxyuridine (EdU) incorporation and detection 24 hr after induction of differentiation (Figures S2A and S2B).

To assess the impact of BMI1 overexpression on muscle differentiation, we evaluated the percentage of MyHC^+^ cells and the percentage of multinucleated cells (fusion index) after 48 hr or 4 days in differentiation medium. We show that BMI1 overexpression enhances the differentiation process in normal human myoblasts, as demonstrated by the increased percentage of MyHC^+^ cells (Figures S2A and S2C) and the increased fusion index leading to the formation of larger myotubes (Figures S2D).

BMI1 knockdown induced a drastic reduction of the proliferation rate (Figures S2A and S2B) and of the differentiation potential (Figures S2A–S2D), as expected if this was a BMI1-dependent effect.

Increased proliferation was not observed in DMD myoblasts upon BMI1 overexpression, with a reduced percentage of EdU^+^ cells seen instead (Figures 2C and 2D). However, enhanced differentiation was noted in these cultures, as assessed by the increased percentage of MyHC^+^ cells (Figures 2C and 2E) and increased fusion index (Figures 2D and 2F), an effect which was more pronounced than in normal myoblasts (9% MyHC^+^ cells versus 2.5%, respectively, p < 0.001). BMI1 knockdown had no effect on the proliferation of the cells while reduced differentiation was observed (Figures 2C–2F).

Taken together, these data show that overexpression of BMI1 ameliorates the differentiation potential of DMD myoblasts in culture.

### Deregulation of Cellular Redox and Mitochondrial Target Genes in DMD BMI1^Over^ Myoblasts and Control

To identify the downstream molecular effectors of BMI1 overexpression in myoblasts, we carried out a whole-genome transcriptome analysis on cultures isolated from three DMD patients and three age-matched controls. In particular, we set out to assess the transcriptome-wide impact of overexpression of BMI1 in DMD myoblasts compared with the same primary cell lines treated with GFP-encoding plasmid (pLoxGFP) as control (DMD versus DMD BMI1^Over^). The impact of overexpressing BMI1 in control cultures (Ctr versus Ctr BMI1^Over^) was also assessed, and a cross-comparison of the datasets was carried out to identify shared and condition-specific target genes.

**Figure 3:**
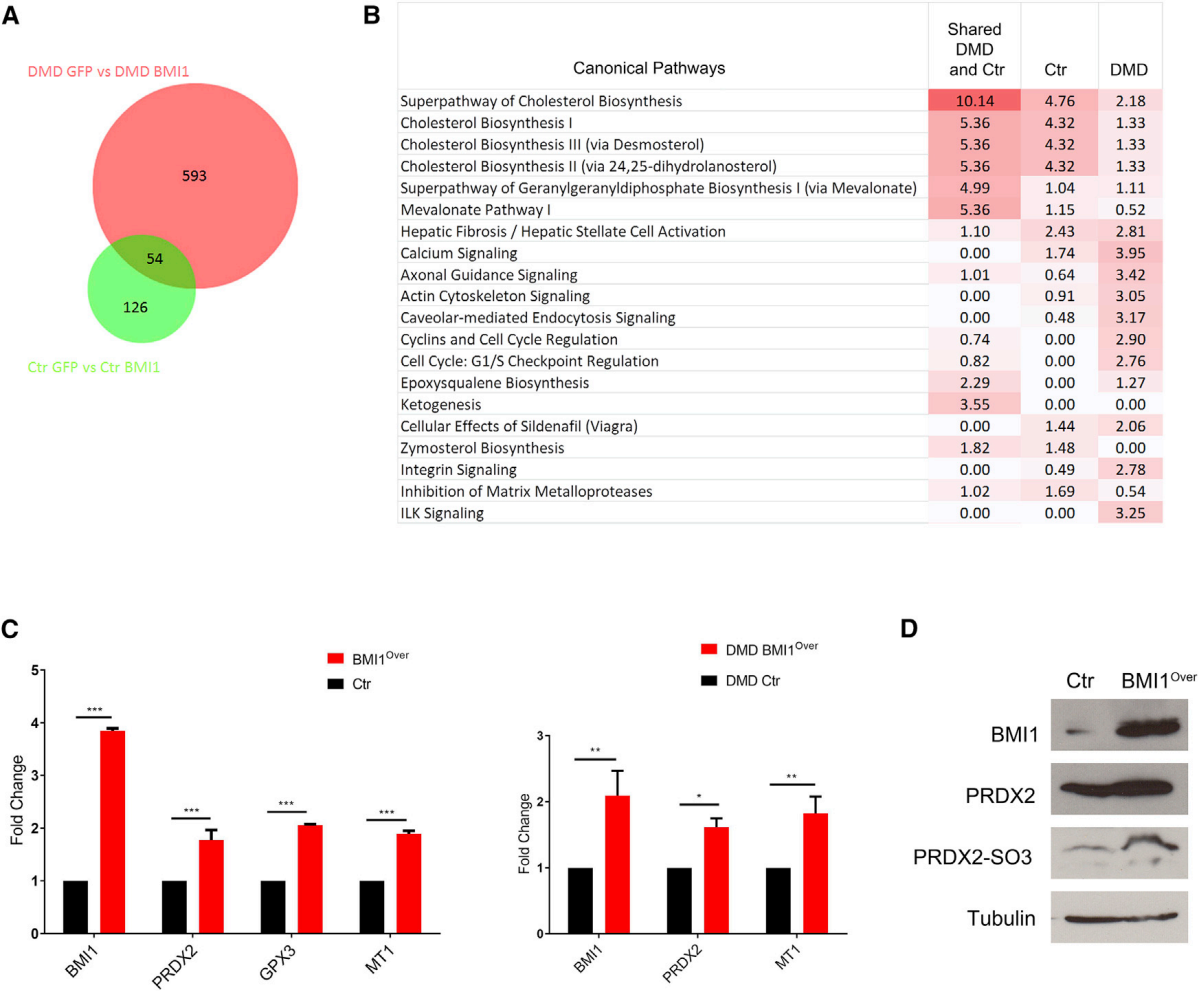
Deregulation of Cellular Redox and Mitochondrial Target Genes in DMD BMI1^Over^ Myoblasts and Controls (A) Venn diagram to identify common deregulated (DE) genes in control and DMD myoblasts overexpressing BMI1. (B) Canonical pathway enrichment (reported as –log_10_ p value) for the 54 shared genes as compared with DMD and control-only DE genes. (C) Validation of selected target genes in independent biological replicas (mean ± SD of three independent preparations; ^∗^p < 0.05 ^∗∗^p < 0.01; ^∗∗∗^p < 0.001). (D) Level of PRDX2 and its oxidized form, PRDX2-SO3, as assessed by western blot analysis.

The exploratory analysis showed 647 genes differentially expressed in DMD versus DMD BMI1^Over^ (344 upregulated in DMD BMI1^Over^ and 303 downregulated) and 180 in Ctr versus Ctr BMI1^Over^ (71 upregulated in Ctr BMI1^Over^ and 109 downregulated) when a statistical significance threshold p value of ≤0.05 was applied. Comparative analysis of these two datasets revealed 54 shared target genes with 593 and 126 specific target genes for the DMD and control comparison, respectively (Figure 3A). Pathway analysis carried out on the Ingenuity platform highlighted deregulation of functions and pathways essential for cell growth and differentiation in the analyzed gene sets (Figure 3B). All stages of cholesterol biosynthesis, including the mevalonate and ketogenesis pathways, were significantly enriched in the shared gene set while calcium signaling, cyclins, and cell-cycle regulation were deregulated in the DMD comparison (Figure 3B).

Upregulation of *MT-1* was noted among the upregulated shared genes (Figure 3C), a finding consistent with previous findings in a mouse model (Di Foggia et al., 2014).

Upregulation of genes involved in maintenance of the cellular redox balance in cells, *Peroxiredoxin2* (*PRDX2*) and *Glutathione peroxidase 3* (*GPX3*), were validated in independent myoblast preparations (Figure 3C). A network connectivity analysis of BMI1 and PDRX2 revealed a predicted molecular relationship in DMD overexpressing BMI1 (Figures S3A–S3C). Importantly, overexpression of BMI1 not only increased the expression levels of PRDX2, but also of the oxidized PRDX2 form, PRDX2-SO3 (Figure 3D), indicating that BMI1^Over^ myoblasts display an enhanced capacity to balance the cellular redox state and protect the myoblasts from oxidative stress.

Mitochondrial dysfunction with its links to deregulation of cholesterol biosynthesis and impairment of energy homeostasis are known to occur in DMD (Onopiuk et al., 2009, Percival et al., 2013, Rybalka et al., 2014, Yoon et al., 2016); hence, our findings raise the possibility that BMI1 overexpression ameliorates the differentiation potential of DMD myoblasts by impacting these cellular functions.

### BMI1 Overexpression Impacts on the Metabolic State of DMD Myoblast Cultures

Because myoblasts can use both oxidative phosphorylation (OXPHOS) and aerobic glycolysis as a source of energy (Paasuke et al., 2016), we measured the oxygen consumption rate (OCR), an indicator of OXPHOS, and the extracellular acidification rate (ECAR), an indicator of aerobic glycolysis, to assess the cellular bioenergetics profile of myoblasts upon BMI1 overexpression. Compounds modulating mitochondrial function (oligomycin, carbonyl cyanide-4-(trifluoromethoxy)phenylhydrazone [FCCP], and rotenone/antimycinA) were added sequentially to the cells and the effect on OCR was measured after each compound addition with a Seahorse Biosciences XF24 analyzer. Firstly, we observed a loss of spare respiratory capacity (SRC) in DMD myoblasts compared with control cells (Figure S4A), in keeping with the known mitochondrial dysfunction in DMD leading to impairment of OXPHOS with concomitant decrease of the SRC (Kuznetsov et al., 1998).

**Figure 4:**
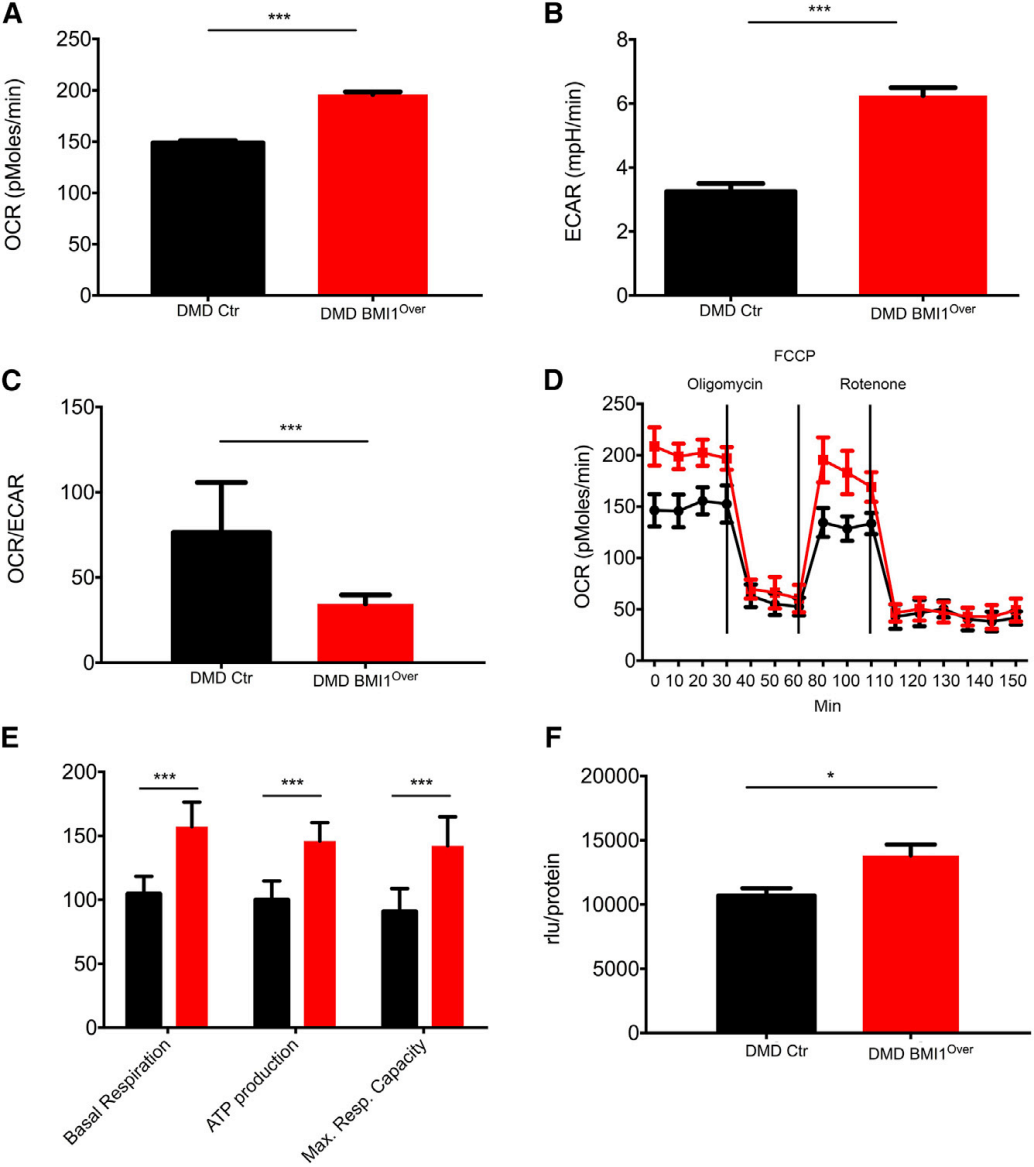
BMI1 Overexpression Enhances Mitochondrial Respiration and ATP Production (A) Basal oxygen consumption rate (OCR, indicative of mitochondrial OXPHOS) was measured in DMD myoblasts overexpressing BMI1 (red) and compared with DMD myoblasts (black) with an XF24 Extracellular Analyzer (^∗∗∗^p < 0.001). (B and C) Measurement of basal extracellular acidification rate (ECAR, representing glycolysis) measured in DMD control myoblasts (black) and overexpressing BMI1 (red) (B), and OCR/ECAR ratios of DMD myoblasts control (black) and overexpressing BMI1 (red) (C) (^∗∗∗^p < 0.001). (D) DMD control myoblasts (black) and overexpressing BMI1 (red) were seeded in a Seahorse XF-24 analyzer and real-time OCR was determined during sequential treatments with oligomycin (ATP-synthase inhibitor), FCCP (mitochondria uncoupler), and rotenone (ETC inhibitors). Data are from three independent experiments in which each data point represents the mean of 12 replicates for each condition ± SD. (E) Basal respiration rate, ATP production, and maximal respiration rate in DMD control myoblasts and overexpressing BMI1 (calculated from data shown in D; ^∗∗∗^p < 0.001). (F) ATP content measured with a luciferase assay and normalized against protein concentration in DMD control and DMD BMI1^Over^ myoblasts (rlu/prot) (mean ± SD of three independent experiments; ^∗^p < 0.05).

Importantly, BMI1 overexpression in DMD myoblasts led to a 33% increase in OXPHOS compared with DMD myoblasts with basal levels of BMI1 (p = 0.0001) (Figure 4A). An increase in aerobic glycolysis was also observed in DMD BMI1^Over^ myoblasts as assessed by ECAR and OCR/ECAR ratio evaluation (Figures 4B and 4C). As OXPHOS and aerobic glycolysis represent the two main energy sources for cells, the prediction is that BMI1 overexpression has a significant impact on the energetic state of the cells (ATP levels). Bioenergetic profiling of DMD myoblasts overexpressing BMI1 showed increased oxygen consumption in basal condition with a concomitant increase in the calculated ATP levels, and a greater maximal respiration capacity (Figures 4D and 4E). Increased ATP production in DMD BMI1^Over^ myoblasts as assessed by an ATP luciferase assay (Figure 4F) lends support to this conclusion. Importantly, increased ATP production and increased OCR levels were validated in non-immortalized DMD myoblasts upon overexpression of BMI1 (Figures S4C–S4F) with the opposite effect detected upon silencing of BMI1 (Figures S4C, S4D, S4E, and S4G).

To assess whether the effect on respiratory capacity was dependent on an increase in the efficiency of the mitochondria or on an increase in the number of organelles available in the cells, we measured the mtDNA copy number. We did not see an increase in mitochondria number (Figure S4B), in keeping with the observed effect being exerted by BMI1 on mitochondrial activity.

### BMI1 Overexpression Protects DMD Myoblasts from Oxidative Damage

**Figure 5:**
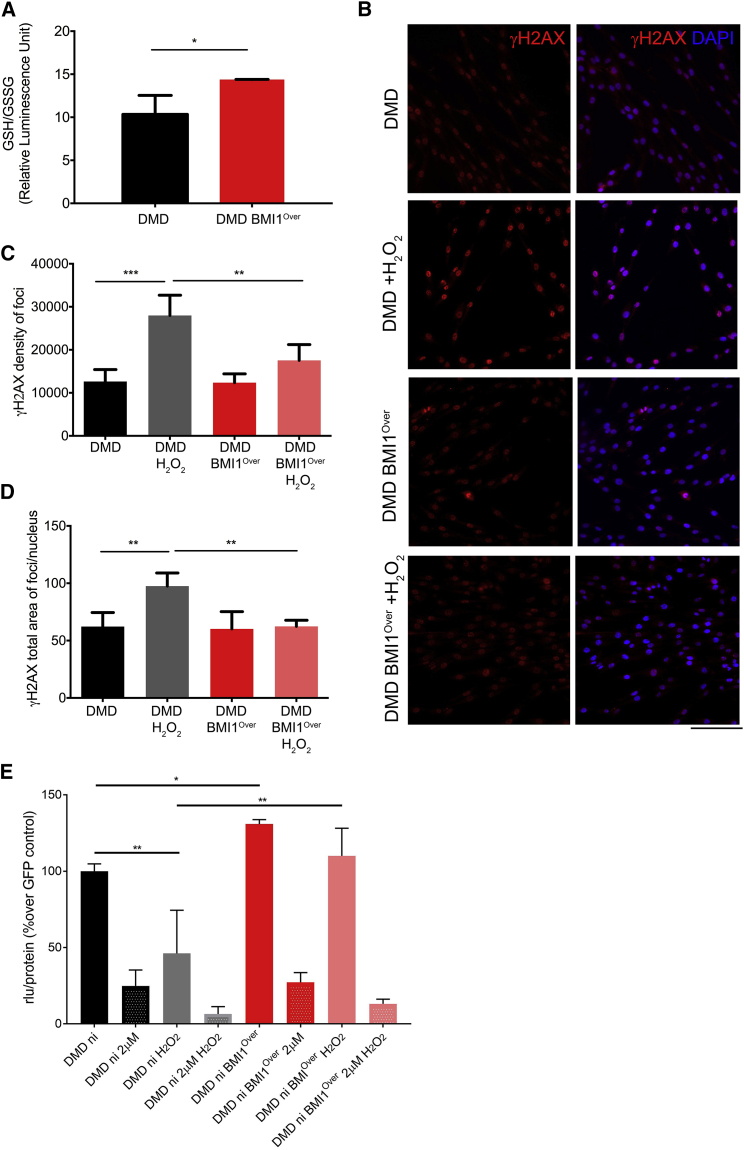
Increase of Reduced Glutathione Form and Protection against DNA Damage upon BMI1 Overexpression (A) GSH/GSSG ratio expressed as relative luminescence unit (rlu) in DMD myoblasts overexpressing BMI1 compared with DMD control (mean ± SD of three independent experiments; ^∗^p < 0.05). (B–D) Representative images (B) of γH2AX staining of DMD myoblasts overexpressing BMI1 and DMD controls either untreated or treated with 50 mM H_2_O_2_. Quantification of mean intensity (C) and mean area (D) of foci per nucleus (mean ± SD of three independent experiments; ^∗∗^p < 0.01, ^∗∗∗^p < 0.001). Quantification of the number of positive cells was carried out on at least 9 fields (20×) for each case using the InCell Developer Toolbox software (GE Healthcare). Scale bar, 250 μm. (E) Effect of conoidin A (2 μM) treatment on ATP level, in normal condition or upon treatment with 50 μM H_2_O_2_ in DMD BMI1^Over^ myoblasts compared with controls (mean ± SD of three independent experiments; ^∗^p < 0.05, ^∗∗^p < 0.01).

Glutathione (GSH) is the main antioxidant of aerobic cells and prevents damage to important cellular components caused by ROS, such as free radicals and peroxides (Mari et al., 2009). Because we observed upregulation of genes involved in glutathione regulation (*PRDX2* and *GPX3*) in BMI1^Over^ myoblasts, we set out to further analyze the GSH/GSSG ratio in DMD cultures, where a reduction in the GSH/GSSG ratio is considered indicative of oxidative stress (Owen and Butterfield, 2010). We observed an increased GSH/GSSG ratio in DMD BMI1^Over^ myoblasts compared with control DMD cells (Figure 5A), indicating a reduced oxidative stress in DMD BMI1^Over^ myoblasts.

Because PRDX2 is known to protect DNA from hydrogen peroxides and thermal stress damage (Lee et al., 2015), we set out to assess the degree of oxidative DNA damage in DMD myoblasts overexpressing BMI1 to determine whether its increase of expression could facilitate the cellular compliance with the higher energetic state induced by BMI1^Over^ and sustain differentiation through protection from DNA damage.

To assess the response to induction of DNA damage in DMD BMI1^Over^ myoblasts, we performed a 10-min challenge with 50 μM H_2_O_2_ and assessed DNA damage by γH2AX staining, a sensitive marker of double-strand DNA breaks (Mariotti et al., 2013). While control DMD cells showed a significant increase in the intensity and total area of γH2AX foci per nucleus, DMD BMI1^Over^ myoblasts did not show this increase and both the intensity and the total area of γH2AX foci were unchanged compared with the non-treated cells (Figures 5B–5D), in keeping with DMD BMI1^Over^ myoblasts being more resistant to DNA damage.

To validate the dependency on PRDX2 of the impact of BMI1 on oxidative stress protection, we treated DMD BMI1^Over^ and controls with conoidin A, a well-characterized PRDX2 inhibitor. Measurement of the ATP level in DMD BMI1^Over^ myoblasts upon H_2_O_2_ treatment showed that its increase is neutralized upon treatment with conoidin A (Figure 5E). These data are consistent with PRDX2 mediating, at least in part, the effect of BMI1^Over^ on the energetic state of the cells.

### DMD BMI1^Over^ Myoblasts Are Protected from DNA Damage upon Engrafting into a Dystrophic Mouse Model

**Figure 6:**
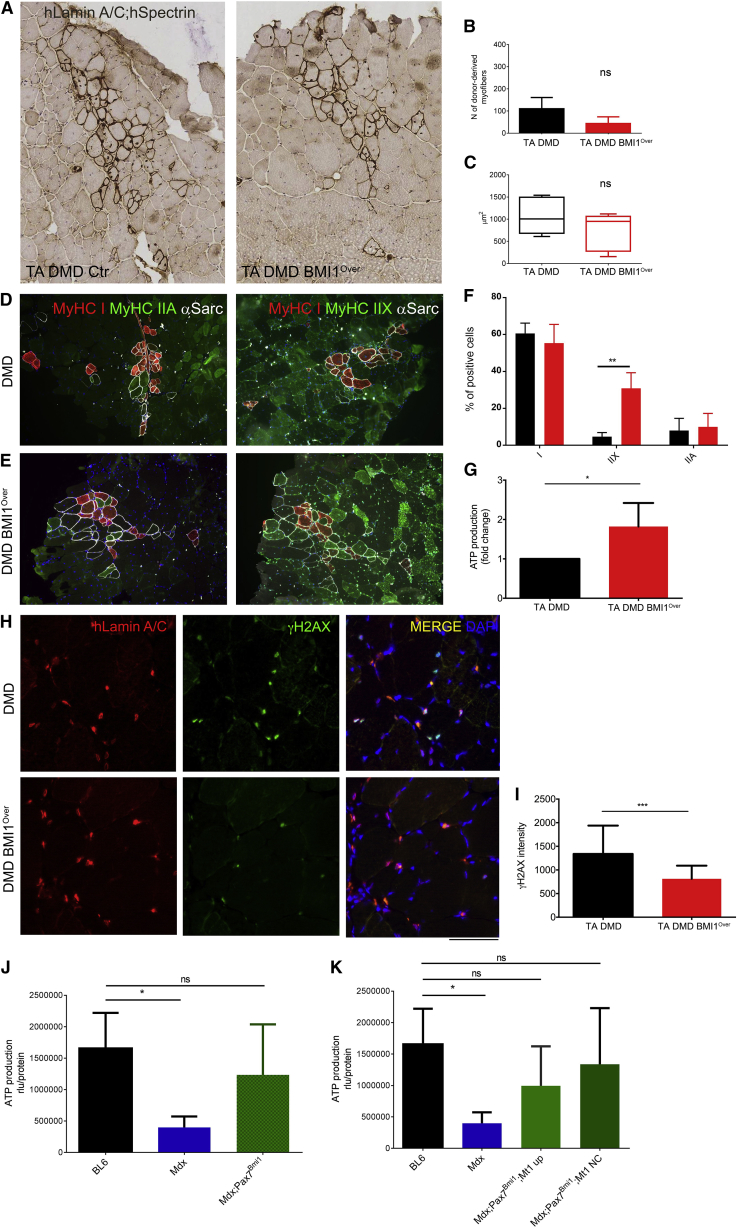
BMI1 Overexpression Increases ATP and Reduces DNA Damage upon Transplantation of DMD BMI1^Over^ Myoblasts in Sgca-null/scid/bg TA Muscle (A–I) Representative images of hLaminA/C and hSpectrin 3,3′-diaminobenzidine staining (A) on frozen transverse sections of Sgca-null/scid/bg TA injected with DMD Ctr or DMD^Over^ human myoblasts and analyzed 25 days after injection. Quantification with Definiens software of the number of integrated fiber (B) and the mean CSA (μm^2^) (C) (mean ± SD, n = 6 animals; ns, not significant). Representative images of MyHC I (red), MyHC IIA or MyHC IIX (green), and α-sarcoglycan (white) staining on frozen transverse sections of Sgca-null/scid/bg TA injected with DMD Ctr (D) or DMD^Over^ (E) human myoblasts. Quantification of positive fibers for MyHC antigens expressed as percentage over total number of α-sarcoglycan-positive fibers (F) (mean ± SD, n = 6 animals; ^∗∗^p < 0.01) and (G) ATP quantification on scraped frozen transverse sections (^∗^p < 0.05). Representative images (H) and quantification (I) of γH2AX and hLamin A/C staining on transverse sections of muscles injected with DMD Ctr or DMD^Over^ human myoblasts (mean ± SD, n = 6 animals; ^∗∗∗^p < 0.001). (J and K) ATP quantification on transverse section of forelimb muscles isolated from BL6 (n = 4), Mdx (n = 6), Mdx; Pax7; Bmi1; Mt up (n = 4), and Mdx; Pax7; Bmi1; Mt NC (n = 7) expressed as relative luminescence unit normalized versus protein concentration (^∗^p < 0.05; ns, not significant). The scale bar represents 500 μm in (A, D, and E) and 125 μm in (H).

To investigate whether modulation of BMI1 expression in human myoblasts could be exploited therapeutically to enhance the regenerative potential of the skeletal muscle in pathological conditions, we performed an engraftment of human myoblasts in a dystrophic mouse host. 10^6^ DMD BMI1^Over^ myoblasts and DMD controls were injected intramuscularly into the tibialis anterior (TA, n = 6.) muscles of α-sarcoglycan null/scid/beige dystrophic mice (Tedesco et al., 2012). The number of donor-derived myofibers and their size were assessed in serial sections double stained for hLaminA/C and hSpectrin 25 days after transplantation. No significant difference was observed in terms of number or cross-sectional area (CSA) of donor-derived myofibers (Figures 6A–6C) upon BMI1 overexpression. Although we could detect rare PAX7^+^ satellite cells of human origin, no increase was detected in muscles that had been transplanted with BMI1-overexpressing cells (data not shown). However, BMI1^Over^ affected the energetic state of the transplanted muscles, as an increase (1.8-fold change) in ATP content was detected in TA muscles injected with BMI1^Over^ cells compared with the controls (Figure 6D). Staining for MyHC I and MyHC IIA/MyHCIIX to identify slow and moderately fast/fast fiber types, respectively, show a higher number of MyHC IIX fast fibers among the fibers originating from the DMD BMI1^Over^ myoblasts engrafted into the dystrophic TA muscles (Figures 6E–6G). Importantly, these fibers use ATP as a major storage fuel for their short-term anaerobic activity; thus, these data are in agreement with the increased ATP production as biochemically assessed (Figures 6D and 6G).

As a further parallel to the *in vitro* results, a reduction of DNA damage was observed in TA muscles engrafted with DMD BMI1^Over^ myoblasts compared with controls in a double staining for γH2AX and hLaminA/C (Figures 6E and 6F).

Because the number of fibers of donor origin was relatively small and the repair was segmental, an assessment of muscle strength was not possible in the xenografted mice.

**Figure 7:**
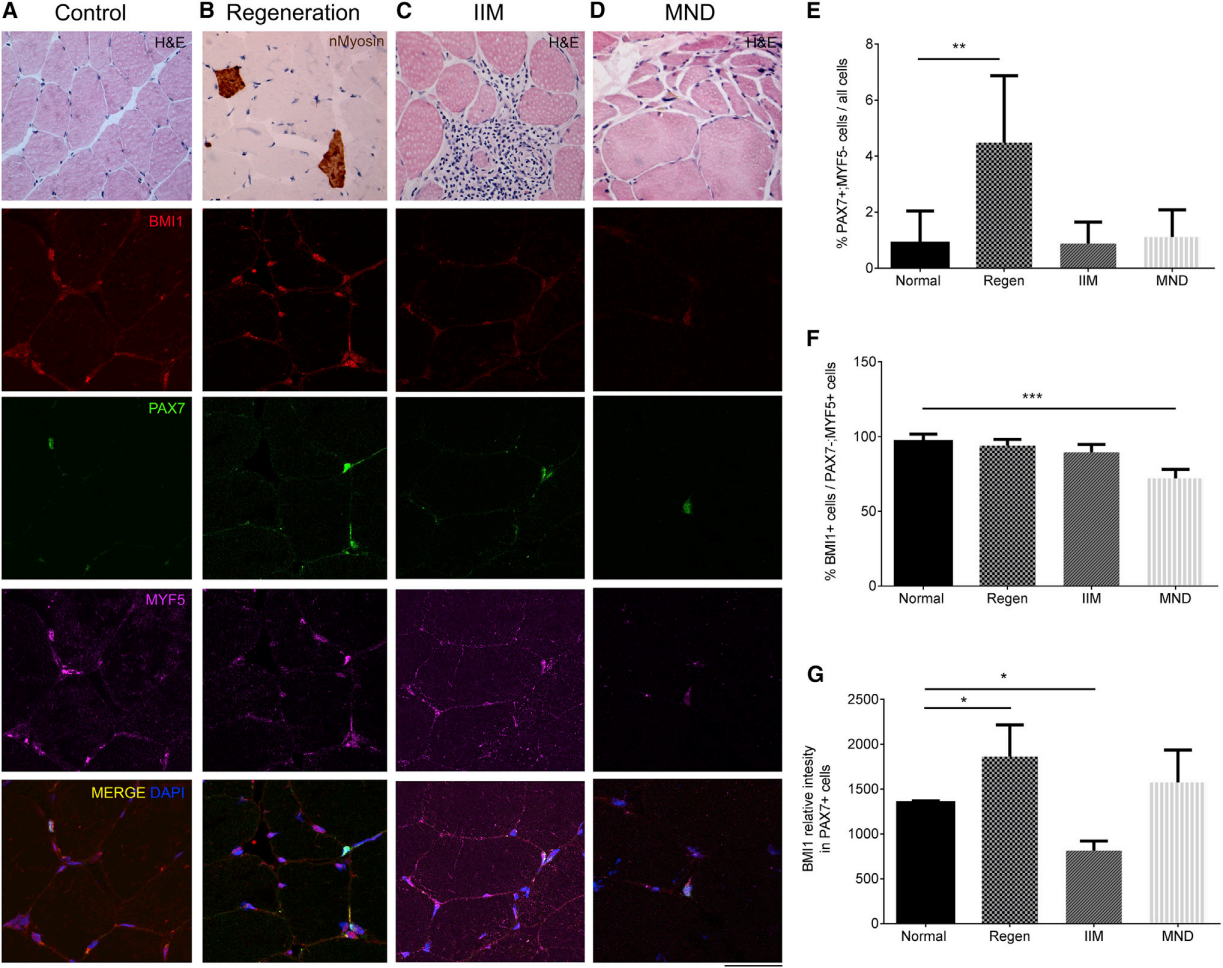
Characterization of BMI1 Expression in Human Chronic Neuromuscular Disorders (A–D) Histological features (H&E) and representative images of a triple immunostaining for BMI1, PAX7, and MYF5 on frozen muscle transverse sections from morphologically normal muscle (n = 6) (A), normal biopsies with evidence of regeneration (n = 5) (B), idiopathic inflammatory myopathy (IIM) (n = 5) (C), and motor neuron disease (MND) (n = 4) (D). Scale bar, 125 μm. (E) Quantification of the number of quiescent satellite cells (PAX7^+^; MYF5^−^) over the total number of nuclei (mean ± SD; ^∗∗^p < 0.01). (F) Quantification of BMI1^+^ cells among committed (PAX7^−^; MYF5^+^) satellite cells (mean ± SD; ^∗∗∗^p < 0.001). (G) BMI1 intensity level in PAX7^+^ cells (mean ± SD; ^∗^p < 0.05). Quantification of the number of positive cells was carried out on at least 5 high-power fields (40×) for each case.

We have previously shown (Di Foggia et al., 2014) that Bmi1 overexpression in satellite cells leads to improved muscle strength in *Mdx* mice, resulting in a better performance on a treadmill compared with controls. Enhanced protection from oxidative stress was essential for the improved functional outcome and was observed only when Bmi1 overexpression triggered MT-1 upregulation. To validate the hypothesis that the increased ATP levels are an essential component of BMI1-mediated enhanced muscle strength, we measured ATP in *Mdx*, *Mdx*;*Pax7*^*Bmi1*^, and control littermates. We show reduced ATP in *Mdx* muscles, although its levels are similar to the normal control in *Mdx* muscles overexpressing Bmi1 in Pax7^+^ satellite cells (Figure 7A), in keeping with the interpretation that a higher energetic state is responsible for the better functional performance observed. Interestingly however, no difference in the ATP levels was observed in the group overexpressing MT1 compared with the group not overexpressing MT1 (Figure 7B), raising the possibility that the increased energetic state translates to a better functional performance only when enhanced protection from oxidative stress also occurs (Di Foggia et al., 2014). Our data show increased ATP levels and protection from oxidative stress in mice xenografted with BMI1^Over^ myoblasts, raising the possibility that BMI1 overexpression may confer enhanced muscle strength also in this model.

### PcG Protein Expression Is Reduced in Quiescent and Committed Satellite Cells in Chronic Neuromuscular Diseases Independently of Their Etiology

To extend our findings to other human muscular conditions, we have characterized the expression of PcG proteins in a wide spectrum of neuromuscular disorders, including inflammatory myopathies and motor neuron disease (MND). To better understand the role of PcG in the regenerative process in human muscles, we also included biopsies from morphologically normal muscles with evidence of regeneration in the study. Cases that did not show histological abnormalities at the time of biopsy were considered normal comparators.

Immunofluorescence for BMI1, including co-localization with markers of quiescent and activated satellite cells, was performed on six cases with normal morphology, five biopsies with evidence of regeneration and a normal morphology, five cases of idiopathic inflammatory myopathy (IIM), and four cases of MND (Figure 7). A significant increase in the percentage of PAX7^+^; MYF5^−^ quiescent satellite cells was noted in cases with regeneration, with no changes seen in the other conditions (Figures 7A–7D and 7E). A significant depletion of Bmi1^+^ committed satellite cells (PAX7^−^; MYF5^+^) was seen in MND cases while no changes were observed in all other conditions (Figures 7A–7D and 7F). No difference was noted in the percentage of BMI1^+^ cells in PAX7^+^; MYF5^−^ and double-positive cells (data not shown).

Interestingly, higher BMI1 expression is seen in the PAX7^+^ cell population in regenerating muscles, while lower BMI1 expression is seen in the PAX7^+^ cell population in IIM cases (Figure 7G).

Triple immunolabeling for PAX7, BMI1, and EZH2 (Figure S5) revealed an overall decrease of EZH2^+^ cells in IIM and MND cases (Figure S6A) with a significant decrease of EZH2^+^ cells in PAX7^+^ satellite cell (Figure S6B). A decrease of BMI1^+^ and EZH2^+^ cells was observed in MND cases (Figure S6C).

In line with what was observed for BMI1, higher EZH2 expression was also observed in PAX7^+^ cells (Figure 6F).

Immunostaining for EZH1 was also performed on the same selection of cases, revealing a decrease in EZH1^+^ cells in IIM and MND (Figure S6D).

We show here that the expression of PcG proteins is decreased in both quiescent and committed satellite cells of chronic neuromuscular diseases, independently of their defining etiology.

## Discussion

We demonstrate here that BMI1 overexpression in human myoblasts increases the mitochondrial activity and the aerobic glycolysis reaction leading to an enhanced energetic state with increased ATP production, and ameliorates the myogenic differentiation capacity of the myoblasts. The concomitant protection of the cells from DNA damage both *in vitro* and *in vivo* upon xenografting in a dystrophic mouse model lends support to the notion that a combination of increased energetic state and protection from oxidative stress is essential for a functional impact of BMI1 overexpression on muscle homeostasis in dystrophic mouse models. These data show that our observation in genetically engineered mouse models (Di Foggia et al., 2014) is translatable to human cells.

The role of PcG proteins during muscle regeneration was recently studied in mouse models. BMI1 expression is upregulated in satellite cells during the first phases of muscle regeneration, and BMI1 ablation leads to an increased proportion of committed myogenic precursor cells (Di Foggia et al., 2014). In keeping with a depletion of the satellite cell pool, when the muscle is challenged with repeated injuries, *BMI1*^*−*/*−*^ mice display a compromised and delayed regeneration with defects in the maturation of the regenerating fibers (Robson et al., 2011). The phenotype observed in *BMI1*^*−*/*−*^ mice is similar to that observed when EZH2 is ablated specifically in the satellite cells (Juan et al., 2011, Woodhouse et al., 2013). The role of both EZH2 and BMI1 has been investigated also in the murine dystrophic environment. Tumor necrosis factor-induced downregulation of Notch levels, a key molecular mechanism in muscle regeneration, is mediated via recruitment of EZH2 on a specific region of the *Notch1* gene, with subsequent increased trimethylation of K27, both *in vitro* and *in vivo* (Bhattacharyya et al., 2009, Palacios et al., 2010). In the *Mdx* mouse, BMI1 is downregulated in the satellite cells (Di Foggia et al., 2014) and *Mdx*;*BMI1*^*−*/*−*^ show fewer and smaller centrally nucleated fibers in the diaphragm (Robson et al., 2011), a muscle known to be particularly affected by chronic muscle wasting and repeated cycles of repair due to its essential role in respiration (Robson et al., 2011). In genetically engineered mouse models, BMI1 overexpression in the satellite cells increases the Pax7^+^/MyoD^−^ proportion of the satellite cells *in vitro*, without impairing their differentiation potential (Di Foggia et al., 2014). Mild overexpression of BMI1 specifically in the satellite cells enhances the muscle strength of *Mdx* mice after challenge with treadmill exercise for 6 weeks (Di Foggia et al., 2014).

Here, we show a reduced number of cells expressing BMI1in both quiescent and committed DMD myoblasts with its expression level also reduced in these cells. These findings raise the possibility that BMI1 depletion contributes to the impaired regenerative process also in the human dystrophic context. We show here that upregulation of BMI1 expression in human DMD myoblasts ameliorates their differentiation potential, an effect even more pronounced than in normal myoblasts. While BMI1 overexpression increases the proliferation of the normal myoblasts, this effect is not noted in DMD myoblasts, where a decrease in proliferation is observed instead. As the basal proliferation rate of the DMD cultures was significantly lower in comparison with normal cells, it is possible that their proliferation capacity is already on a plateau because of cellular exhaustion and cannot be further increased. However, this did not impact on the enhanced differentiation induced by BMI1 overexpression in human DMD myoblasts.

The connection between mitochondrial processes and stem cell function has been recently highlighted (Garcia-Prat et al., 2016). Bmi1 plays a well-defined role in maintaining mitochondrial function and redox homeostasis in various stem cell populations, hence linking regulation of cellular metabolism with stem and progenitor cell function (Liu et al., 2009). We show here an enhanced energetic state with increased ATP production upon overexpression of BMI1 in human DMD myoblasts both *in vitro* and *in vivo* upon transplantation of these cells into a dystrophic environment. *Sgca-null*/*scid*/*bg* mice (Tedesco et al., 2012) were chosen as recipient as this is a more severe dystrophic model compared with the *Mdx* mouse with significant fibrosis, and would therefore provide a more challenging environment for the transplanted DMD myoblasts. Interestingly, the morphological substrate for the increased ATP production was shown to be the higher number of MyHC IIX fast fibers originating from the DMD BMI1^Over^ myoblasts engrafted into the dystrophic TA muscles.

Increased ATP levels were also observed in *Mdx* mice where Bmi1 is overexpressed in Pax7^+^ satellite cells, similarly to that observed in the xenograft model. Importantly, despite the enhanced energetic state, a significant functional impact with amelioration of the performance of the dystrophic muscles under a forced exercise regime was found only when concomitant reduction of DNA damage occurred, an event which was dependent on metallothionein 1-mediated protection from oxidative stress-induced cellular damage.

We confirmed upregulation of *MT1* in human DMD myoblasts upon BMI1 overexpression in a genome-wide expression analysis. Interestingly, we also found upregulation of *PRDX2* in these cells. PRDX2 belongs to the peroxiredoxin family, which are thioredoxin-family antioxidant enzymes that scavenge cellular peroxides and contribute to redox homeostasis (Rhee and Woo, 2011, Winterbourn and Hampton, 2015). Functionally we observed an enhanced scavenging role and increased protection from DNA damage in DMD BMI1^Over^ myoblasts compared with control DMD cells, as assessed by increased GSH/GSSG ratio and a significantly lower intensity of γH2AX foci, respectively, both *in vitro* and *in vivo* in xenografts. Importantly, we show that PRDX2 mediates at least in part the effect of BMI1^Over^ on the energetic state of the cells, as treatments with PRDX2 inhibitors neutralized the increased ATP production induced by BMI1^Over^ in DMD myoblasts. These data are well in keeping with our previous observation in genetically engineered mouse models (Di Foggia et al., 2014).

In conclusion, our preclinical data in mouse models support the development of pharmacological approaches to target BMI1-mediated mitochondrial regulation and protection from DNA damage as a novel therapeutic approach to stimulate endogenous, but possibly also transplanted, satellite cell self-renewal to sustain and enhance their contribution to muscle regeneration in DMD patients. Importantly, we show that BMI1 expression is reduced in conditions of chronic muscle wasting independently of their etiology, hence raising the possibility that the patient benefit of such an approach may be wider than originally predicted.

## Experimental Procedures

### Human Biopsies

Surplus material of muscle biopsies performed at Barts Health NHS in the context of the clinical evaluation of patients with neuromuscular disorders was used. Biopsies of DMD patients and age-matched controls were obtained from the MRC Center for Neuromuscular Diseases Biobank London (REC 06/Q0406/33) (R. Phadke and F. Muntoni). The use of human muscle samples was in agreement with the UK Human Tissue Act 2004, and its use for this specific research had ethical approval (East London and The City REC Alpha, ReDA Reference: 006244). All patients or their legal guardians gave written informed consent.

### Human Myoblast Cultures

Immortalized and non-immortalized human myoblasts (isolated from DMD and histologically normal muscle biopsies, Table S1) were obtained from the MRC Center for Neuromuscular Diseases Biobank (Mamchaoui et al., 2011) and used in this study under the same ethics stated above. Cells were maintained in Skeletal Muscle Medium plus supplements (Promocell Kit, C-23060), 10% fetal bovine serum (PAA A15-151), 1× GlutaMAX (Invitrogen 35050038), and 5 μg/mL gentamicin (Sigma G1272). Differentiation was induced at confluence by replacing the growth medium with DMEM and 2% horse serum.

### Cell Transplantation Experiments

DMD human myoblasts were infected with BMI1^Over^ and GFP lentiviral particles. Cells (1 × 10^6^) were transplanted into TA muscles (n = 6) of Sgca-null/scid/beige mice (Tedesco et al., 2012) 7 days later. After 25 days the muscles were collected, frozen in isopenthane, and mounted on Tragacanth gum (Sigma). Serial sections (8 μm thick) were cut, stained for specific antigens, and analyzed with ImageJ or Definiens AG (Developer XD, Munich). This work was conducted under UK Home Office Project Licenses no. 70/7435 and 70/8566.

### Statistical Analysis

Unless specified in the text, all the graphs represent population mean ± SD. Statistical analysis was performed with Prism statistical analysis software (GraphPad), Student's t test for single comparisons and ANOVA for multiple comparisons were applied. Significance is indicated in figures as ^∗∗∗^p < 0.001, ^∗∗^p < 0.01, and ^∗^p < 0.05.

## Author Contributions

S.D., P.K., M.N.-C., M.R., and V.d.F. conducted the wet lab experiments; S.D., L.G.R., P.K., M.N.-C., F.S.T., M.R., V.d.F., and S.M. analyzed the data; C.P.C. and M.R.B. conducted and analyzed the computational experiments; M.E. performed the image analysis; S.D., M.N.-C., F.S.T., and S.M. designed the experiments; A.R. contributed patient samples and clinical data; S.M. wrote the paper with contributions from all authors.

## Supplemental Information

Document S1. Supplemental Experimental Procedures and Figures S1–S6

Table S1. List of Human Satellite Cell-Derived Myoblasts Isolated from DMD Patients and Healthy Donors

Document S2. Article plus Supplemental Information
