# Supplementary material for: Enhanced Energetic State and Protection from Oxidative Stress in Human Myoblasts Overexpressing BMI1
Source: Stem Cell Reports. 2017 Jul 20;9(2):528–42. doi: 10.1016/j.stemcr.2017.06.009 (PMC5549966; doi:10.1016/j.stemcr.2017.06.009)
Supplement: Document S1. Supplemental Experimental Procedures and Figures S1–S6 [file mmc1.pdf]

Stem Cell Reports, Volume *9*

Supplemental Information

Enhanced Energetic State and Protection from Oxidative Stress in Human Myoblasts Overexpressing BMI1

Silvia Dibenedetto, Maria Niklison-Chirou, Claudia P. Cabrera, Matthew Ellis, Lesley G. Robson, Paul Knopp, Francesco Saverio Tedesco, Martina Ragazzi, Valentina Di Foggia, Michael R. Barnes, Aleksandar Radunovic, and Silvia Marino

Dibenedetto et al. Figure S1

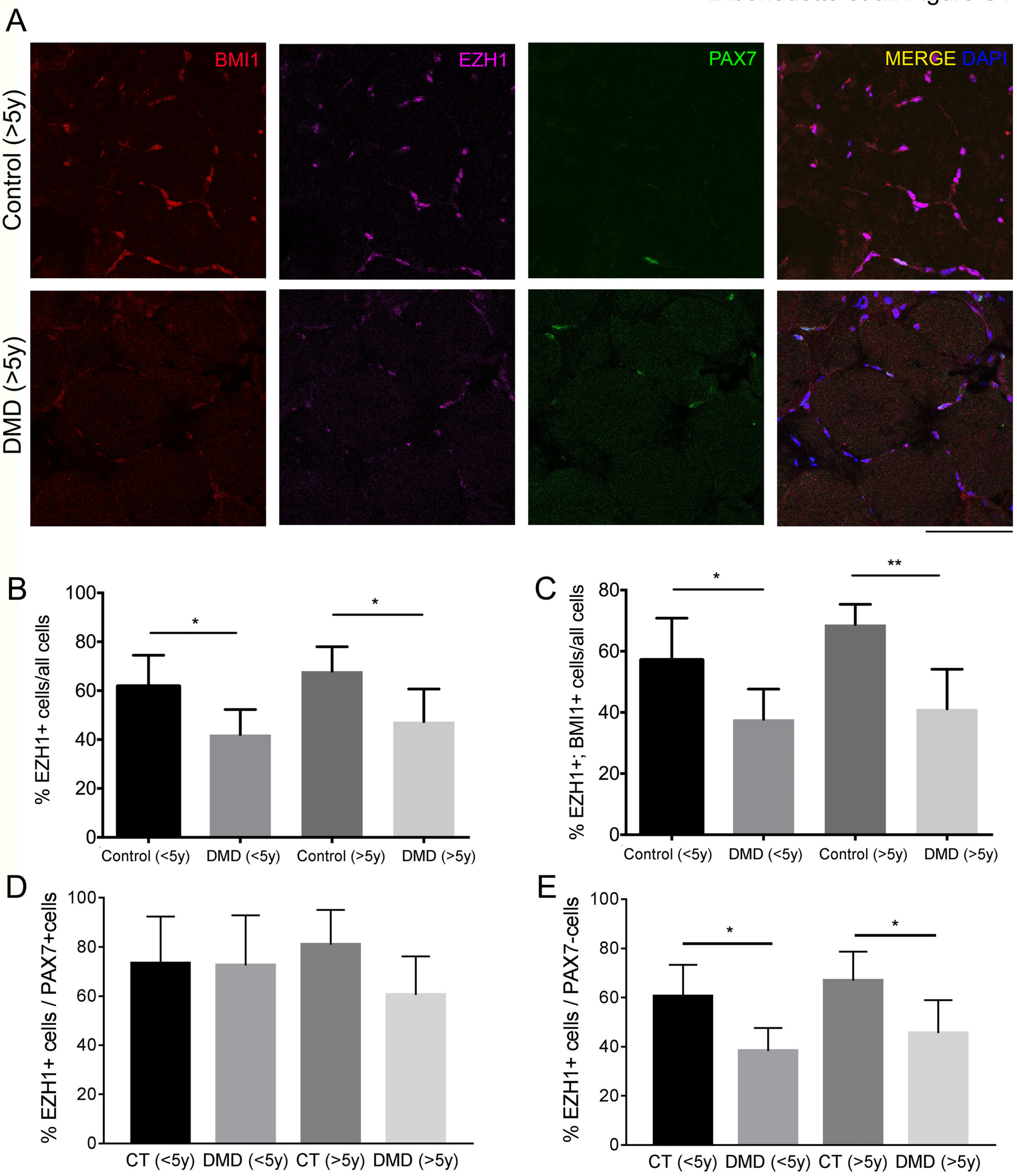

Dibenedetto et al. Figure S2

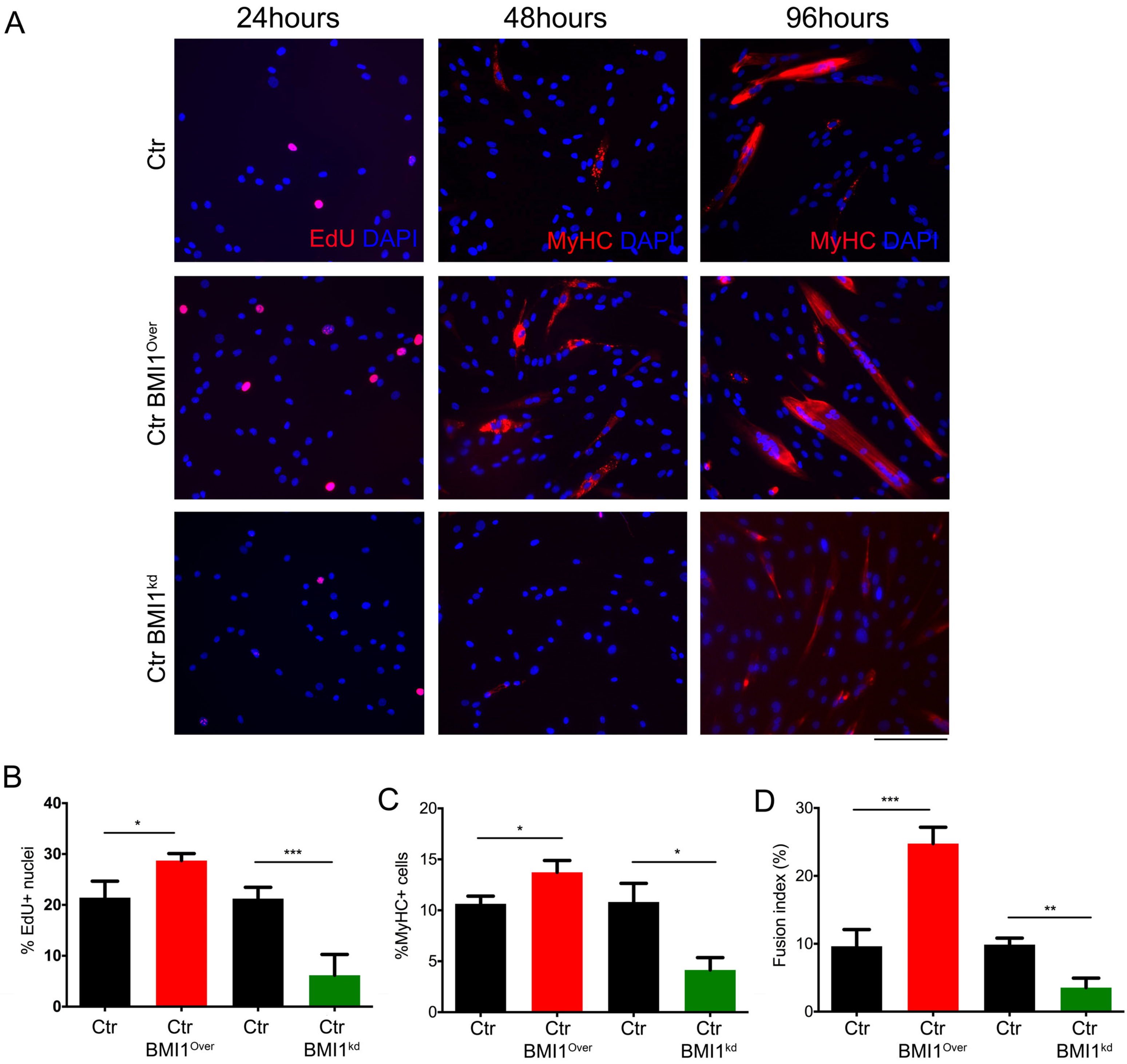

Dibenedetto et al. Figure S3

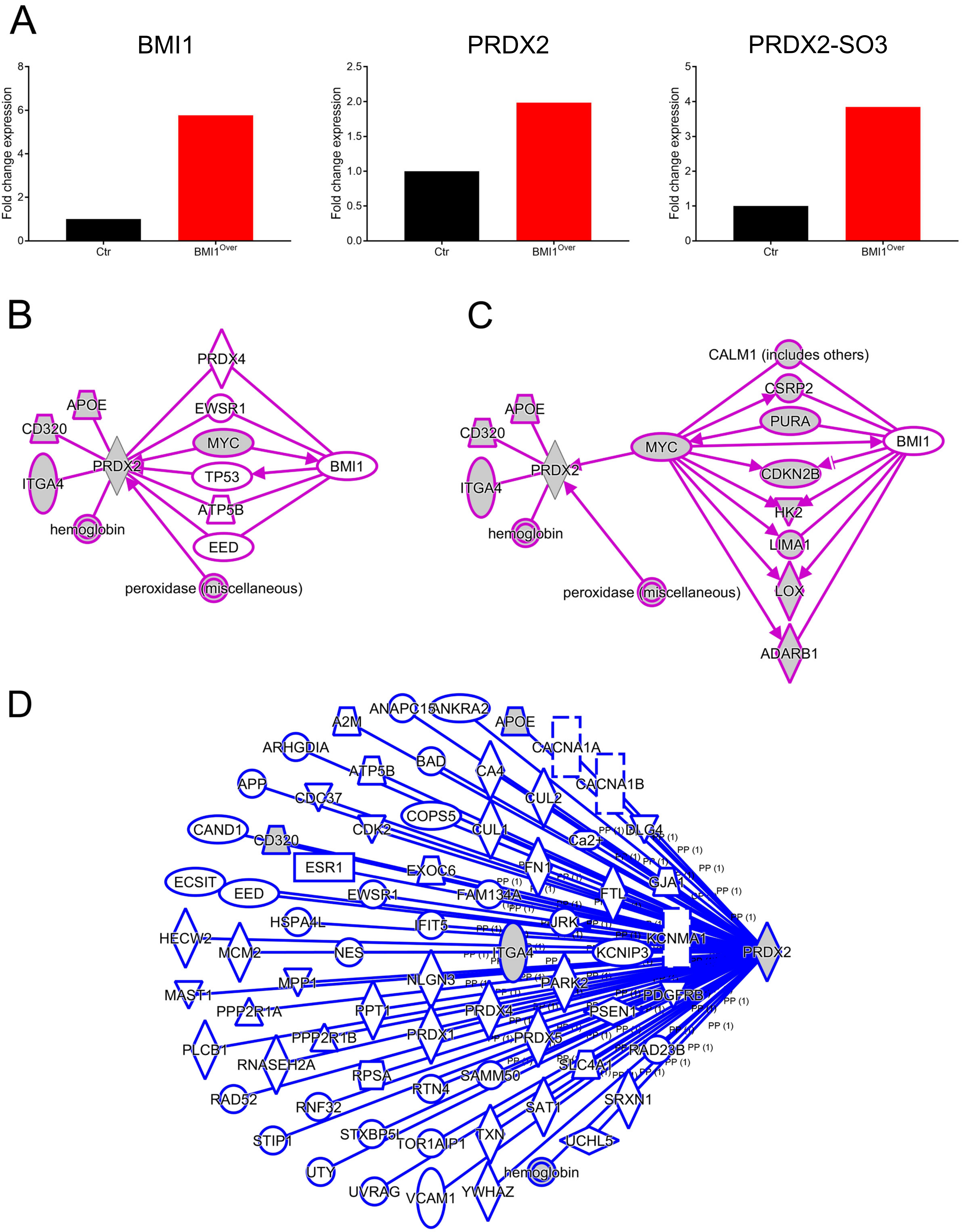

Dibenedetto et al. Figure S4

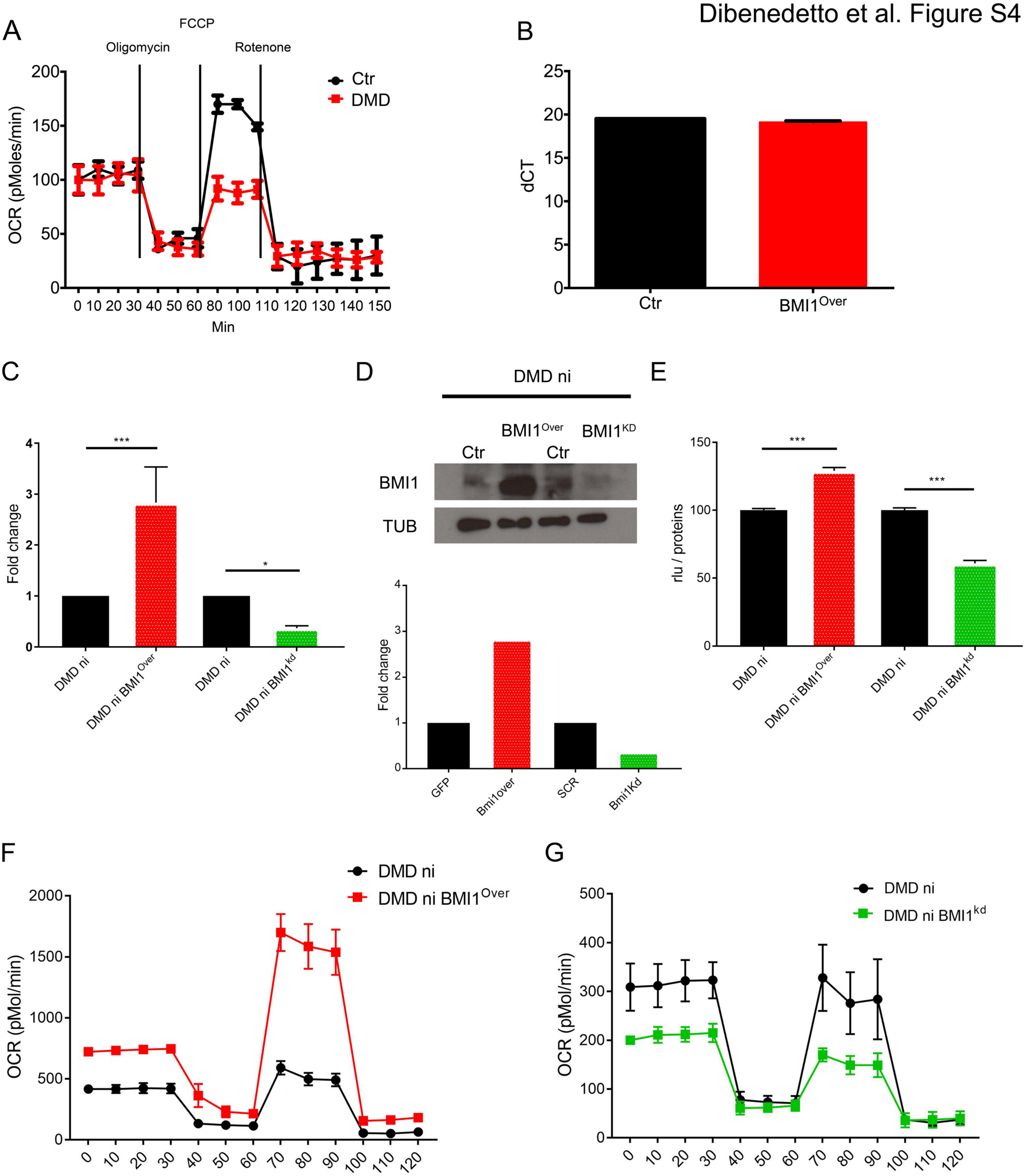

Dibenedetto et al. Figure S5

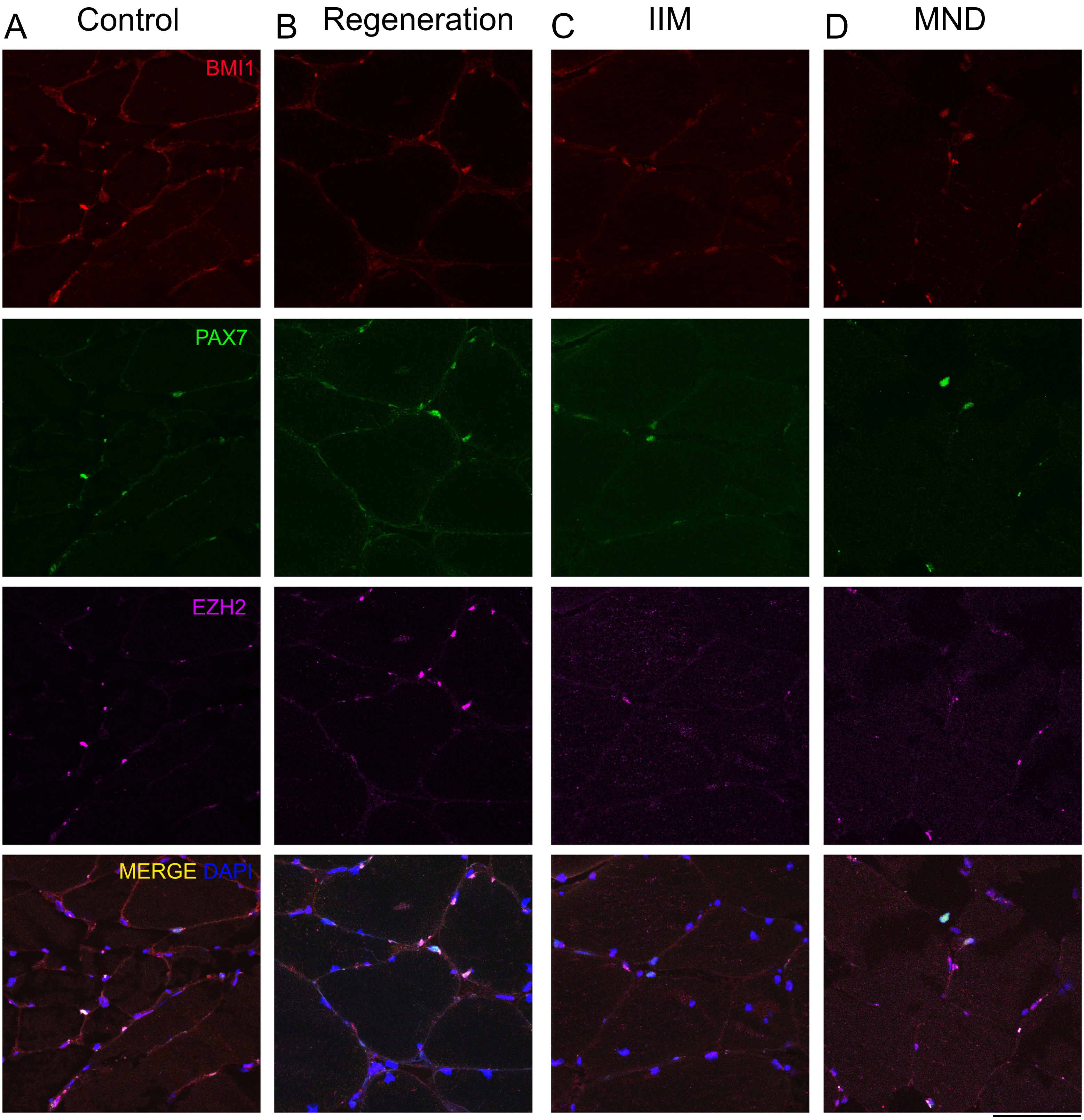

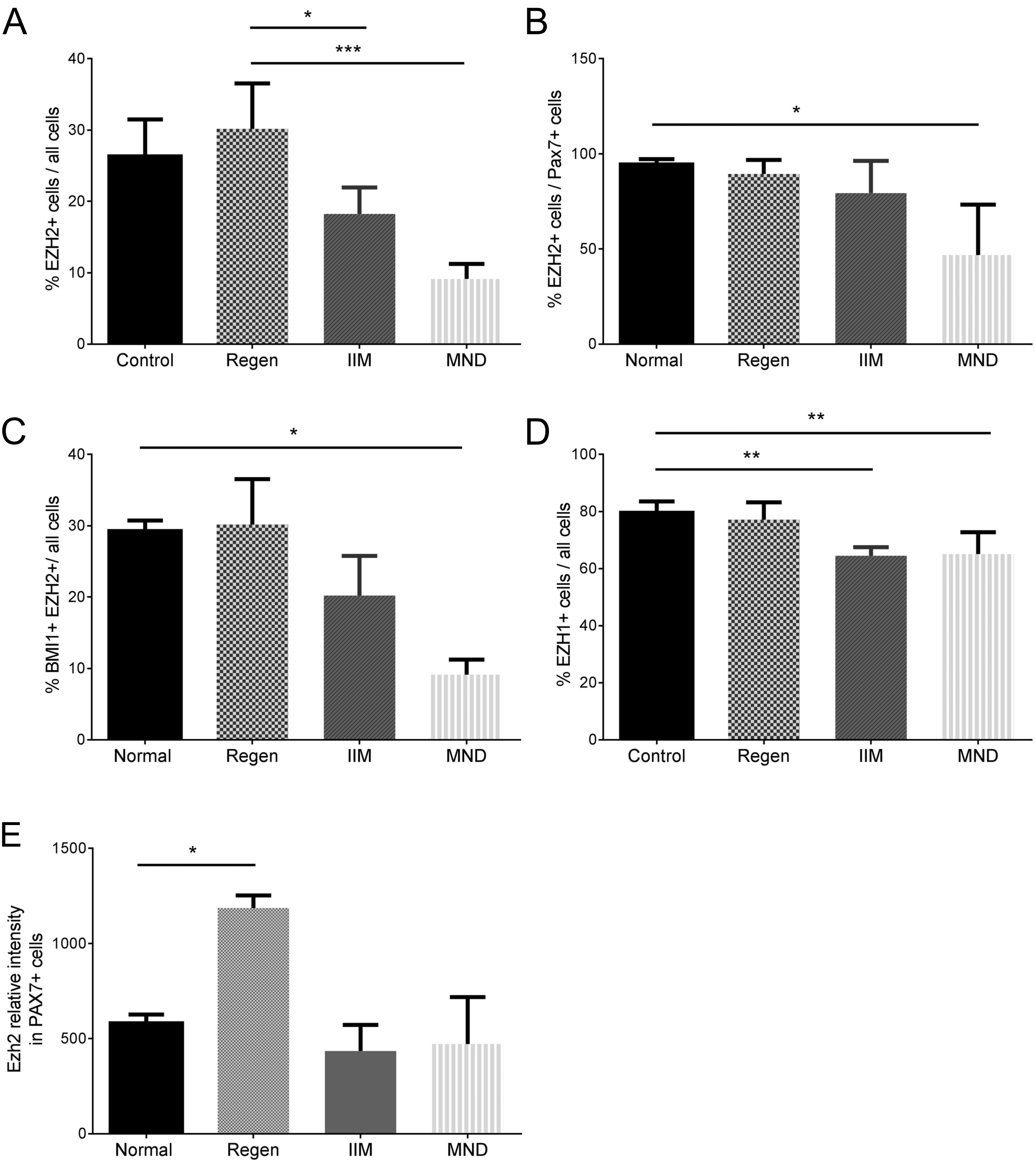
A
% EZH2+ cells / all cells
0
10
20
30
40
*
***
Control
Regen
IIM
MND
B
% EZH2+ cells / Pax7+ cells
0
50
100
150
*
Normal
Regen
IIM
MND
C
% BMI1+ EZH2+/ all cells
0
10
20
30
40
*
Normal
Regen
IIM
MND
D
% EZH1+ cells / all cells
0
20
40
60
80
100
**
**
Control
Regen
IIM
MND
E
Ezh2 relative intensity
in PAX7+ cells
0
500
1000
1500
*
Normal
Regen
IIM
MND

Supplementary Figure Legends

Fig S1.Depletion of EZH1+ cells in DMD patients.

A) Triple immunostaining for BMI1, EZH1 and PAX7 on frozen muscle transverse sections of DMD patients (n=4 patients <5 year old; n=4 patients >5 year old) and aged matched controls (n=4 patients <5 year old; n=4 patients >5 year old). Representative images of the staining on >5 year old DMD and control muscles are shown. B) Quantification of EZH1+ cells over the total number of nuclei. C) Quantification of BMI1+;EZH1+ cells over the total number of nuclei. D) Quantification of EZH1+ cells over the total number of PAX7- cells and E) PAX7+ cells (mean ± SD; *, p <0.05; **, p<0.01). Quantification of the number of +ve cells was carried out on at least 5 high power fields (40x) for each case. Scale bar is 125μm

Fig S2. Increased proliferation and differentiation in normal human myoblasts overexpressing BMI1.

Control human myoblasts are infected with a *GFP* or *BMI1* encoding lentiviral particles and with a SCR or *BMI1*-shRNA lentiviral particles. (A) Representative images of EdU and MyHC staining on human myoblasts in proliferation medium, 48 and 96hrs after induction of differentiation. Quantification of the percentage of positive cells for EdU (B) and MyHC over total number of nuclei at 48hrs is shown in (C). Differentiation rate at 96hrs after induction of differentiation is expressed as percentage of fusion index (D) (mean ± SD of three independent experiments; *, p<0.05 **, p<0.01; ***,p<0.001). Scale bar is 250μm.

Fig. S3. Predicted molecular networks in BMI1^{Over} myoblasts.

(A) Quantification of the band intensity of western blot shown in Fig.3D. (B-C) Predicted networks linking BMI1 and PRDX2, genes in grey are deregulated in our dataset. (D) Network of PDRX2 downstream target genes, genes in grey are deregulated in our dataset.

Fig S4. BMI1 overexpression has no impact on mitochondrial biogenesis and it enhances mitochondrial respiration and ATP production in non immortalised DMD myoblasts.

(A) Myoblasts control (black) and DMD myoblasts (red) were seeded in a Seahorse XF-24 analyzer and real-time OCR was determined during sequential treatments with oligomycin (ATP-synthase inhibitor), FCCP (mitochondria uncoupler) and rotenone (ETC inhibitors). A normalization of data generated by the XF24 was performed to observe the loss of the spare respiratory capacity (SRC). Data are representive of three independent experiments in which each data point represents the mean ± SD of 12 replicates for each condition.. (B) qRT-PCR showing the level of mtDNA copy number in DMD myoblasts upon BMI1 overexpression (red) as compared to the DMD control (black) (mean ± SD of three independent preparations;). Non immortalised DMD myoblasts are infected with a GFP (black bar) or BMI1 (red bar) encoding lentiviral particles and with a SCR (black bar) or BMI1-shRNA (green bar) lentiviral particles. Level of expression of BMI1 upon BMI1 overexpression or BMI1 knockdown was assessed by qRT-PCR (C) (mean ± SD of three independent preparations; *, $p<0.05$; ***,$p<0.001$) and by Western Blot (D), quantification of band intensity is shown (E). (F) ATP content measured with a luciferase assay and normalised against protein concentration in DMD ni GFP (black bar) or DMD ni $BMI1^{Over}$ (red bar) and DMD ni SCR (black bar) or DMD ni Bmi1kd (green bar) myoblasts (rlu/prot) (mean ± SD of three independent experiments;

***,p<0.001). (G,H) Real-time OCR determined with XF24 on DMD ni upon BMI1 overexpression (F) or BMI1kd (G) and compared to respective controls. Data are representive of three independent experiments in which each data point represents the mean ± SD of 6 replicates for each condition

Fig S5. Characterisation of EZH2 expression in human chronic neuromuscular disorders.

Representative images of a triple immunostaining for BMI1, PAX7 and EZH2 on frozen muscle transverse sections from morphologically normal muscle (n=6), normal muscle biopsies with evidence of regeneration (n=5), Idiopathic Inflammatory Myopathy (IIM) (n=5) and motorneurone disease (MND) (n=4). Scale bar is 125μm.

FigS6. Reduction of EZH2 and EZH1 expression in human chronic neuromuscular disorders.

(A) Quantification of EZH2+ cells over the total number of nuclei and (B) amongst PAX7+ satellite cells. (C) Percentage of BMI1, EZH2 double positive cells over the total number of nuclei. (D) Percentage of EZH1+ cells over the total number of nuclei. (mean ± SD; *, p<0.005; **, p<0.001; ***, p<0.0001). Quantification of the number of +ve cells was carried out on at least 5 high power fields (40x) for each case.

Supplementary Experimental Procedures

Production of BMI1over and shRNA-BMI1 lentiviral vector

pLox-CWBmi1 (#12240) and pLox-CWGFP (#12241) (used as control) (both from Addgene) lentiviral vectors were used to assess the impact of BMI1 overexpression in the myoblasts culture. A pGIPZ-lentiviral shRNA vector containing a hairpin sequence targeting *BMI1* (ThermoFisher, Clone Id: V3LHS_302124) was used to achieve *BMI1* knock-down. A pGIPZ lentiviral vector containing a scramble sequence was used as control. The lentiviral vectors were co-transfected with lentiviral packaging, using the calcium phosphate method into 293T cells. The medium was refreshed and viruses were harvested 48 hours after transfection, passed through 0.45-µm filters, concentrated by PEG precipitation, and stored at -80°C. The infectious titer was determined by FACS analysis of GFP positive 293T cells.

Lentiviral mediated BMI1overexpression and knockdown

Human myoblasts were seeded in multiple well plates and infected with BMI1 and GFP control (10 MOI) or shBMI1 and Scramble (30 MOI) lentivirus in proliferation medium. The medium was replaced on the second day and the transduction efficiency was analysed ninety-six hrs after infection by qPCR and WB.

Immunohistochemistry

All myoblast cultures and muscle sections were fixed with 4% PFA for 10 min.

For immuno-labelling primary antibodies were applied for one hour or overnight at room temperature or 4°C respectively for myoblasts or muscle section respectively; appropriate fluorescent secondary antibodies were used and the myogenic cultures or sections were mounted using Vectashield mounting medium with DAPI (Vector Laboratory).

Peroxidase-based immunohistochemistry

Sections were air dried and then blocked with normal horse serum for 10 min. (Vectastain kit). For the detection of fibers of human origin, sections were incubated for 1hour at RT with hSpectrin (1:100, VP-5283, Vector Laboratories) and hLamin A/C (1:100, NCL-LAM-A/C, Novacastra), followed by 30 min incubation in universal biotinylated serum (Vectastain). Sections were then treated for 20 min with Elite ABC complex (Vectastain) and for 10 min with liquid DAB (BioGenex). Finally sections were counterstain in Gill's haematoxylin, digitised to give 8-bit colour images at x40 magnification using a Leica SCN400F whole slide scanner, and the number and CSA of engrafted fibers was analysed with Definiens Developer.

Tissue areas were identified based on the lowest (darkest) pixel value at each pixel from the composite RGB image layers. The mean pixel value from these 'darkest' pixels was calculated and adjusted by -15 (this ensures only stained tissue is selected) was then used to separate tissue from background. The blue and brown components of the images were then identified using the HSD model (van Der Laak et al., 2000) which provides a direct measure of each colour. Brown regions were identified as spectrin, then spectrin regions were grown into intensely blue regions to close gaps in sarcolemma identification. Regions of positive fibres were then manually selected.

A combination of morphological measurements and object cutting processes were then used to optimise the identification and separation of positive fibres and their associated nuclei. Some nuclei associated with the sarcolemma have a high degree of overlap with the sarcolemma so cannot be identified as separate objects. These nuclei were inferred from the presence of a relatively dark spots associated with a

widening of the sarcolemma that satisfy inclusion criteria. The area and width was then exported for each fibre.

Antibodies

The following primary antibodies were used: goat anti-BMI1 (Santa Cruz Biotechnology, sc-8906,1:100), Click-iT TM EdU Imaging Kits (Invitrogen, C-10340), rabbit anti-Laminin (Sigma-Aldrich, L-9393, 1:1000), rabbit anti-Myf5 (C-20) (Santa Cruz Biotechnology, sc-302, 1:100), mouse anti-pan myosin heavy chain (A4.1025), mouse anti myosin heavy chain type1(BA-F8), mouse anti myosin heavy chain type IIA (SC-71), mouse anti myosin heavy chain type IIX (6H1) (all from Developmental Studies Hybridoma Bank, 1:10), mouse anti-Pax7 (Developmental Studies Hybridoma Bank, 1:1), Rabbit anti-alpha sarcoglycan (EPR14773) (Abcam, ab189254,1:200), rabbit anti-γH2AX (phosphor S139)-DNA double-strand break marker (Abcam, ab11174,1:200), mouse anti-hSpectrin (Vector Laboratories, VP-5283, 1:100), mouse anti-hLamin A/C (Novacastra, NCL-LAM-A/C, 1:100), rabbit anti-Ezh1 (Millipore, ABE281, 1:100), rabbit anti-Ezh2 (Cell Signalling, D2C9,1:100). The following secondary antibodies were used: donkey anti-mouse IgG Alexa 488 (R37114, 1:1000), goat anti-mouse IgG1 Alexa 546 (A-21123, 1:1000), goat anti-mouse IgG1 Alexa 488 (A-21121, 1:1000), goat anti-mouse IgG2b Alexa 555 (A-21147, 1:1000), goat anti-mouse IgM Alexa 488 (A-21042, 1:1000), donkey anti-rabbit IgG Alexa 488 (A-21206, 1:1000), donkey anti-rabbit IgG Alexa 647 (A-31573, 1:1000), donkey anti-rabbit IgG Alexa 546 (A-10040, 1:1000), donkey anti-goat Alexa 568 (A-11057, 1:1000) (all from ThermoFisher Scientific).

Microscopy and quantification

Fluorescent and bright field image capture was performed using a Leica epifluorescent microscope or Zeiss Meta 510 LSM or Zeiss LSM 710 confocal. In

myoblast cultures, all DAPI positive cells and antigen positive cells were counted and the percentage of Antigen$^+$ cells was calculated. Data from ≥3 cultures were pooled to give a population mean ± SD

For the quantification of the intensity staining all samples were stained simultaneously and the pictures were acquired with a Zeiss LSM 710 confocal at the same ratio of emissions. For the analysis in ImageJ software, the shape of each Pax7$^+$ cell and hLamin$^+$ cells was drawn around the nucleus and the intensity of antigen staining calculated for each cell as mean of Integrated Density (Mean Intensity*Area of the cell). The average Integrated Density was calculated among all cells per each field acquired.

Measurement of cellular oxygen consumption rate and extracellular acidification rate

An extracellular flux (XF) analyzer (Seahorse Bioscience, North Billerica, MA) was used to determine metabolic phenotype of cells. XF Analyzer simultaneously monitors oxygen consumption rate (OCR) and extracellular acidification rate (ECAR), which are indicators of mitochondrial respiration and aerobic glycolysis (lactate production), respectively, of cultured cells.

XF-24 cell culture microplates were coated with 3.4 mg/mL BD Cell-TakTM tissue adhesive solution (BD Bioscience 354240) according to manufacturer’s instruction. 43µl of the Cell-Tak solution was added to each well of a XF-24 cell culture plate and incubated for 20 min a room temperature.

Immortalised myoblasts were plated at densities of 50,000 cell per well in XF-24 cell culture plates and incubated overnight in 37°C incubators.

Non immortalised myoblasts (50,000 cell per well) were attached to Seahorse XF-24 plates pre-coated with Cell-TAK on the same day of the analysis. Microplates

containing the cell suspension were centrifuged at 700×g for 5 min and then incubated at 37°C for at least 1h to allow attachment.

Before measurements, the growth medium was replaced with 600 μl assay medium (Seahorse Bioscience), a low buffered DMEM containing no bicarbonate, and incubated for 45 minutes in a 37°C non-CO2 incubator. Basal oxygen consumption rate and extracellular acidification rate was determined using XF24 Extracellular Flux analyzer. Oxygen-consumption rate (OCR) was determined in response to sequential treatment with the ATPase inhibitor oligomycin, the uncoupling agent FCCP and the electron-transport-chain inhibitors rotenone. Oligomycin, carbonyl cyanide p-trifluoromethoxyphenylhydrazone (FCCP) and rotenone were obtained from Sigma, and stock solutions were prepared following the manufacturer's instruction.

ATP measurement

Human myoblasts were plated at a density of 60,000 cells/well in a 6 well plate. Cells were harvested 24hrs later by centrifugation at 1000rpm 180 g for 5 min to remove extracellular ATP. Harvested cells were suspended in 100μL cold water and sonicate for 5 min. Next, 50 μL of cell suspension was mixed with 50μL CellTiter-Glo Luminescent Cell Viability Assay solution (Promega, Madison, WI, USA), and incubated for 10 min. The luminescence intensity was measured using a Synergy HT Multi-Mode Microplate Reader. 2 μL of cell suspension was used to measured protein levels. All the readings were express as rlu (relative luminescence unit) /mg of proteins.

Conoidin A (Cayman Chemical) was used as inhibitor of PRDX2. Non immortalised cells were treated with 2uM of Conoidin A for 24 hours at 37ºC. ATP level was measured as described above and compared to DMSO treated control cells.

For ATP measurement on mouse muscles, sections were scrapped off the slides and resuspended with 100 µl of cold PBS and then processed as described above.

GSH/GSSG ratios

Cells were plated in a 96 well plate at 5,000 cells per well density the day before the test. Reduced and oxidized Glutathione ratio was measured by using the GSH/GSSG-Glo assay kit (Promega) according to the manufacturer's protocol. Assay reagents were added directly to cells cultured in the multiwell plates. The GSH/GSSG-Glo™ Assay is a luminescence-based system for the detection and quantification of GSH/GSSG ratios in cultured cells. The luminescence intensity was measured using a Synergy HT Multi-Mode Microplate Reader and normalised against protein concentration.

DNA damage measurement

Cells were treated for 10 min at RT with 50µM H_2O_2. Medium was then replaced with fresh one and cells were incubated for 45 min in a 37ºC incubator to allow for DNA repair process to occur. Cells were then washed with PBS, fix for 10 min with 4% PFA and then immunostained with γH2AX antibody. Cell images were acquired using an InCell 1000 automated microscope (GE), and then analysed using InCell Developer Toolbox software (GE) to determine the mean area of foci and mean intensity of the staining. Data were averaged for the triplicate technical replicates and compared to the untreated wells.

DNA, RNA extraction and qPCR analysis

DNA extraction was performed using the DNeasy Blood and Tissue kit (Qiagen). RNA extraction was carried out using the RNeasy Micro purification kit (Qiagen) and DNase digestion was performed to remove genomic DNA. cDNA synthesis was performed with SuperScript III Reverse Transcriptase Kit (Invitrogen). qRT-PCR

analysis was performed with Taqman assays (AB Applied Biosystems) with FAM labeled probes in 96 well plates using the Applied Biosystems 7500 RT PCR machine according to the manufacturer’s instructions (*BMI1*:Hs00180411_m1; *MT1*: Hs01938284_g1; *PRDX2*: Hs00853603_s1; *GPX3*: Hs01078668_m1; *MT-ND1*: Hs02596873_s1). The cDNA content was normalized against the expression of the housekeeping gene *GAPDH* (Hs02758991_g1) (technical duplicates for each culture and ≥3 independently derived cultures or biological samples were analysed).

Western blot

Myoblasts were lysed for one hour on ice with RIPA buffer (1% Igepal CA-630, 2 M TrisHCl pH 8.0, 0.5% Na-deoxycholate, 0.1% SDS, 2 mM EDTA, 150 mM NaCl including Mini protease inhibitor complete cocktail (Sigma Aldrich, 11836153001)) followed by max speed centrifugation (10 mins). The supernatants were collected and the protein concentration was measured using BCA Protein Assay Kit (Pierce). Twenty µg of protein per lane were separated on a NuPage 4-12% Bis-Tris Gel (Invitrogen) for 2hr at 100mA and electroblotted onto nitrocellulose PROTRAN (Amersham) 1 hr at 100V. After blocking 1hr at RT in TBST buffer (25 mMTrisHCl, 137 mMNaCl, 0.1% Tween 20, pH 7.5) containing 5% skimmed milk, immunodetection of proteins was performed with mouse anti-BMI1 (Clone F6)(Millipore, 06-637, 1:2000), rabbit anti Peroxiredoxin 2 (Abcam, ab59539, 1:2000), rabbit anti SO_3 Peroxiredoxin (Abcam, ab16830, 1:2000) ON at 4º C followed by HRP-conjugated anti-mouse (NA931) and anti-rabbit (NA934) IgG secondary antibody (1:5000; Amersham for 1hr) at room temperature. Enhanced chemoluminescence (ECL Plus; Amersham) was used for detection of the bands. Mouse anti-α-tubulin (Sigma, T5168, 1:5000) and Mouse anti vinculin (Sigma, V4505, 1:5000) were used as a control for gel loading.

Proliferation and differentiation assays

Cells were plated in growth medium on 13 cm diameter coverglass coated with Matrigel (90µg/ml) in 24 well plates at 20,000 cell density per well. The day after medium was replaced with differentiation medium and proliferation rate was assessed via EdU incorporation 24hrs after induction of differentiation. Cells were treated for 2hrs 30min with 10uM EdU solution (Click-iT EdU Imaging kit, TermoFisher, C-9393), then washed with PBS and fixed with 4% PFA for 10 min. Cells were permeabilized for 20 min with 0,3% Triton-PBS and then incubated for 30 min in the dark with the Click-iT reaction cocktail (Click-iT EdU Imaging kit, TermoFisher) to detect EdU incorporation.

To evaluate the differentiation rate, cells were plated and maintained as above. Cells were fixed with 4% PFA at 48 and 96 hrs after induction of differentiation and immunostained for MyHC. Coverglass were then mounted with Vectashield mounting medium plus Dapi and pictures acquired with Leica Epifluorescent microscope. Proliferation was assessed as percentage of EdU positive nuclei over the total number of nuclei. Differentiation was assessed as percentage of MyHC positive cells (either single cells at 48hrs or myotubes at 96hrs) over the total number of cells. Fusion index was expressed as percentage of the sum of nuclei in multinucleated fibers over the total number of nuclei.

RNASeq analysis

Three independently prepared normal and DMD myoblasts cell cultures infected with GFP and BMI1^{Over} lentiviral particles and induced to differentiate for 2 days were analysed by RNA sequencing (RNASeq).

One microgram of total RNA was used for library preparation, according to the Illumina TruSeq mRNA sample preparation protocol (Illumina, San Diego). Libraries

were sequenced using the Illumina NextSeq platform and TruSeq protocol. Adaptor and poor quality sequences were removed using trim galore (v 0.3.7) software. Trimmed sequences were aligned to the human reference genome (GRCh37) using TopHat2 (v2.0.13)/bowtie2(v2.2.3). The read counts were calculated using the software HTSeq-counts. All the analyses described above were implemented using standard protocols.

Differential expression was calculated for i) differences between DMD vs DMD BMI1^{Over} and ii) differences between Ctr vs Ctr BMI1^{Over}. Genes with zero counts were removed prior to the statistical analyses. Generalized linear models from DESeq2 (Love et al., 2014) were applied to test for differential expression. To account for the paired cell lines used in the experiment between Ctr and BMI1^{Over} we included the cell line as a fixed effect in the design matrix of the linear model applied (i.e. formula(~cell + condition). Pathway analysis was performed using Ingenuity Pathway Analysis (Qiagen Redwood city, CA). Datasets are available at GEO, E-MTAB-5846.

Treadmill exercise

The dystrophic animals *mdx;*Pax7^{Bmi1}, *mdx* control littermates and C57BL/6-BL/10 mice (2-4 month) used here were those described in (Di Foggia et al., 2014). In brief, mice were injected with Tamoxifen to conditionally activate Bmi1 overexpression in satellite cells. Three days after the last injection, the treadmill exercise regime was started. Each mouse was put in a lane of the treadmill and forced to run at the speed of 12m/min for 30 min twice a week, for 6 consecutive weeks. Six days after the last treadmill session the mice were sacrificed. One forelimb was embedded in OCT and frozen in isopentane cooled at the liquid nitrogen temperature.
