# Supplementary material for: Enhanced Energetic State and Protection from Oxidative Stress in Human Myoblasts Overexpressing BMI1
Source: Stem Cell Reports. 2017 Jul 20;9(2):528–42. doi: 10.1016/j.stemcr.2017.06.009 (PMC5549966; doi:10.1016/j.stemcr.2017.06.009)
Supplement: Table S1. List of Human Satellite Cell-Derived Myoblasts Isolated from DMD Patients and Healthy Donors [file mmc2.pptx]

## Slide 1
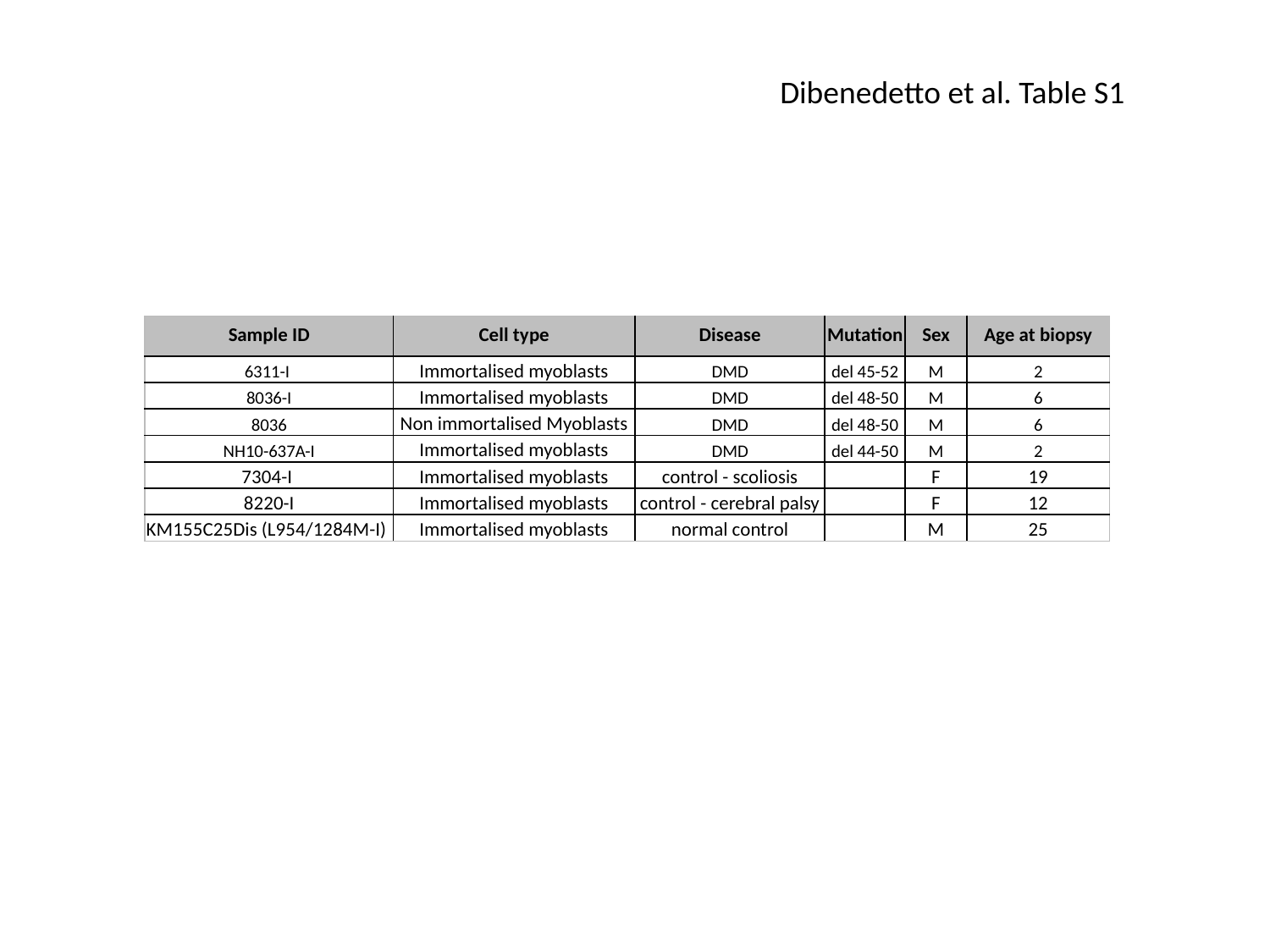

Dibenedetto et al. Table S1
| Sample ID | Cell type | Disease | Mutation | Sex | Age at biopsy |
| --- | --- | --- | --- | --- | --- |
| 6311-I | Immortalised myoblasts | DMD | del 45-52 | M | 2 |
| 8036-I | Immortalised myoblasts | DMD | del 48-50 | M | 6 |
| 8036 | Non immortalised Myoblasts | DMD | del 48-50 | M | 6 |
| NH10-637A-I | Immortalised myoblasts | DMD | del 44-50 | M | 2 |
| 7304-I | Immortalised myoblasts | control - scoliosis | | F | 19 |
| 8220-I | Immortalised myoblasts | control - cerebral palsy | | F | 12 |
| KM155C25Dis (L954/1284M-I) | Immortalised myoblasts | normal control | | M | 25 |
