# Supplementary material for: Enhanced Energetic State and Protection from Oxidative Stress in Human Myoblasts Overexpressing BMI1
Source: Stem Cell Reports. 2017 Jul 20;9(2):528–42. doi: 10.1016/j.stemcr.2017.06.009 (PMC5549966; doi:10.1016/j.stemcr.2017.06.009)
Supplement: Document S2. Article plus Supplemental Information [file mmc3.pdf]

Stem Cell Reports
Article
OPEN ACCESS

# Enhanced Energetic State and Protection from Oxidative Stress in Human Myoblasts Overexpressing BMI1

Silvia Dibenedetto,[1] Maria Niklison-Chirou,[1] Claudia P. Cabrera,[2] Matthew Ellis,[3] Lesley G. Robson,[1] Paul Knopp,[1] Francesco Saverio Tedesco,[4] Martina Ragazzi,[4] Valentina Di Foggia,[1] Michael R. Barnes,[2] Aleksandar Radunovic,[5] and Silvia Marino[1,*]

[1]Blizard Institute, Barts and The London School of Medicine and Dentistry, Queen Mary University of London, 4 Newark Street, London E1 2AT, UK
[2]Centre for Translational Bioinformatics, William Harvey Research Institute, Barts and The London School of Medicine and Dentistry, Queen Mary University of London, London EC1M 6BQ, UK
[3]Division of Neuropathology, the National Hospital for Neurology and Neurosurgery, Queen Square, London WC1N 3BG, UK
[4]Department of Cell and Developmental Biology, University College London, 21 University Street, London WC1X 0JS, UK
[5]Neuroscience and Trauma Centre, Barts Health NHS Trust, Whitechapel, London E1 1BB, UK
*Correspondence: s.marino@qmul.ac.uk
http://dx.doi.org/10.1016/j.stemcr.2017.06.009

## SUMMARY

The Polycomb group gene *BMI1* is essential for efficient muscle regeneration in a mouse model of Duchenne muscular dystrophy, and its enhanced expression in adult skeletal muscle satellite cells ameliorates the muscle strength in this model. Here, we show that the impact of mild BMI1 overexpression observed in mouse models is translatable to human cells. In human myoblasts, BMI1 overexpression increases mitochondrial activity, leading to an enhanced energetic state with increased ATP production and concomitant protection against DNA damage both *in vitro* and upon xenografting in a severe dystrophic mouse model. These preclinical data in mouse models and human cells provide a strong rationale for the development of pharmacological approaches to target BMI1-mediated mitochondrial regulation and protection from DNA damage to sustain the regenerative potential of the skeletal muscle in conditions of chronic muscle wasting.

## INTRODUCTION

Myopathies are a heterogeneous group of conditions with diverse etiologies, which affect the muscle without involving the nervous system or the neuromuscular junction. The muscular dystrophies are the most common of such disorders. However, the range of myopathies is broad, they can be not only genetic but also acquired, they are prevalent in all ethnicities, and they range widely in severity. Most are progressive in nature, often leading to muscle weakness and disability; for most there are no effective treatments or cures (reviewed in Marino and Di Foggia, 2016).

Skeletal muscle is able to regenerate throughout the life of individuals mainly due to the function of satellite cells (the main skeletal muscle stem cells [Brack and Rando, 2012; Wang and Rudnicki, 2012; Yin et al., 2013]). Satellite cells are normally quiescent and located beneath the basal lamina of the muscle fibers. After muscle injury or in diseases the satellite cells become activated and re-enter the cell cycle to provide repair cells as well as repopulating the stem cell pool (Collins et al., 2005; Hill et al., 2003; Sambasivan et al., 2011). In primary chronic myopathies, such as the muscular dystrophies, there is widespread degeneration of the skeletal muscle, and changes in the ability of the satellite cells to repair the muscle contribute to the progressive worsening of the pathology (Dumont et al., 2015; reviewed in Chang et al., 2016).

Muscular dystrophies are predominantly caused by defects in proteins that are part of the dystrophin-glycoprotein complex which form the connections between muscle cells and their surrounding cellular structure (Rando, 2001). They are currently untreatable, with many forms leading to severe disabilities and the most severe forms leading to death in early adulthood. Novel therapies are currently being developed and comprise antisense oligonucleotide-mediated exon skipping or gene replacements to restore protein expression and stem cell transplantation or a combination of both approaches (Benedetti et al., 2013). Of particular interest, the recent application of the induced pluripotent stem cell technology to derive satellite cells and myoblasts from induced pluripotent/embryonic stem cells obtained from human fibroblasts as a tool to overcome limitations in the expansion of a sufficient number of myoblasts for transplantation (Chal et al., 2015; Loperfido et al., 2015; Maffioletti et al., 2015).

Polycomb group (PcG) proteins are essential regulators of stem cell function during normal development and in adult organs (Marino and Di Foggia, 2016). They form multiprotein chromatin-associated complexes that play an essential role in the genome-wide epigenetic-mediated remodeling of gene expression during the myogenic differentiation of satellite cells, mainly through post-translational modifications of histones (Asp et al., 2011). BMI1 and EZH2 play an essential role in adult satellite cell homeostasis and proliferation in response to muscle injury, an effect


This is an open access article under the CC BY license (http://creativecommons.org/licenses/by/4.0/).

mediated at least in part by repression of the ink4a locus (Juan et al., 2011; Robson et al., 2011).

An emerging role for PcG proteins is their involvement in DNA repair (Facchino et al., 2010; Ginjala et al., 2011; Ismail et al., 2010; Liu et al., 2009; Pan et al., 2011) and in maintaining redox balance (Chen et al., 2015; Jin et al., 2014). BMI1$^{-/-}$-derived cells show significant mitochondrial dysfunction accompanied by a sustained increase in the production of reactive oxygen species (ROS) that are sufficient to engage the DNA repair pathway (Liu et al., 2009), which is in turn impaired, thus leading to magnified cellular damage. The balance between intracellular ROS and antioxidant molecules is vital in determining the rate of oxidative damage accumulation and the impaired function of satellite cells in aging and in myopathies, where decreased antioxidative capacity has been documented (Fulle et al., 2005; Tidball and Wehling-Henricks, 2007; Whitehead et al., 2006).

In Duchenne muscular dystrophy (DMD) the overall number of satellite cells is not affected, but their proliferative capacity rapidly declines during progression of the disease (Blau et al., 1983; Endesfelder et al., 2000; Gnocchi et al., 2008; Mouly et al., 2005), an effect due at least in part to their increased sensitivity to oxidative stress injury leading to reduced and defective regeneration of the muscle (Blau et al., 1983, 1985; Disatnik et al., 2000). Moreover, enzymatic adaptations to exercise-induced production of ROS and free radical damage are significantly decreased in dystrophic compared with normal muscles (Faist et al., 1998, 2001). Overall, an impaired protection against ROS in dystrophic muscle appears to contribute to disease progression, as also indicated by the beneficial effect of antioxidants in ameliorating the skeletal muscle pathophysiology in DMD patients (Whitehead et al., 2008).

We have recently shown that BMI1 is essential for efficient muscle regeneration, especially after repeated muscle injury and in a mouse model of DMD, the *Mdx* mouse (Robson et al., 2011). Conditional overexpression of BMI1 in the satellite cells of the adult skeletal muscle enhances their regenerative capacity in this model, leading to improved muscle strength. BMI1 exerts this effect, at least in part, by protecting the satellite cells from oxidative stress-induced DNA and cellular damage via upregulation of metallothionein 1 (Di Foggia et al., 2014).

Here we show that the impact of mild BMI1 overexpression observed in mouse models is translatable to human cells. In human myoblasts, BMI1 overexpression increases mitochondrial activity, leading to an enhanced energetic state with increased ATP production. Concomitantly it protects the cells from DNA damage both *in vitro* and *in vivo* upon xenografting in a severe dystrophic mouse model.

## RESULTS

### BMI1 Expression Is Reduced in Quiescent and Committed DMD Satellite Cells

We have previously shown that the expression of BMI1 is significantly reduced in quiescent satellite cells in muscle biopsies from DMD patients, a finding mirrored by the downregulation of BMI1 expression in the satellite cells of the *Mdx* mouse (Di Foggia et al., 2014). Here, we set out to further dissect the expression of BMI1 in muscle biopsies of DMD patients. To reflect the progression of the disease throughout aging, we divided patients into two groups: younger (n = 4) and older (n = 4) than 5 years old. Age-matched patients with a muscle biopsy without histological abnormalities were included in the study as controls (<5 years, n = 4; >5years, n = 4). Immunostaining for BMI1 confirmed the previously reported overall reduction of positive cells in both young and older DMD patients compared with the control group in this extended number of patients (Figures 1A and 1B). Co-immunostaining for BMI1, PAX7, and MYF5 showed that the decrease of BMI1$^{+}$cells affected both PAX7$^{+}$; MYF5$^{-}$ quiescent and PAX7$^{-}$; MYF5$^{+}$ committed myoblasts in older DMD patients (Figures 1A, 1C, and 1D), while no difference was seen in younger patients (Figures 1A, 1C, and 1D). Immunostaining for EZH1 revealed an overall reduction of the EZH1$^{+}$ cells (Figures S1A and S1B) and also of the EZH1 and BMI1 double-positive cells in both groups of DMD patients compared with their age-matched controls (Figures S1A and S1C). In this case, however, the difference was due to a reduction in the PAX7$^{-}$ population rather than in the PAX7$^{+}$ (Figures S1D and S1E).

In summary, a time-dependent depletion of quiescent and activated satellite cells expressing BMI1 but not EZH1 is noted in DMD, while an overall reduction in the myonuclei expressing BMI1 and EZH1 is detected, raising the possibility that fluctuation in the expression of BMI1 may be more relevant for satellite cells' biology.

### BMI1 Overexpression Increases Differentiation but Not Proliferation in DMD Myoblasts

Next we evaluated the impact of BMI1 modulation on human satellite cell function. Short-term cultures of human satellite cell-derived myoblasts isolated from DMD patients (n = 3) and control donors (n = 3) (Mamchaoui et al., 2011) were used for these studies. Reduced expression of *BMI1* was confirmed in DMD myoblasts compared with age-matched cultures at the RNA level (Figure 1E), in keeping with our observation in the muscle tissue (Di Foggia et al., 2014). Overexpression of BMI1 was achieved by lentiviral-mediated cell transduction and confirmed at the RNA and protein levels (Figure 2A and data not

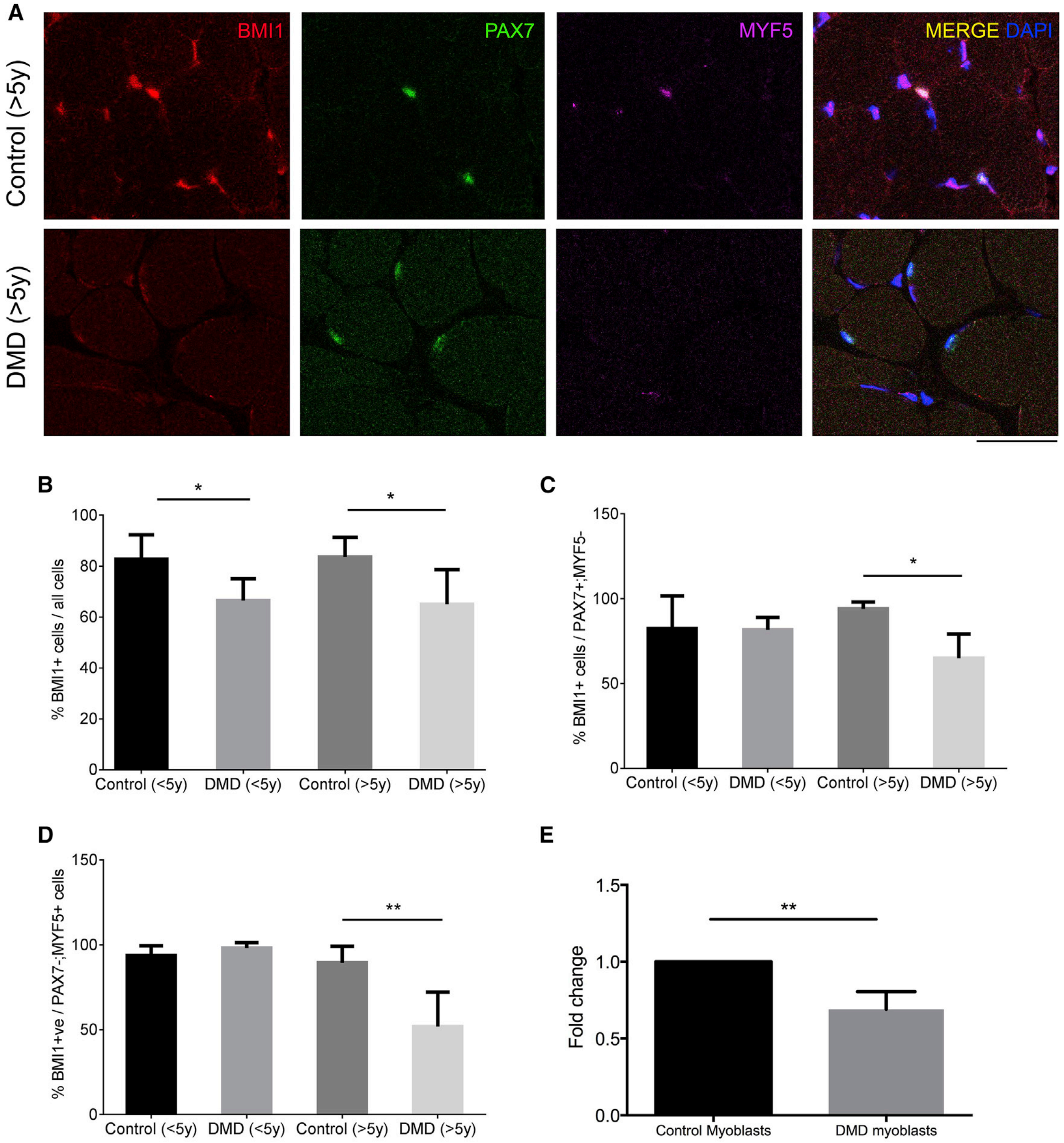


**Figure 1. Depletion of BMI1⁺ Cells in Quiescent and Committed Satellite Cells in DMD Patients**

(A) Triple immunostaining for BMI1, PAX7, and MYF5 on frozen muscle transverse sections from DMD patients (n = 4 patients <5 years old; n = 4 patients >5 years old) and age-matched controls (n = 4 patients <5 years old; n = 4 patients >5 years old). Representative images of the staining on >5-year-old DMD and control muscles are shown. Scale bar, 125 μm.

(B) Quantification of $BMI1^+$ cells over the total number of cells (mean ± SD; *p < 0.05).

(C and D) Quantification of $BMI1^+$ cells among quiescent ($PAX7^+$; $MYF5^-$) (C) and committed ($PAX7^-$; $MYF5^+$) (D) satellite cells (mean ± SD; *p < 0.05, **p < 0.01). Quantification was carried out on at least 5 high-power fields (40×) for each case.

(E) *BMI1* expression at the RNA level in DMD human primary myoblasts compared with normal human myoblasts (mean ± SD of three independent preparations; **p < 0.01).

shown). Short hairpin RNA (shRNA)-mediated knockdown of BMI1 was also performed in these cultures and its efficacy confirmed as above (Figure 2B and data not shown).

In normal myoblasts, BMI1 overexpression induced increased proliferation, as assessed by 5-ethynyl-2′-deoxyuridine (EdU) incorporation and detection 24 hr after induction of differentiation (Figures S2A and S2B).

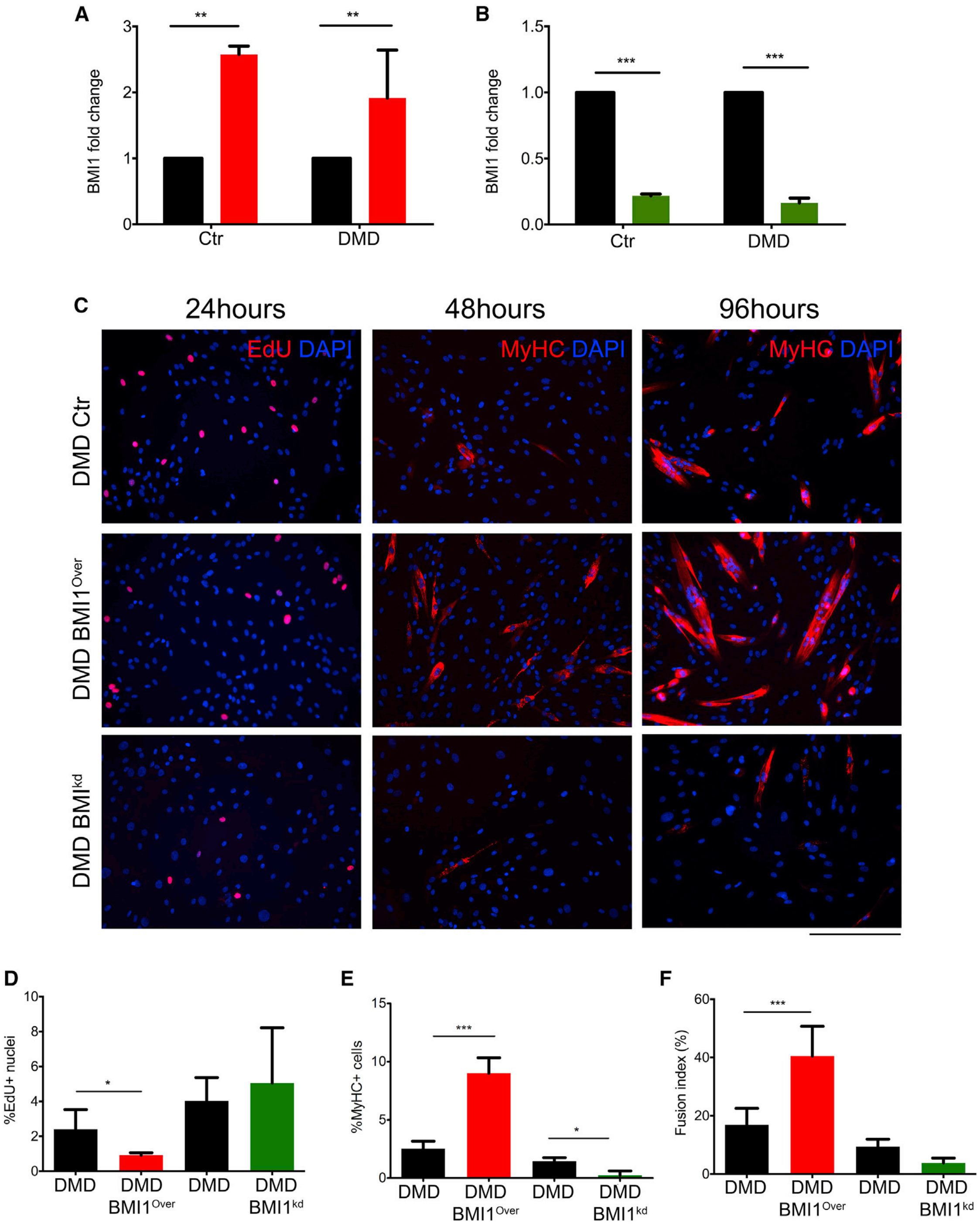


**Figure 2. Increased Differentiation in DMD Human Myoblasts Overexpressing BMI1**

(A and B) DMD and control human myoblasts are infected with a GFP (black bar) or BMI1 (red bar) encoding lentiviral particles (A) and with an SCR (black bar) or $BMI1^{-}$ shRNA (green bar) lentiviral particles (B). Level of expression of *BMI1* upon BMI1 overexpression (A) or BMI1 knockdown (B) was assessed by qRT-PCR (mean ± SD of three independent preparations; $^{**}p < 0.01$, $^{***}p < 0.001$).

(C–F) Representative images (C) of EdU staining and MyHC staining on DMD human myoblasts at 24, 48, and 96 hr after induction of differentiation. Quantification of the percentage of positive cells for EdU and MyHC over total number of cells is shown in (D) and (E). Differentiation rate at 96 hr is expressed as fusion index (F) (mean ± SD of three independent experiments; $^{*}p < 0.05$, $^{***}p < 0.001$). Quantification was carried out on at least 5 fields (20×) for each case. Scale bar, 250 μm.

To assess the impact of BMI1 overexpression on muscle differentiation, we evaluated the percentage of MyHC$^{+}$ cells and the percentage of multinucleated cells (fusion index) after 48 hr or 4 days in differentiation medium. We show that BMI1 overexpression enhances the differentiation process in normal human myoblasts, as demonstrated by the increased percentage of MyHC$^{+}$ cells (Figures S2A and S2C) and the increased fusion index leading to the formation of larger myotubes (Figures S2D).

BMI1 knockdown induced a drastic reduction of the proliferation rate (Figures S2A and S2B) and of the differentiation potential (Figures S2A–S2D), as expected if this was a BMI1-dependent effect.

Increased proliferation was not observed in DMD myoblasts upon BMI1 overexpression, with a reduced percentage of EdU$^{+}$ cells seen instead (Figures 2C and 2D). However, enhanced differentiation was noted in these cultures, as assessed by the increased percentage of MyHC$^{+}$ cells (Figures 2C and 2E) and increased fusion index (Figures 2D and 2F), an effect which was more pronounced than in normal myoblasts (9% MyHC$^{+}$ cells versus 2.5%, respectively, $p < 0.001$). BMI1 knockdown had no effect on the proliferation of the cells while reduced differentiation was observed (Figures 2C–2F).

Taken together, these data show that overexpression of BMI1 ameliorates the differentiation potential of DMD myoblasts in culture.

### Deregulation of Cellular Redox and Mitochondrial Target Genes in DMD BMI1$^{Over}$ Myoblasts and Control

To identify the downstream molecular effectors of BMI1 overexpression in myoblasts, we carried out a whole-genome transcriptome analysis on cultures isolated from three DMD patients and three age-matched controls. In particular, we set out to assess the transcriptome-wide impact of overexpression of BMI1 in DMD myoblasts compared with the same primary cell lines treated with GFP-encoding plasmid (pLoxGFP) as control (DMD versus DMD BMI1$^{Over}$). The impact of overexpressing BMI1 in control cultures (Ctr versus Ctr BMI1$^{Over}$) was also assessed, and a cross-comparison of the datasets was carried out to identify shared and condition-specific target genes.

The exploratory analysis showed 647 genes differentially expressed in DMD versus DMD BMI1$^{Over}$ (344 upregulated in DMD BMI1$^{Over}$ and 303 downregulated) and 180 in Ctr versus Ctr BMI1$^{Over}$ (71 upregulated in Ctr BMI1$^{Over}$ and 109 downregulated) when a statistical significance threshold p value of $\leq 0.05$ was applied. Comparative analysis of these two datasets revealed 54 shared target genes with 593 and 126 specific target genes for the DMD and control comparison, respectively (Figure 3A). Pathway analysis carried out on the Ingenuity platform highlighted deregulation of functions and pathways essential for cell growth and differentiation in the analyzed gene sets (Figure 3B). All stages of cholesterol biosynthesis, including the mevalonate and ketogenesis pathways, were significantly enriched in the shared gene set while calcium signaling, cyclins, and cell-cycle regulation were deregulated in the DMD comparison (Figure 3B).

Upregulation of *MT-1* was noted among the upregulated shared genes (Figure 3C), a finding consistent with previous findings in a mouse model (Di Foggia et al., 2014).

Upregulation of genes involved in maintenance of the cellular redox balance in cells, *Peroxiredoxin2* (*PRDX2*) and *Glutathione peroxidase 3* (*GPX3*), were validated in independent myoblast preparations (Figure 3C). A network connectivity analysis of BMI1 and PDRX2 revealed a predicted molecular relationship in DMD overexpressing BMI1 (Figures S3A–S3C). Importantly, overexpression of BMI1 not only increased the expression levels of PRDX2, but also of the oxidized PRDX2 form, PRDX2-SO3 (Figure 3D), indicating that BMI1$^{Over}$ myoblasts display an enhanced capacity to balance the cellular redox state and protect the myoblasts from oxidative stress.

Mitochondrial dysfunction with its links to deregulation of cholesterol biosynthesis and impairment of energy homeostasis are known to occur in DMD (Onopiuk et al., 2009; Percival et al., 2013; Rybalka et al., 2014; Yoon et al., 2016); hence, our findings raise the possibility that BMI1 overexpression ameliorates the differentiation potential of DMD myoblasts by impacting these cellular functions.

### BMI1 Overexpression Impacts on the Metabolic State of DMD Myoblast Cultures

Because myoblasts can use both oxidative phosphorylation (OXPHOS) and aerobic glycolysis as a source of energy (Paasuke et al., 2016), we measured the oxygen consumption rate (OCR), an indicator of OXPHOS, and the extracellular acidification rate (ECAR), an indicator of aerobic glycolysis, to assess the cellular bioenergetics profile of myoblasts upon BMI1 overexpression. Compounds modulating mitochondrial function (oligomycin, carbonyl cyanide-4-(trifluoromethoxy)phenylhydrazone [FCCP], and rotenone/antimycinA) were added sequentially to the cells and the effect on OCR was measured after each compound addition with a Seahorse Biosciences XF24 analyzer. Firstly, we observed a loss of spare respiratory capacity (SRC) in DMD myoblasts compared with control cells (Figure S4A), in keeping with the known mitochondrial dysfunction in DMD leading to impairment of OXPHOS with concomitant decrease of the SRC (Kuznetsov et al., 1998).

Importantly, BMI1 overexpression in DMD myoblasts led to a 33% increase in OXPHOS compared with DMD myoblasts with basal levels of BMI1 ($p = 0.0001$) (Figure 4A). An increase in aerobic glycolysis was also observed in DMD BMI1$^{Over}$ myoblasts as assessed by ECAR and OCR/ECAR

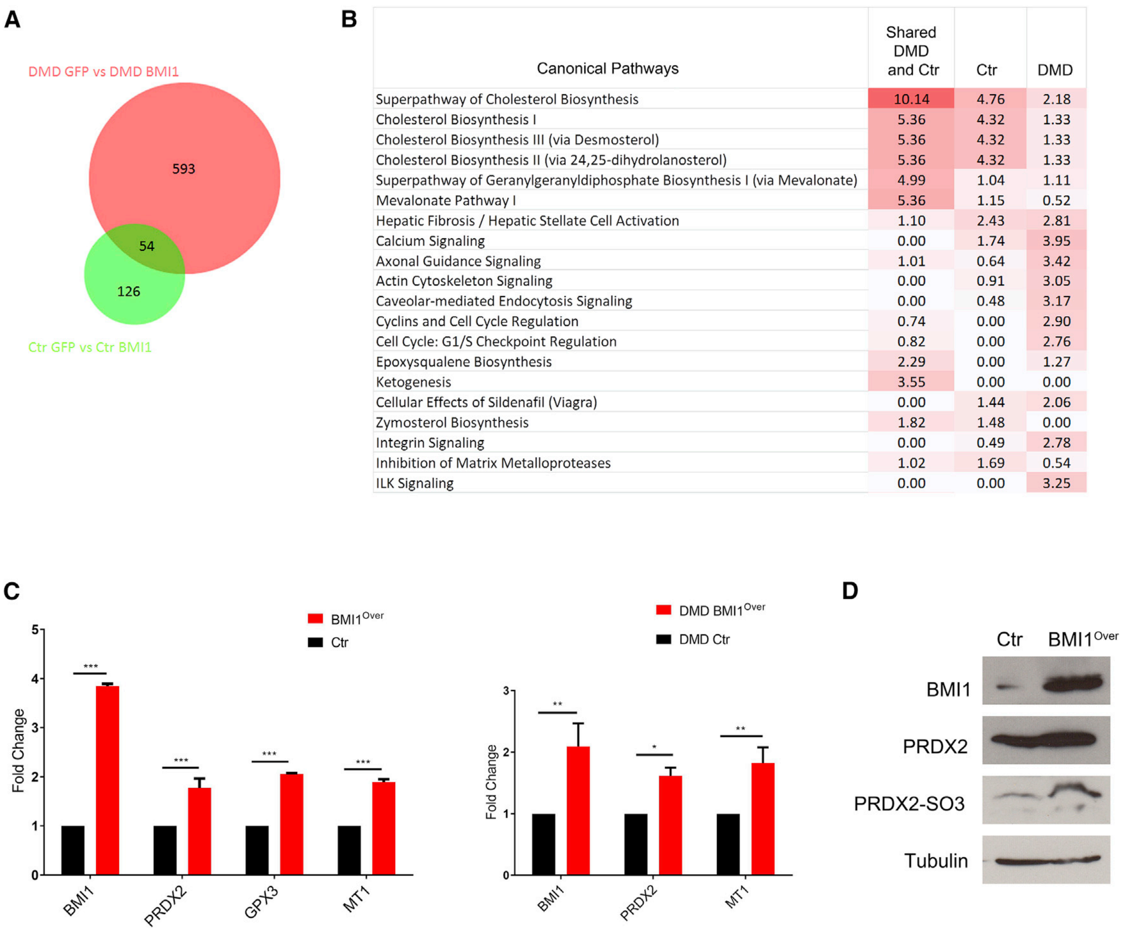


| Canonical Pathways | Shared DMD and Ctr | Ctr | DMD |
|---|---|---|---|
| Superpathway of Cholesterol Biosynthesis | 10.14 | 4.76 | 2.18 |
| Cholesterol Biosynthesis I | 5.36 | 4.32 | 1.33 |
| Cholesterol Biosynthesis III (via Desmosterol) | 5.36 | 4.32 | 1.33 |
| Cholesterol Biosynthesis II (via 24,25-dihydrolanosterol) | 5.36 | 4.32 | 1.33 |
| Superpathway of Geranylgeranyldiphosphate Biosynthesis I (via Mevalonate) | 4.99 | 1.04 | 1.11 |
| Mevalonate Pathway I | 5.36 | 1.15 | 0.52 |
| Hepatic Fibrosis / Hepatic Stellate Cell Activation | 1.10 | 2.43 | 2.81 |
| Calcium Signaling | 0.00 | 1.74 | 3.95 |
| Axonal Guidance Signaling | 1.01 | 0.64 | 3.42 |
| Actin Cytoskeleton Signaling | 0.00 | 0.91 | 3.05 |
| Caveolar-mediated Endocytosis Signaling | 0.00 | 0.48 | 3.17 |
| Cyclins and Cell Cycle Regulation | 0.74 | 0.00 | 2.90 |
| Cell Cycle: G1/S Checkpoint Regulation | 0.82 | 0.00 | 2.76 |
| Epoxysqualene Biosynthesis | 2.29 | 0.00 | 1.27 |
| Ketogenesis | 3.55 | 0.00 | 0.00 |
| Cellular Effects of Sildenafil (Viagra) | 0.00 | 1.44 | 2.06 |
| Zymosterol Biosynthesis | 1.82 | 1.48 | 0.00 |
| Integrin Signaling | 0.00 | 0.49 | 2.78 |
| Inhibition of Matrix Metalloproteases | 1.02 | 1.69 | 0.54 |
| ILK Signaling | 0.00 | 0.00 | 3.25 |

**Figure 3. Deregulation of Cellular Redox and Mitochondrial Target Genes in DMD BMI1$^{Over}$ Myoblasts and Controls**
(A) Venn diagram to identify common deregulated (DE) genes in control and DMD myoblasts overexpressing BMI1.
(B) Canonical pathway enrichment (reported as $-\log_{10}$ p value) for the 54 shared genes as compared with DMD and control-only DE genes.
(C) Validation of selected target genes in independent biological replicas (mean ± SD of three independent preparations; *p < 0.05 **p < 0.01; ***p < 0.001).
(D) Level of PRDX2 and its oxidized form, PRDX2-SO3, as assessed by western blot analysis.

ratio evaluation (Figures 4B and 4C). As OXPHOS and aerobic glycolysis represent the two main energy sources for cells, the prediction is that BMI1 overexpression has a significant impact on the energetic state of the cells (ATP levels). Bioenergetic profiling of DMD myoblasts overexpressing BMI1 showed increased oxygen consumption in basal condition with a concomitant increase in the calculated ATP levels, and a greater maximal respiration capacity (Figures 4D and 4E). Increased ATP production in DMD BMI1$^{Over}$ myoblasts as assessed by an ATP luciferase assay (Figure 4F) lends support to this conclusion. Importantly, increased ATP production and increased OCR levels were validated in non-immortalized DMD myoblasts upon overexpression of BMI1 (Figures S4C–S4F) with the opposite effect detected upon silencing of BMI1 (Figures S4C, S4D, S4E, and S4G).

To assess whether the effect on respiratory capacity was dependent on an increase in the efficiency of the mitochondria or on an increase in the number of organelles available in the cells, we measured the mtDNA copy number. We did not see an increase in mitochondria number (Figure S4B), in keeping with the observed effect being exerted by BMI1 on mitochondrial activity.

## BMI1 Overexpression Protects DMD Myoblasts from Oxidative Damage

Glutathione (GSH) is the main antioxidant of aerobic cells and prevents damage to important cellular components caused by ROS, such as free radicals and peroxides (Mari et al., 2009). Because we observed upregulation of genes involved in glutathione regulation (*PRDX2* and *GPX3*) in BMI1$^{Over}$ myoblasts, we set out to further analyze the

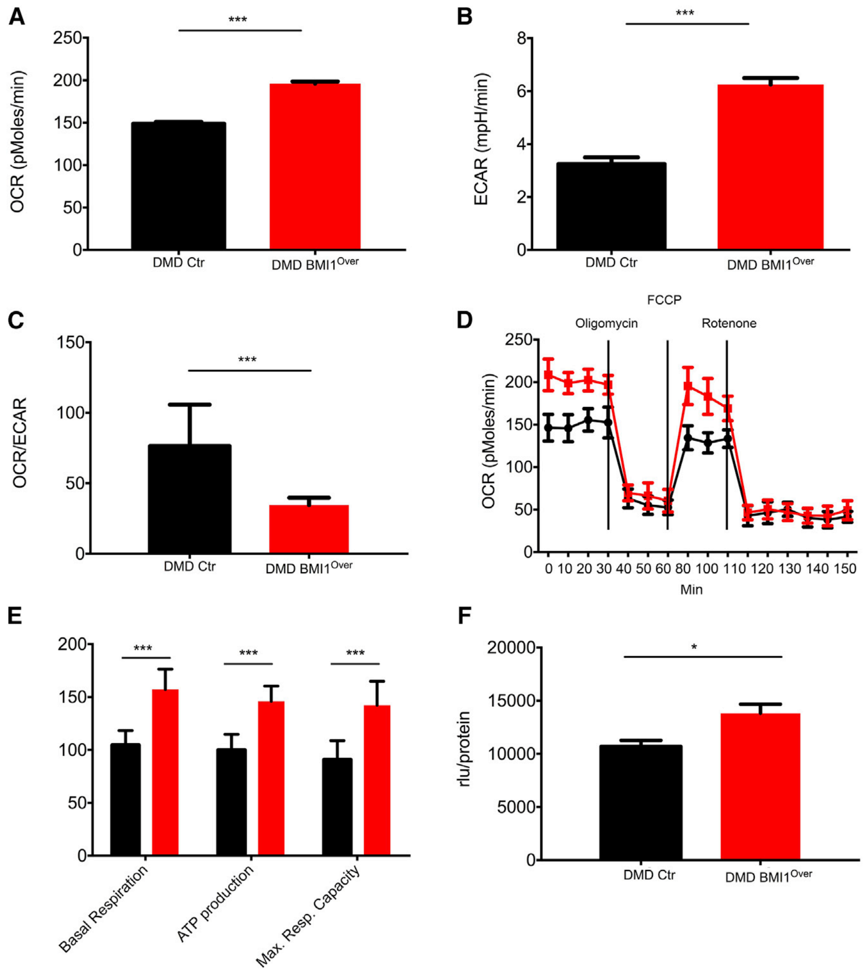


**Figure 4. BMI1 Overexpression Enhances Mitochondrial Respiration and ATP Production**

(A) Basal oxygen consumption rate (OCR, indicative of mitochondrial OXPHOS) was measured in DMD myoblasts overexpressing BMI1 (red) and compared with DMD myoblasts (black) with an XF24 Extracellular Analyzer (***$p < 0.001$).

(B and C) Measurement of basal extracellular acidification rate (ECAR, representing glycolysis) measured in DMD control myoblasts (black) and overexpressing BMI1 (red) (B), and OCR/ECAR ratios of DMD myoblasts control (black) and overexpressing BMI1 (red) (C) (***$p < 0.001$).

(D) DMD control myoblasts (black) and overexpressing BMI1 (red) were seeded in a Seahorse XF-24 analyzer and real-time OCR was determined during sequential treatments with oligomycin (ATP-synthase inhibitor), FCCP (mitochondria uncoupler), and rotenone (ETC inhibitors). Data are from three independent experiments in which each data point represents the mean of 12 replicates for each condition ± SD.

(E) Basal respiration rate, ATP production, and maximal respiration rate in DMD control myoblasts and overexpressing BMI1 (calculated from data shown in D; ***$p < 0.001$).

(F) ATP content measured with a luciferase assay and normalized against protein concentration in DMD control and DMD BMI1$^{Over}$ myoblasts (rlu/prot) (mean ± SD of three independent experiments; *$p < 0.05$).

GSH/GSSG ratio in DMD cultures, where a reduction in the GSH/GSSG ratio is considered indicative of oxidative stress (Owen and Butterfield, 2010). We observed an increased GSH/GSSG ratio in DMD BMI1$^{Over}$ myoblasts compared with control DMD cells (Figure 5A), indicating a reduced oxidative stress in DMD BMI1$^{Over}$ myoblasts.

Because PRDX2 is known to protect DNA from hydrogen peroxides and thermal stress damage (Lee et al., 2015), we set out to assess the degree of oxidative DNA damage in DMD myoblasts overexpressing BMI1 to determine whether its increase of expression could facilitate the cellular compliance with the higher energetic state induced by BMI1$^{Over}$ and sustain differentiation through protection from DNA damage.

To assess the response to induction of DNA damage in DMD BMI1$^{Over}$ myoblasts, we performed a 10-min challenge with 50 μM $H_2O_2$ and assessed DNA damage by γH2AX staining, a sensitive marker of double-strand DNA breaks (Mariotti et al., 2013). While control DMD cells showed a significant increase in the intensity and total area of γH2AX foci per nucleus, DMD BMI1$^{Over}$ myoblasts did not show this increase and both the intensity and the total area of γH2AX foci were unchanged compared with the non-treated cells (Figures 5B–5D), in keeping with DMD BMI1$^{Over}$ myoblasts being more resistant to DNA damage.

To validate the dependency on PRDX2 of the impact of BMI1 on oxidative stress protection, we treated DMD BMI1$^{Over}$ and controls with conoidin A, a well-characterized PRDX2 inhibitor. Measurement of the ATP level in DMD BMI1$^{Over}$ myoblasts upon $H_2O_2$ treatment showed that its increase is neutralized upon treatment with conoidin A (Figure 5E). These data are consistent with PRDX2 mediating, at least in part, the effect of BMI1$^{Over}$ on the energetic state of the cells.

## DMD BMI1$^{Over}$ Myoblasts Are Protected from DNA Damage upon Engrafting into a Dystrophic Mouse Model

To investigate whether modulation of BMI1 expression in human myoblasts could be exploited therapeutically to

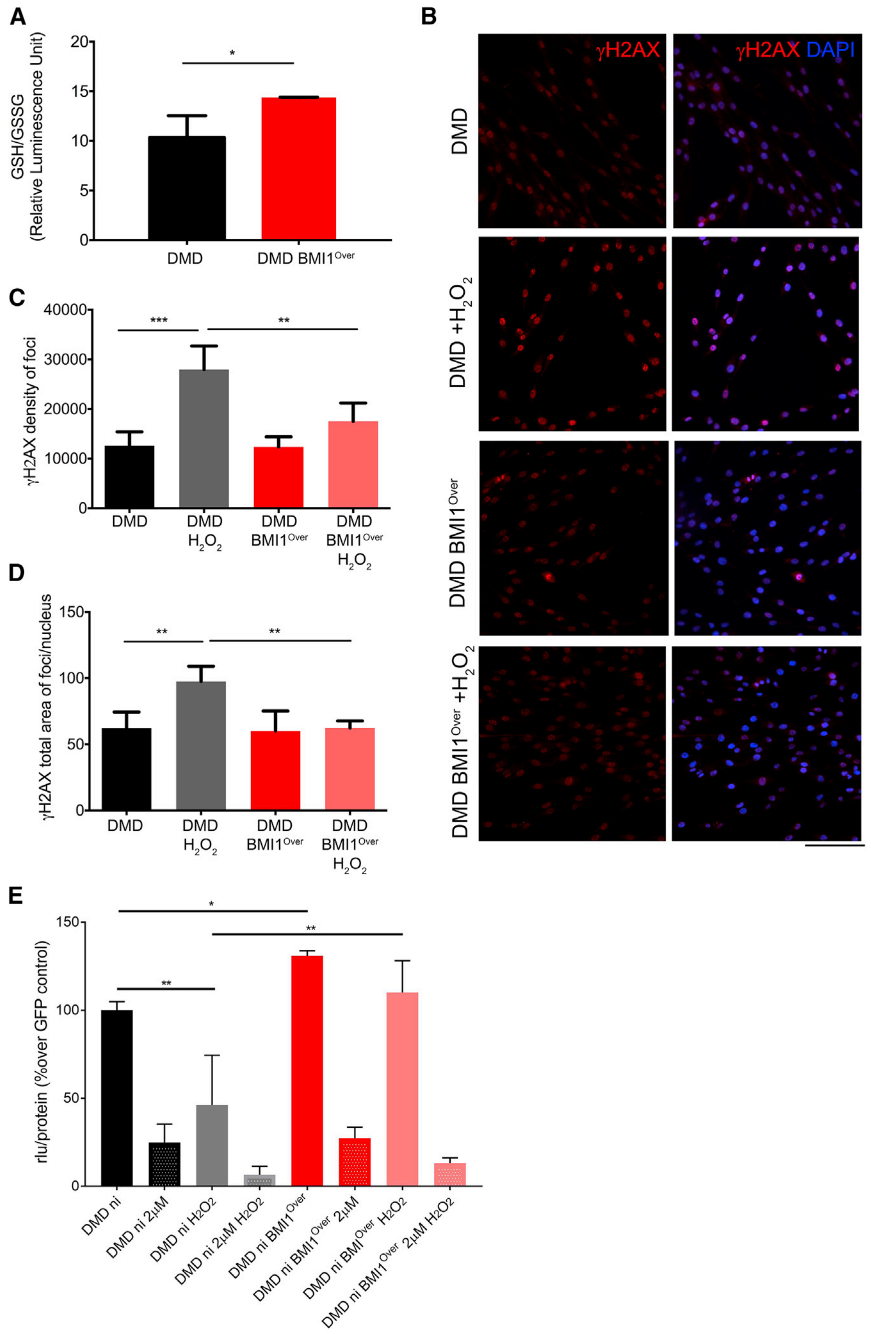


**Figure 5. Increase of Reduced Glutathione Form and Protection against DNA Damage upon BMI1 Overexpression**

(A) GSH/GSSG ratio expressed as relative luminescence unit (rlu) in DMD myoblasts overexpressing BMI1 compared with DMD control (mean ± SD of three independent experiments; $*p < 0.05$).

(B–D) Representative images (B) of γH2AX staining of DMD myoblasts overexpressing BMI1 and DMD controls either untreated or treated with 50 mM $H_2O_2$. Quantification of mean intensity (C) and mean area (D) of foci per nucleus (mean ± SD of three independent experiments; $**p < 0.01$, $***p < 0.001$). Quantification of the number of positive cells was carried out on at least 9 fields (20×) for each case using the InCell Developer Toolbox software (GE Healthcare). Scale bar, 250 μm.

(E) Effect of conoidin A (2 μM) treatment on ATP level, in normal condition or upon treatment with 50 μM $H_2O_2$ in DMD $BMI1^{Over}$ myoblasts compared with controls (mean ± SD of three independent experiments; $*p < 0.05$, $**p < 0.01$).

enhance the regenerative potential of the skeletal muscle in pathological conditions, we performed an engraftment of human myoblasts in a dystrophic mouse host. $10^6$ DMD $BMI1^{Over}$ myoblasts and DMD controls were injected intramuscularly into the tibialis anterior (TA, n = 6.) muscles of α-sarcoglycan null/scid/beige dystrophic mice (Tedesco et al., 2012). The number of donor-derived myofibers and their size were assessed in serial sections double stained for hLaminA/C and hSpectrin 25 days after transplantation. No significant difference was observed in terms of number or cross-sectional area (CSA) of donor-derived myofibers (Figures 6A–6C) upon BMI1 overexpression. Although we could detect rare $PAX7^+$ satellite cells of human origin, no increase was detected in muscles that had been transplanted with BMI1-overexpressing cells (data not shown). However, $BMI1^{Over}$ affected the energetic state of the transplanted muscles, as an increase (1.8-fold change) in ATP content was detected in TA muscles

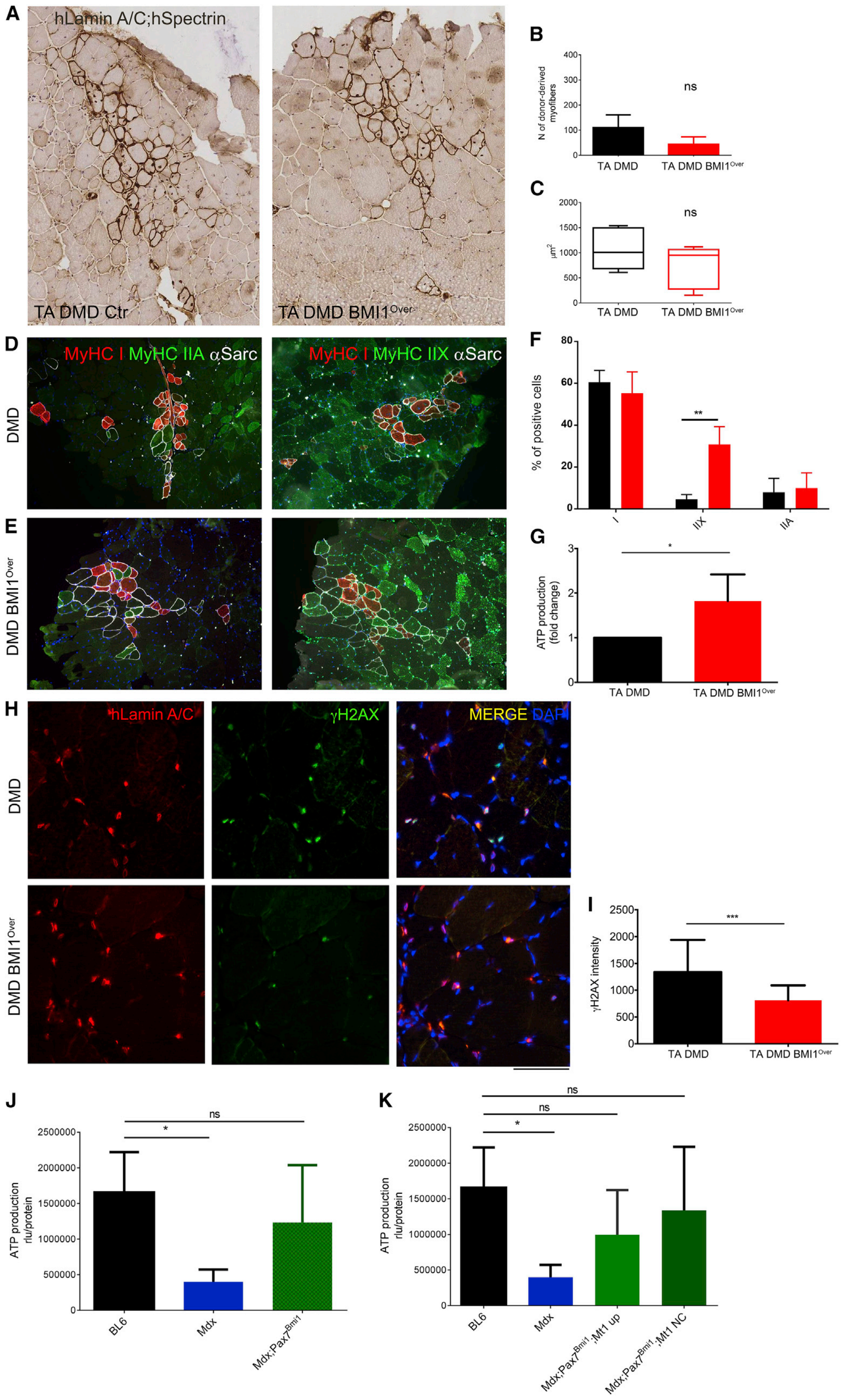


*(legend on next page)*

injected with BMI1$^{Over}$ cells compared with the controls (Figure 6D). Staining for MyHC I and MyHC IIA/MyHCIIX to identify slow and moderately fast/fast fiber types, respectively, show a higher number of MyHC IIX fast fibers among the fibers originating from the DMD BMI1$^{Over}$ myoblasts engrafted into the dystrophic TA muscles (Figures 6E–6G). Importantly, these fibers use ATP as a major storage fuel for their short-term anaerobic activity; thus, these data are in agreement with the increased ATP production as biochemically assessed (Figures 6D and 6G).

As a further parallel to the *in vitro* results, a reduction of DNA damage was observed in TA muscles engrafted with DMD BMI1$^{Over}$ myoblasts compared with controls in a double staining for γH2AX and hLaminA/C (Figures 6E and 6F).

Because the number of fibers of donor origin was relatively small and the repair was segmental, an assessment of muscle strength was not possible in the xenografted mice.

We have previously shown (Di Foggia et al., 2014) that Bmi1 overexpression in satellite cells leads to improved muscle strength in *Mdx* mice, resulting in a better performance on a treadmill compared with controls. Enhanced protection from oxidative stress was essential for the improved functional outcome and was observed only when Bmi1 overexpression triggered MT-1 upregulation. To validate the hypothesis that the increased ATP levels are an essential component of BMI1-mediated enhanced muscle strength, we measured ATP in *Mdx*, *Mdx*;*Pax7*$^{Bmi1}$, and control littermates. We show reduced ATP in *Mdx* muscles, although its levels are similar to the normal control in *Mdx* muscles overexpressing Bmi1 in Pax7$^+$ satellite cells (Figure 7A), in keeping with the interpretation that a higher energetic state is responsible for the better functional performance observed. Interestingly however, no difference in the ATP levels was observed in the group overexpressing MT1 compared with the group not overexpressing MT1 (Figure 7B), raising the possibility that the increased energetic state translates to a better functional performance only when enhanced protection from oxidative stress also occurs (Di Foggia et al., 2014). Our data show increased ATP levels and protection from oxidative stress in mice xenografted with BMI1$^{Over}$ myoblasts, raising the possibility that BMI1 overexpression may confer enhanced muscle strength also in this model.

## PcG Protein Expression Is Reduced in Quiescent and Committed Satellite Cells in Chronic Neuromuscular Diseases Independently of Their Etiology

To extend our findings to other human muscular conditions, we have characterized the expression of PcG proteins in a wide spectrum of neuromuscular disorders, including inflammatory myopathies and motor neuron disease (MND). To better understand the role of PcG in the regenerative process in human muscles, we also included biopsies from morphologically normal muscles with evidence of regeneration in the study. Cases that did not show histological abnormalities at the time of biopsy were considered normal comparators.

Immunofluorescence for BMI1, including co-localization with markers of quiescent and activated satellite cells, was performed on six cases with normal morphology, five biopsies with evidence of regeneration and a normal morphology, five cases of idiopathic inflammatory myopathy (IIM), and four cases of MND (Figure 7). A significant increase in the percentage of PAX7$^+$; MYF5$^-$ quiescent satellite cells was noted in cases with regeneration, with no changes seen in the other conditions (Figures 7A–7D and 7E). A significant depletion of Bmi1$^+$ committed satellite cells (PAX7$^-$; MYF5$^+$) was seen in MND cases while no changes were observed in all other conditions (Figures 7A–7D and 7F). No difference was noted in the percentage of BMI1$^+$ cells in PAX7$^+$; MYF5$^-$ and double-positive cells (data not shown).

Interestingly, higher BMI1 expression is seen in the PAX7$^+$ cell population in regenerating muscles, while lower BMI1 expression is seen in the PAX7$^+$ cell population in IIM cases (Figure 7G).

**Figure 6. BMI1 Overexpression Increases ATP and Reduces DNA Damage upon Transplantation of DMD BMI1$^{Over}$ Myoblasts in Sgca-null/scid/bg TA Muscle**

(A–I) Representative images of hLaminA/C and hSpectrin 3,3′-diaminobenzidine staining (A) on frozen transverse sections of Sgca-null/scid/bg TA injected with DMD Ctr or DMD$^{Over}$ human myoblasts and analyzed 25 days after injection. Quantification with Definiens software of the number of integrated fiber (B) and the mean CSA ($\mu m^2$) (C) (mean ± SD, n = 6 animals; ns, not significant). Representative images of MyHC I (red), MyHC IIA or MyHC IIX (green), and α-sarcoglycan (white) staining on frozen transverse sections of Sgca-null/scid/bg TA injected with DMD Ctr (D) or DMD$^{Over}$ (E) human myoblasts. Quantification of positive fibers for MyHC antigens expressed as percentage over total number of α-sarcoglycan-positive fibers (F) (mean ± SD, n = 6 animals; $^{**}p < 0.01$) and (G) ATP quantification on scraped frozen transverse sections ($^{*}p < 0.05$). Representative images (H) and quantification (I) of γH2AX and hLamin A/C staining on transverse sections of muscles injected with DMD Ctr or DMD$^{Over}$ human myoblasts (mean ± SD, n = 6 animals; $^{***}p < 0.001$).

(J and K) ATP quantification on transverse section of forelimb muscles isolated from BL6 (n = 4), Mdx (n = 6), Mdx; Pax7; Bmi1; Mt up (n = 4), and Mdx; Pax7; Bmi1; Mt NC (n = 7) expressed as relative luminescence unit normalized versus protein concentration ($^{*}p < 0.05$; ns, not significant).

The scale bar represents 500 μm in (A, D, and E) and 125 μm in (H).

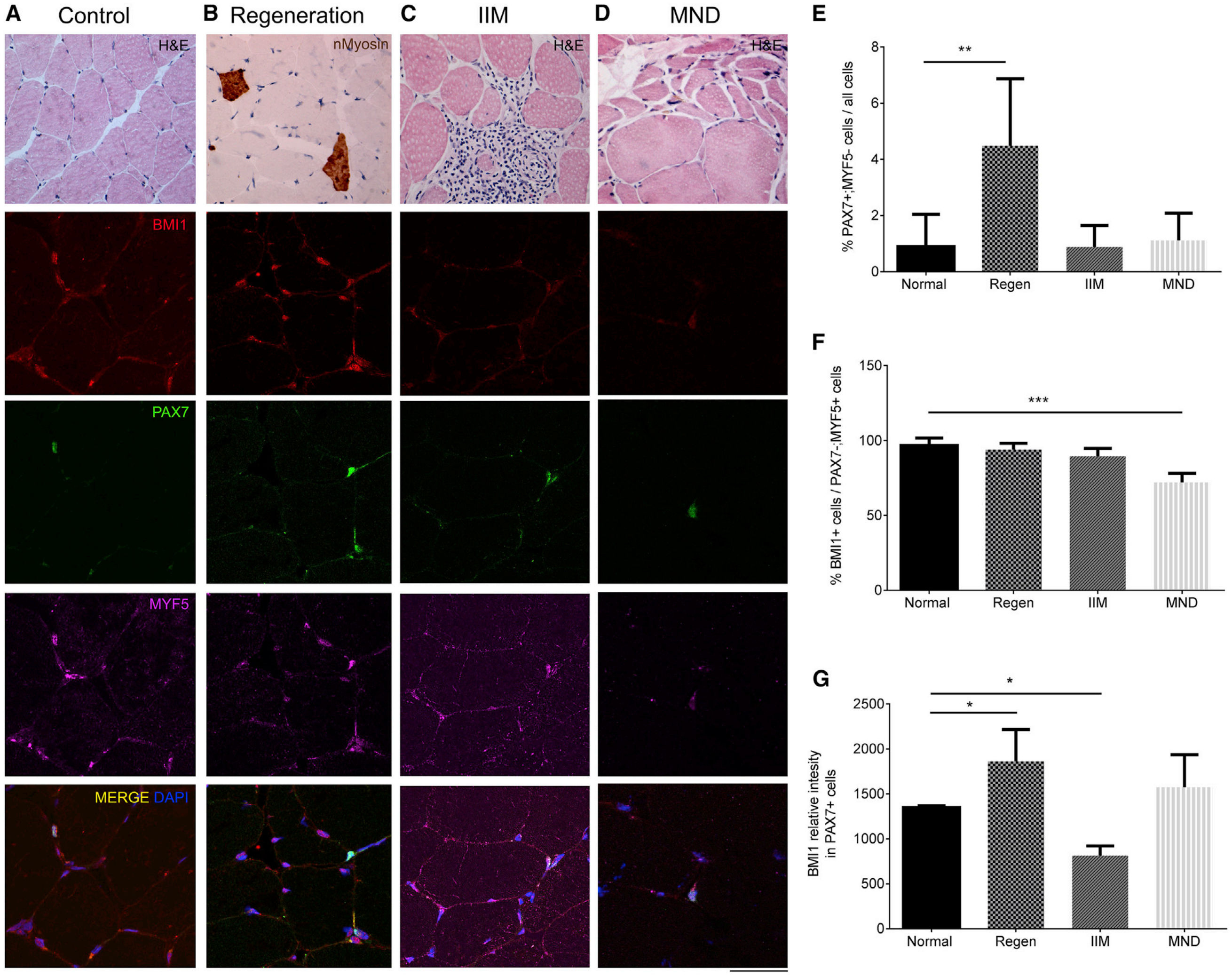


**Figure 7. Characterization of BMI1 Expression in Human Chronic Neuromuscular Disorders**

(A–D) Histological features (H&E) and representative images of a triple immunostaining for BMI1, PAX7, and MYF5 on frozen muscle transverse sections from morphologically normal muscle (n = 6) (A), normal biopsies with evidence of regeneration (n = 5) (B), idiopathic inflammatory myopathy (IIM) (n = 5) (C), and motor neuron disease (MND) (n = 4) (D). Scale bar, 125 μm.

(E) Quantification of the number of quiescent satellite cells ($PAX7^{+}$; $MYF5^{-}$) over the total number of nuclei (mean ± SD; $**p < 0.01$).

(F) Quantification of $BMI1^{+}$ cells among committed ($PAX7^{-}$; $MYF5^{+}$) satellite cells (mean ± SD; $***p < 0.001$).

(G) BMI1 intensity level in $PAX7^{+}$ cells (mean ± SD; $*p < 0.05$).

Quantification of the number of positive cells was carried out on at least 5 high-power fields (40×) for each case.

Triple immunolabeling for PAX7, BMI1, and EZH2 (Figure S5) revealed an overall decrease of $EZH2^{+}$ cells in IIM and MND cases (Figure S6A) with a significant decrease of $EZH2^{+}$ cells in $PAX7^{+}$ satellite cell (Figure S6B). A decrease of $BMI1^{+}$ and $EZH2^{+}$ cells was observed in MND cases (Figure S6C).

In line with what was observed for BMI1, higher EZH2 expression was also observed in $PAX7^{+}$ cells (Figure 6F).

Immunostaining for EZH1 was also performed on the same selection of cases, revealing a decrease in $EZH1^{+}$ cells in IIM and MND (Figure S6D).

We show here that the expression of PcG proteins is decreased in both quiescent and committed satellite cells of chronic neuromuscular diseases, independently of their defining etiology.

## DISCUSSION

We demonstrate here that BMI1 overexpression in human myoblasts increases the mitochondrial activity and the aerobic glycolysis reaction leading to an enhanced energetic

state with increased ATP production, and ameliorates the myogenic differentiation capacity of the myoblasts. The concomitant protection of the cells from DNA damage both *in vitro* and *in vivo* upon xenografting in a dystrophic mouse model lends support to the notion that a combination of increased energetic state and protection from oxidative stress is essential for a functional impact of BMI1 overexpression on muscle homeostasis in dystrophic mouse models. These data show that our observation in genetically engineered mouse models (Di Foggia et al., 2014) is translatable to human cells.

The role of PcG proteins during muscle regeneration was recently studied in mouse models. BMI1 expression is upregulated in satellite cells during the first phases of muscle regeneration, and BMI1 ablation leads to an increased proportion of committed myogenic precursor cells (Di Foggia et al., 2014). In keeping with a depletion of the satellite cell pool, when the muscle is challenged with repeated injuries, $BMI1^{-/-}$ mice display a compromised and delayed regeneration with defects in the maturation of the regenerating fibers (Robson et al., 2011). The phenotype observed in $BMI1^{-/-}$ mice is similar to that observed when EZH2 is ablated specifically in the satellite cells (Juan et al., 2011; Woodhouse et al., 2013). The role of both EZH2 and BMI1 has been investigated also in the murine dystrophic environment. Tumor necrosis factor-induced downregulation of Notch levels, a key molecular mechanism in muscle regeneration, is mediated via recruitment of EZH2 on a specific region of the *Notch1* gene, with subsequent increased trimethylation of K27, both *in vitro* and *in vivo* (Bhattacharyya et al., 2009; Palacios et al., 2010). In the *Mdx* mouse, BMI1 is downregulated in the satellite cells (Di Foggia et al., 2014) and *Mdx*;$BMI1^{-/-}$ show fewer and smaller centrally nucleated fibers in the diaphragm (Robson et al., 2011), a muscle known to be particularly affected by chronic muscle wasting and repeated cycles of repair due to its essential role in respiration (Robson et al., 2011). In genetically engineered mouse models, BMI1 overexpression in the satellite cells increases the $Pax7^{+}$/$MyoD^{-}$ proportion of the satellite cells *in vitro*, without impairing their differentiation potential (Di Foggia et al., 2014). Mild overexpression of BMI1 specifically in the satellite cells enhances the muscle strength of *Mdx* mice after challenge with treadmill exercise for 6 weeks (Di Foggia et al., 2014).

Here, we show a reduced number of cells expressing BMI1in both quiescent and committed DMD myoblasts with its expression level also reduced in these cells. These findings raise the possibility that BMI1 depletion contributes to the impaired regenerative process also in the human dystrophic context. We show here that upregulation of BMI1 expression in human DMD myoblasts ameliorates their differentiation potential, an effect even more pronounced than in normal myoblasts. While BMI1 overexpression increases the proliferation of the normal myoblasts, this effect is not noted in DMD myoblasts, where a decrease in proliferation is observed instead. As the basal proliferation rate of the DMD cultures was significantly lower in comparison with normal cells, it is possible that their proliferation capacity is already on a plateau because of cellular exhaustion and cannot be further increased. However, this did not impact on the enhanced differentiation induced by BMI1 overexpression in human DMD myoblasts.

The connection between mitochondrial processes and stem cell function has been recently highlighted (Garcia-Prat et al., 2016). Bmi1 plays a well-defined role in maintaining mitochondrial function and redox homeostasis in various stem cell populations, hence linking regulation of cellular metabolism with stem and progenitor cell function (Liu et al., 2009). We show here an enhanced energetic state with increased ATP production upon overexpression of BMI1 in human DMD myoblasts both *in vitro* and *in vivo* upon transplantation of these cells into a dystrophic environment. *Sgca-null/scid/bg* mice (Tedesco et al., 2012) were chosen as recipient as this is a more severe dystrophic model compared with the *Mdx* mouse with significant fibrosis, and would therefore provide a more challenging environment for the transplanted DMD myoblasts. Interestingly, the morphological substrate for the increased ATP production was shown to be the higher number of MyHC IIX fast fibers originating from the DMD $BMI1^{Over}$ myoblasts engrafted into the dystrophic TA muscles.

Increased ATP levels were also observed in *Mdx* mice where Bmi1 is overexpressed in $Pax7^{+}$ satellite cells, similarly to that observed in the xenograft model. Importantly, despite the enhanced energetic state, a significant functional impact with amelioration of the performance of the dystrophic muscles under a forced exercise regime was found only when concomitant reduction of DNA damage occurred, an event which was dependent on metallothionein 1-mediated protection from oxidative stress-induced cellular damage.

We confirmed upregulation of *MT1* in human DMD myoblasts upon BMI1 overexpression in a genome-wide expression analysis. Interestingly, we also found upregulation of *PRDX2* in these cells. PRDX2 belongs to the peroxiredoxin family, which are thioredoxin-family antioxidant enzymes that scavenge cellular peroxides and contribute to redox homeostasis (Rhee and Woo, 2011; Winterbourn and Hampton, 2015). Functionally we observed an enhanced scavenging role and increased protection from DNA damage in DMD $BMI1^{Over}$ myoblasts compared with control DMD cells, as assessed by increased GSH/GSSG ratio and a significantly lower intensity of γH2AX foci, respectively, both *in vitro* and *in vivo* in xenografts. Importantly, we show that PRDX2 mediates at least in part the effect of

BMI1$^{Over}$ on the energetic state of the cells, as treatments with PRDX2 inhibitors neutralized the increased ATP production induced by BMI1$^{Over}$ in DMD myoblasts. These data are well in keeping with our previous observation in genetically engineered mouse models (Di Foggia et al., 2014).

In conclusion, our preclinical data in mouse models support the development of pharmacological approaches to target BMI1-mediated mitochondrial regulation and protection from DNA damage as a novel therapeutic approach to stimulate endogenous, but possibly also transplanted, satellite cell self-renewal to sustain and enhance their contribution to muscle regeneration in DMD patients. Importantly, we show that BMI1 expression is reduced in conditions of chronic muscle wasting independently of their etiology, hence raising the possibility that the patient benefit of such an approach may be wider than originally predicted.

## EXPERIMENTAL PROCEDURES

### Human Biopsies

Surplus material of muscle biopsies performed at Barts Health NHS in the context of the clinical evaluation of patients with neuromuscular disorders was used. Biopsies of DMD patients and age-matched controls were obtained from the MRC Center for Neuromuscular Diseases Biobank London (REC 06/Q0406/33) (R. Phadke and F. Muntoni). The use of human muscle samples was in agreement with the UK Human Tissue Act 2004, and its use for this specific research had ethical approval (East London and The City REC Alpha, ReDA Reference: 006244). All patients or their legal guardians gave written informed consent.

### Human Myoblast Cultures

Immortalized and non-immortalized human myoblasts (isolated from DMD and histologically normal muscle biopsies, Table S1) were obtained from the MRC Center for Neuromuscular Diseases Biobank (Mamchaoui et al., 2011) and used in this study under the same ethics stated above. Cells were maintained in Skeletal Muscle Medium plus supplements (Promocell Kit, C-23060), 10% fetal bovine serum (PAA A15-151), 1× GlutaMAX (Invitrogen 35050038), and 5 μg/mL gentamicin (Sigma G1272). Differentiation was induced at confluence by replacing the growth medium with DMEM and 2% horse serum.

### Cell Transplantation Experiments

DMD human myoblasts were infected with BMI1$^{Over}$ and GFP lentiviral particles. Cells ($1 \times 10^6$) were transplanted into TA muscles (n = 6) of Sgca-null/scid/beige mice (Tedesco et al., 2012) 7 days later. After 25 days the muscles were collected, frozen in isopenthane, and mounted on Tragacanth gum (Sigma). Serial sections (8 μm thick) were cut, stained for specific antigens, and analyzed with ImageJ or Definiens AG (Developer XD, Munich). This work was conducted under UK Home Office Project Licenses no. 70/7435 and 70/8566.

### Statistical Analysis

Unless specified in the text, all the graphs represent population mean ± SD. Statistical analysis was performed with Prism statistical analysis software (GraphPad), Student's t test for single comparisons and ANOVA for multiple comparisons were applied. Significance is indicated in figures as ***$p < 0.001$, **$p < 0.01$, and *$p < 0.05$.

## SUPPLEMENTAL INFORMATION

Supplemental Information includes Supplemental Experimental Procedures, six figures, and one table and can be found with this article online at http://dx.doi.org/10.1016/j.stemcr.2017.06.009.

## AUTHOR CONTRIBUTIONS

S.D., P.K., M.N.-C., M.R., and V.d.F. conducted the wet lab experiments; S.D., L.G.R., P.K., M.N.-C., F.S.T., M.R., V.d.F., and S.M. analyzed the data; C.P.C. and M.R.B. conducted and analyzed the computational experiments; M.E. performed the image analysis; S.D., M.N.-C., F.S.T., and S.M. designed the experiments; A.R. contributed patient samples and clinical data; S.M. wrote the paper with contributions from all authors.


## ACKNOWLEDGMENTS

We are grateful to Jennifer Morgan, UCL Institute of Child Health, for critically reading the manuscript. This work is funded by grants from Barts Charity (468/1610) to A.R. and S.M. and the Muscular Dystrophy UK (RA4/788/4) and the Medical Research Council UK (G0802546/1) to S.M. and L.G.R. F.S.T. is funded by the European Union's 7th Framework project PluriMes (grant no. 602423), Muscular Dystrophy UK, Fundació la Marató de TV3, and the UK National Institute for Health Research (NIHR).

Received: November 14, 2016
Revised: June 17, 2017
Accepted: June 17, 2017
Published: July 20, 2017


## REFERENCES


Asp, P., Blum, R., Vethantham, V., Parisi, F., Micsinai, M., Cheng, J., Bowman, C., Kluger, Y., and Dynlacht, B.D. (2011). Genome-wide remodeling of the epigenetic landscape during myogenic differentiation. Proc. Natl. Acad. Sci. USA *108*, E149–E158.

Benedetti, S., Hoshiya, H., and Tedesco, F.S. (2013). Repair or replace? Exploiting novel gene and cell therapy strategies for muscular dystrophies. FEBS J. *280*, 4263–4280.

Bhattacharyya, J., Mihara, K., Yasunaga, S., Tanaka, H., Hoshi, M., Takihara, Y., and Kimura, A. (2009). BMI-1 expression is enhanced through transcriptional and posttranscriptional regulation during the progression of chronic myeloid leukemia. Ann. Hematol. *88*, 333–340.

Blau, H.M., Webster, C., and Pavlath, G.K. (1983). Defective myoblasts identified in Duchenne muscular dystrophy. Proc. Natl. Acad. Sci. USA *80*, 4856–4860.

Blau, H.M., Webster, C., Pavlath, G.K., and Chiu, C.P. (1985). Evidence for defective myoblasts in Duchenne muscular dystrophy. Adv. Exp. Med. Biol. *182*, 85–110.

Brack, A.S., and Rando, T.A. (2012). Tissue-specific stem cells: lessons from the skeletal muscle satellite cell. Cell Stem Cell *10*, 504–514.

Chal, J., Oginuma, M., Al Tanoury, Z., Gobert, B., Sumara, O., Hick, A., Bousson, F., Zidouni, Y., Mursch, C., Moncuquet, P., et al. (2015). Differentiation of pluripotent stem cells to muscle fiber to model Duchenne muscular dystrophy. Nat. Biotechnol. *33*, 962–969.

Chang, N.C., Chevalier, F.P., and Rudnicki, M.A. (2016). Satellite cells in muscular dystrophy - lost in polarity. Trends Mol. Med. *22*, 479–496.

Chen, Y., Li, L., Ni, W., Zhang, Y., Sun, S., Miao, D., Chai, R., and Li, H. (2015). Bmi1 regulates auditory hair cell survival by maintaining redox balance. Cell Death Dis. *6*, e1605.

Collins, C.A., Olsen, I., Zammit, P.S., Heslop, L., Petrie, A., Partridge, T.A., and Morgan, J.E. (2005). Stem cell function, self-renewal, and behavioral heterogeneity of cells from the adult muscle satellite cell niche. Cell *122*, 289–301.

Di Foggia, V., Zhang, X., Licastro, D., Gerli, M.F., Phadke, R., Muntoni, F., Mourikis, P., Tajbakhsh, S., Ellis, M., Greaves, L.C., et al. (2014). Bmi1 enhances skeletal muscle regeneration through MT1-mediated oxidative stress protection in a mouse model of dystrophinopathy. J. Exp. Med. *211*, 2617–2633.

Disatnik, M.H., Chamberlain, J.S., and Rando, T.A. (2000). Dystrophin mutations predict cellular susceptibility to oxidative stress. Muscle Nerve *23*, 784–792.

Dumont, N.A., Wang, Y.X., von Maltzahn, J., Pasut, A., Bentzinger, C.F., Brun, C.E., and Rudnicki, M.A. (2015). Dystrophin expression in muscle stem cells regulates their polarity and asymmetric division. Nat. Med. *21*, 1455–1463.

Endesfelder, S., Krahn, A., Kreuzer, K.A., Lass, U., Schmidt, C.A., Jahrmarkt, C., von Moers, A., and Speer, A. (2000). Elevated p21 mRNA level in skeletal muscle of DMD patients and mdx mice indicates either an exhausted satellite cell pool or a higher p21 expression in dystrophin-deficient cells per se. J. Mol. Med. (Berl.) *78*, 569–574.

Facchino, S., Abdouh, M., Chatoo, W., and Bernier, G. (2010). BMI1 confers radioresistance to normal and cancerous neural stem cells through recruitment of the DNA damage response machinery. J. Neurosci. *30*, 10096–10111.

Faist, V., Koenig, J., Hoeger, H., and Elmadfa, I. (1998). Mitochondrial oxygen consumption, lipid peroxidation and antioxidant enzyme systems in skeletal muscle of senile dystrophic mice. Pflugers Arch. *437*, 168–171.

Faist, V., Konig, J., Hoger, H., and Elmadfa, I. (2001). Decreased mitochondrial oxygen consumption and antioxidant enzyme activities in skeletal muscle of dystrophic mice after low-intensity exercise. Ann. Nutr. Metab. *45*, 58–66.

Fulle, S., Di, D.S., Puglielli, C., Pietrangelo, T., Beccafico, S., Bellomo, R., Protasi, F., and Fano, G. (2005). Age-dependent imbalance of the antioxidative system in human satellite cells. Exp. Gerontol. *40*, 189–197.

Garcia-Prat, L., Martinez-Vicente, M., Perdiguero, E., Ortet, L., Rodriguez-Ubreva, J., Rebollo, E., Ruiz-Bonilla, V., Gutarra, S., Ballestar, E., Serrano, A.L., et al. (2016). Autophagy maintains stemness by preventing senescence. Nature *529*, 37–42.

Ginjala, V., Nacerddine, K., Kulkarni, A., Oza, J., Hill, S.J., Yao, M., Citterio, E., van, L.M., and Ganesan, S. (2011). BMI1 is recruited to DNA breaks and contributes to DNA damage-induced H2A ubiquitination and repair. Mol. Cell. Biol. *31*, 1972–1982.

Gnocchi, V.F., Ellis, J.A., and Zammit, P.S. (2008). Does satellite cell dysfunction contribute to disease progression in Emery-Dreifuss muscular dystrophy? Biochem. Soc. Trans. *36*, 1344–1349.

Hill, M., Wernig, A., and Goldspink, G. (2003). Muscle satellite (stem) cell activation during local tissue injury and repair. J. Anat. *203*, 89–99.

Ismail, I.H., Andrin, C., McDonald, D., and Hendzel, M.J. (2010). BMI1-mediated histone ubiquitylation promotes DNA double-strand break repair. J. Cell Biol. *191*, 45–60.

Jin, J., Lv, X., Chen, L., Zhang, W., Li, J., Wang, Q., Wang, R., Lu, X., and Miao, D. (2014). Bmi-1 plays a critical role in protection from renal tubulointerstitial injury by maintaining redox balance. Aging Cell *13*, 797–809.

Juan, A.H., Derfoul, A., Feng, X., Ryall, J.G., Dell'Orso, S., Pasut, A., Zare, H., Simone, J.M., Rudnicki, M.A., and Sartorelli, V. (2011). Polycomb EZH2 controls self-renewal and safeguards the transcriptional identity of skeletal muscle stem cells. Genes Dev. *25*, 789–794.

Kuznetsov, A.V., Winkler, K., Wiedemann, F.R., von Bossanyi, P., Dietzmann, K., and Kunz, W.S. (1998). Impaired mitochondrial oxidative phosphorylation in skeletal muscle of the dystrophin-deficient mdx mouse. Mol. Cell. Biochem. *183*, 87–96.

Lee, S., Jia, B., Liu, J., Pham, B.P., Kwak, J.M., Xuan, Y.H., and Cheong, G.W. (2015). A 1-Cys peroxiredoxin from a thermophilic archaeon moonlights as a molecular chaperone to protect protein and DNA against stress-induced damage. PLoS One *10*, e0125325.

Liu, J., Cao, L., Chen, J., Song, S., Lee, I.H., Quijano, C., Liu, H., Keyvanfar, K., Chen, H., Cao, L.Y., et al. (2009). Bmi1 regulates mitochondrial function and the DNA damage response pathway. Nature *459*, 387–392.

Loperfido, M., Steele-Stallard, H.B., Tedesco, F.S., and VandenDriessche, T. (2015). Pluripotent stem cells for gene therapy of degenerative muscle diseases. Curr. Gene Ther. *15*, 364–380.

Maffioletti, S.M., Gerli, M.F., Ragazzi, M., Dastidar, S., Benedetti, S., Loperfido, M., VandenDriessche, T., Chuah, M.K., and Tedesco, F.S. (2015). Efficient derivation and inducible differentiation of expandable skeletal myogenic cells from human ES and patient-specific iPS cells. Nat. Protoc. *10*, 941–958.

Mamchaoui, K., Trollet, C., Bigot, A., Negroni, E., Chaouch, S., Wolff, A., Kandalla, P.K., Marie, S., Di Santo, J., St Guily, J.L., et al. (2011). Immortalized pathological human myoblasts: towards a universal tool for the study of neuromuscular disorders. Skelet Muscle *1*, 34.

Mari, M., Morales, A., Colell, A., Garcia-Ruiz, C., and Fernandez-Checa, J.C. (2009). Mitochondrial glutathione, a key survival antioxidant. Antioxid. Redox Signal. *11*, 2685–2700.

Marino, S., and Di Foggia, V. (2016). Invited review: polycomb group genes in the regeneration of the healthy and pathological skeletal muscle. Neuropathol. Appl. Neurobiol. *42*, 407–422.

Mariotti, L.G., Pirovano, G., Savage, K.I., Ghita, M., Ottolenghi, A., Prise, K.M., and Schettino, G. (2013). Use of the gamma-H2AX assay to investigate DNA repair dynamics following multiple radiation exposures. PLoS One *8*, e79541.

Mouly, V., Aamiri, A., Bigot, A., Cooper, R.N., Di Donna, S., Furling, D., Gidaro, T., Jacquemin, V., Mamchaoui, K., Negroni, E., et al. (2005). The mitotic clock in skeletal muscle regeneration, disease and cell mediated gene therapy. Acta Physiol. Scand. *184*, 3–15.

Onopiuk, M., Brutkowski, W., Wierzbicka, K., Wojciechowska, S., Szczepanowska, J., Fronk, J., Lochmuller, H., Gorecki, D.C., and Zablocki, K. (2009). Mutation in dystrophin-encoding gene affects energy metabolism in mouse myoblasts. Biochem. Biophys. Res. Commun. *386*, 463–466.

Owen, J.B., and Butterfield, D.A. (2010). Measurement of oxidized/reduced glutathione ratio. Methods Mol. Biol. *648*, 269–277.

Paasuke, R., Eimre, M., Piirsoo, A., Peet, N., Laada, L., Kadaja, L., Roosimaa, M., Paasuke, M., Martson, A., Seppet, E., et al. (2016). Proliferation of human primary myoblasts is associated with altered energy metabolism in dependence on ageing in vivo and in vitro. Oxid. Med. Cell. Longev. *2016*, 8296150.

Palacios, D., Mozzetta, C., Consalvi, S., Caretti, G., Saccone, V., Proserpio, V., Marquez, V.E., Valente, S., Mai, A., Forcales, S.V., et al. (2010). TNF/p38alpha/polycomb signaling to Pax7 locus in satellite cells links inflammation to the epigenetic control of muscle regeneration. Cell Stem Cell *7*, 455–469.

Pan, M.R., Peng, G., Hung, W.C., and Lin, S.Y. (2011). Monoubiquitination of H2AX protein regulates DNA damage response signaling. J. Biol. Chem. *286*, 28599–28607.

Percival, J.M., Siegel, M.P., Knowels, G., and Marcinek, D.J. (2013). Defects in mitochondrial localization and ATP synthesis in the mdx mouse model of Duchenne muscular dystrophy are not alleviated by PDE5 inhibition. Hum. Mol. Genet. *22*, 153–167.

Rando, T.A. (2001). The dystrophin-glycoprotein complex, cellular signaling, and the regulation of cell survival in the muscular dystrophies. Muscle Nerve *24*, 1575–1594.

Rhee, S.G., and Woo, H.A. (2011). Multiple functions of peroxiredoxins: peroxidases, sensors and regulators of the intracellular messenger H(2)O(2), and protein chaperones. Antioxid. Redox Signal. *15*, 781–794.

Robson, L.G., Di Foggia, V., Radunovic, A., Bird, K., Zhang, X., and Marino, S. (2011). Bmi1 is expressed in postnatal myogenic satellite cells, controls their maintenance and plays an essential role in repeated muscle regeneration. PLoS One *6*, e27116.

Rybalka, E., Timpani, C.A., Cooke, M.B., Williams, A.D., and Hayes, A. (2014). Defects in mitochondrial ATP synthesis in dystrophin-deficient mdx skeletal muscles may be caused by complex I insufficiency. PLoS One *9*, e115763.

Sambasivan, R., Yao, R., Kissenpfennig, A., Van, W.L., Paldi, A., Gayraud-Morel, B., Guenou, H., Malissen, B., Tajbakhsh, S., and Galy, A. (2011). Pax7-expressing satellite cells are indispensable for adult skeletal muscle regeneration. Development *138*, 3647–3656.

Tedesco, F.S., Gerli, M.F., Perani, L., Benedetti, S., Ungaro, F., Cassano, M., Antonini, S., Tagliafico, E., Artusi, V., Longa, E., et al. (2012). Transplantation of genetically corrected human iPSC-derived progenitors in mice with limb-girdle muscular dystrophy. Sci. Transl. Med. *4*, 140ra189.

Tidball, J.G., and Wehling-Henricks, M. (2007). The role of free radicals in the pathophysiology of muscular dystrophy. J. Appl. Physiol. *102*, 1677–1686.

Wang, Y.X., and Rudnicki, M.A. (2012). Satellite cells, the engines of muscle repair. Nat. Rev. Mol. Cell Biol. *13*, 127–133.

Whitehead, N.P., Yeung, E.W., and Allen, D.G. (2006). Muscle damage in mdx (dystrophic) mice: role of calcium and reactive oxygen species. Clin. Exp. Pharmacol. Physiol. *33*, 657–662.

Whitehead, N.P., Pham, C., Gervasio, O.L., and Allen, D.G. (2008). N-Acetylcysteine ameliorates skeletal muscle pathophysiology in mdx mice. J. Physiol. *586*, 2003–2014.

Winterbourn, C.C., and Hampton, M.B. (2015). Redox biology: signaling via a peroxiredoxin sensor. Nat. Chem. Biol. *11*, 5–6.

Woodhouse, S., Pugazhendhi, D., Brien, P., and Pell, J.M. (2013). Ezh2 maintains a key phase of muscle satellite cell expansion but does not regulate terminal differentiation. J. Cell Sci. *126*, 565–579.

Yin, H., Price, F., and Rudnicki, M.A. (2013). Satellite cells and the muscle stem cell niche. Physiol. Rev. *93*, 23–67.

Yoon, S.H., Sugamori, K.S., Grynpas, M.D., and Mitchell, J. (2016). Positive effects of bisphosphonates on bone and muscle in a mouse model of Duchenne muscular dystrophy. Neuromuscul. Disord. *26*, 73–84.

Stem Cell Reports, Volume *9*

## Supplemental Information

# Enhanced Energetic State and Protection from Oxidative Stress in Human Myoblasts Overexpressing BMI1

**Silvia Dibenedetto, Maria Niklison-Chirou, Claudia P. Cabrera, Matthew Ellis, Lesley G. Robson, Paul Knopp, Francesco Saverio Tedesco, Martina Ragazzi, Valentina Di Foggia, Michael R. Barnes, Aleksandar Radunovic, and Silvia Marino**

Dibenedetto et al. Figure S1

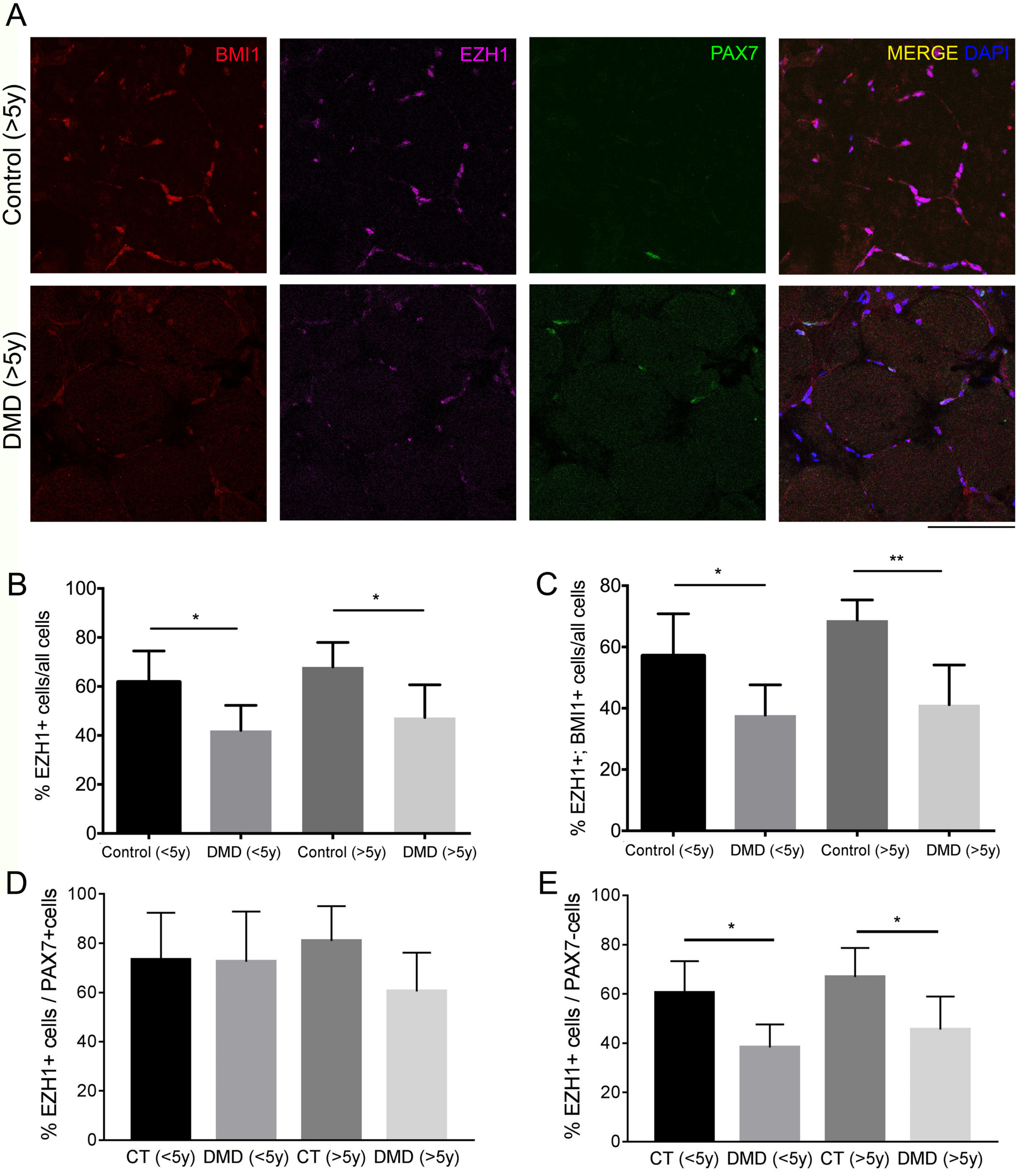

Dibenedetto et al. Figure S2

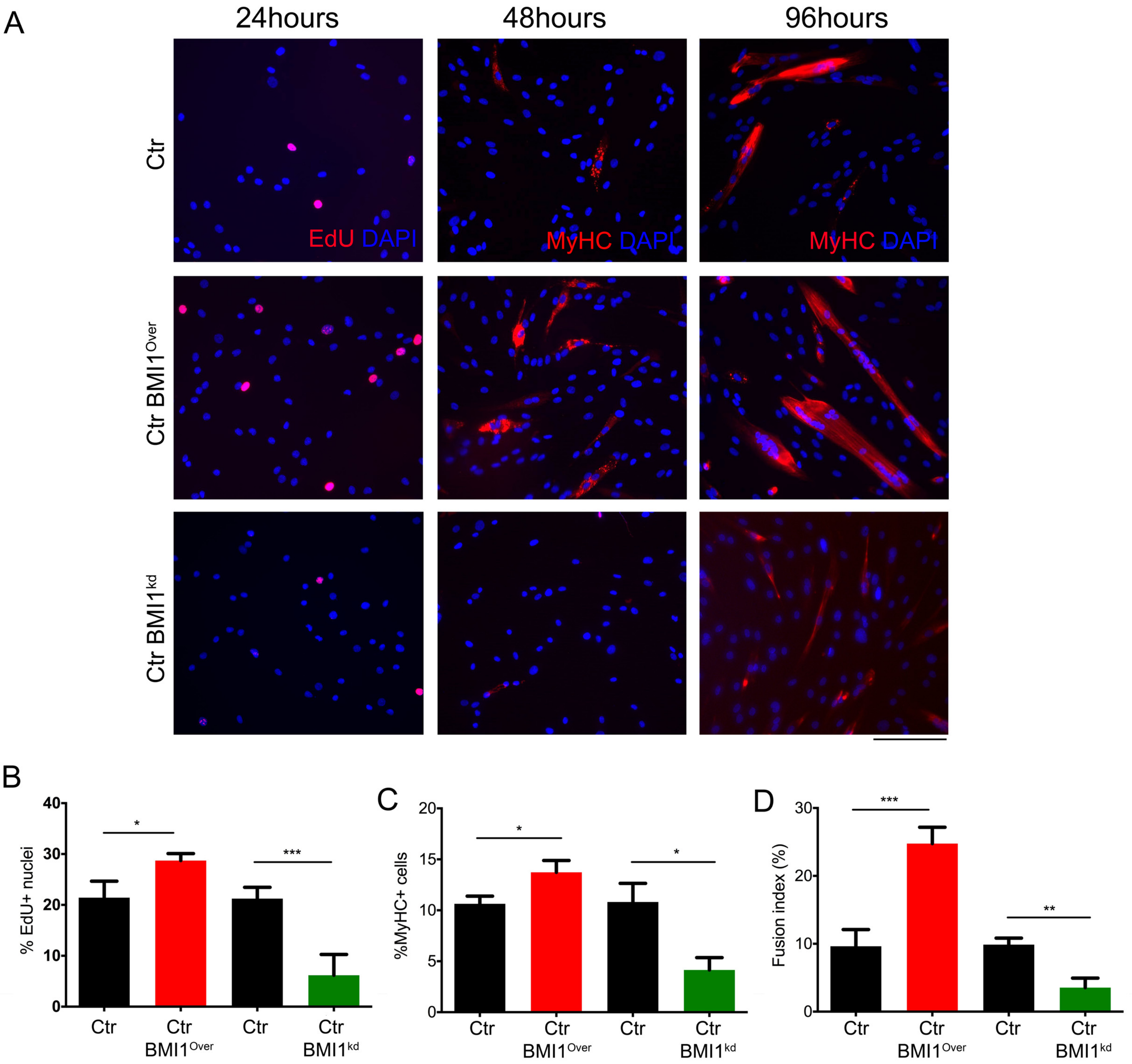

Dibenedetto et al. Figure S3

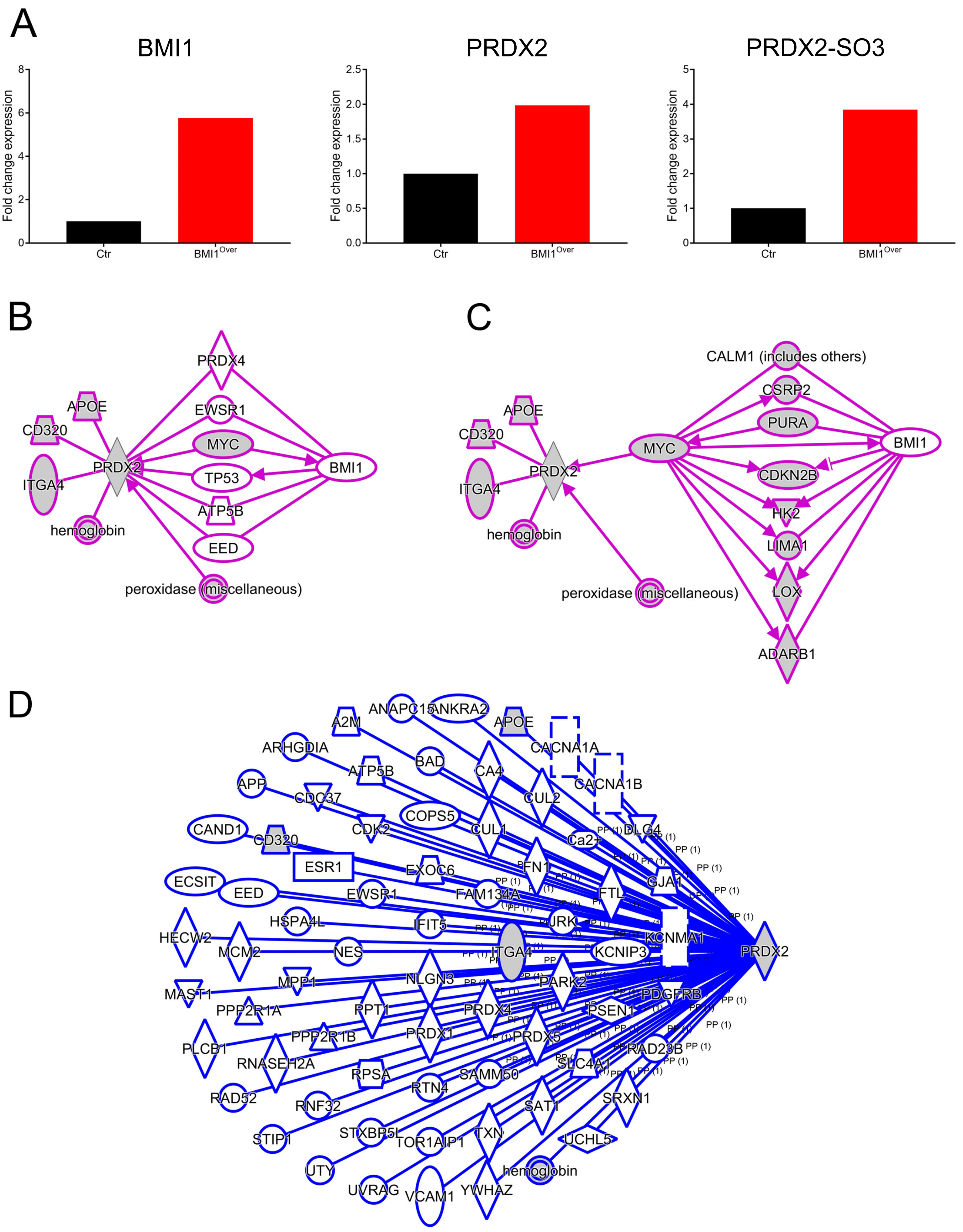

Dibenedetto et al. Figure S4

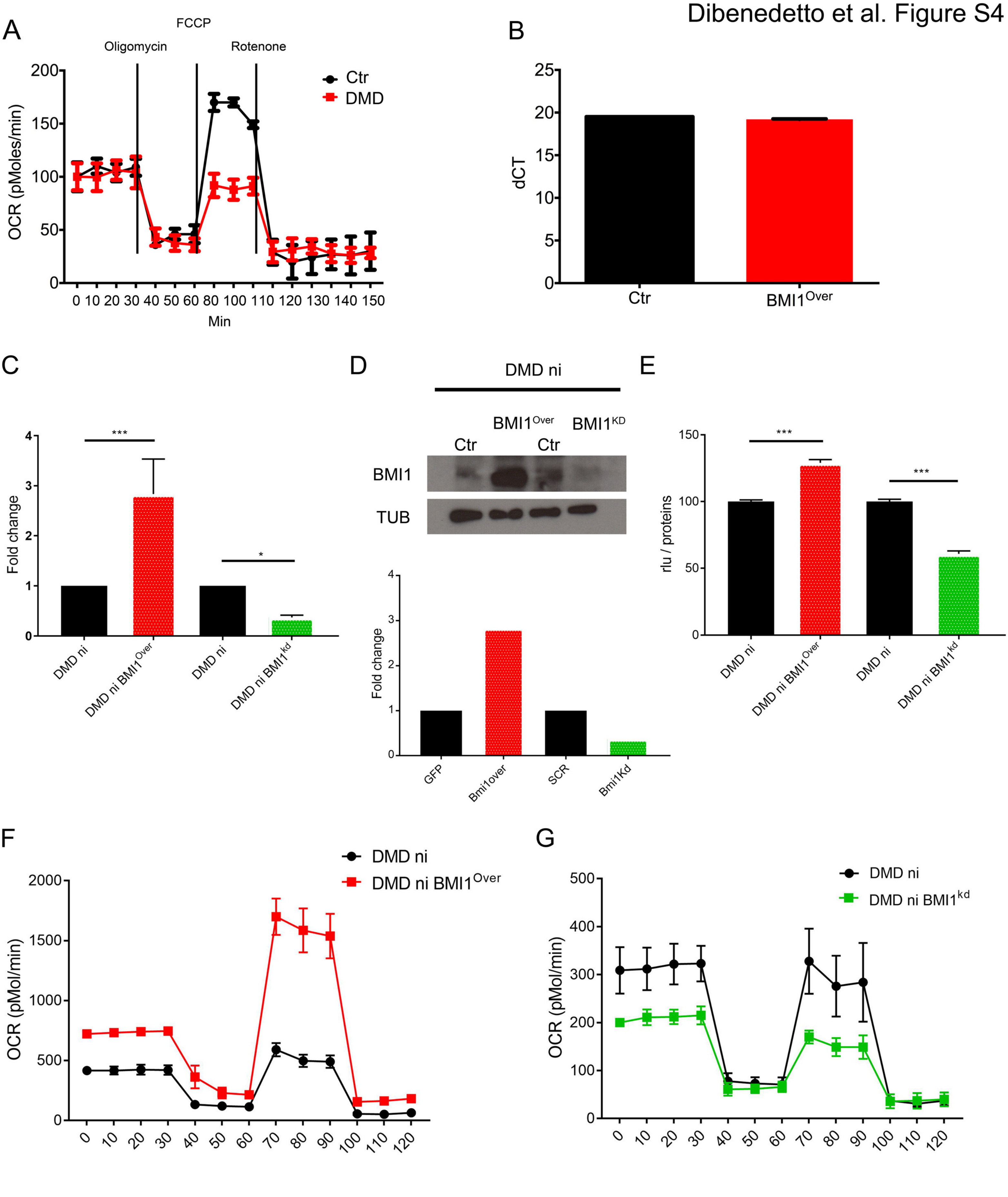

Dibenedetto et al. Figure S5

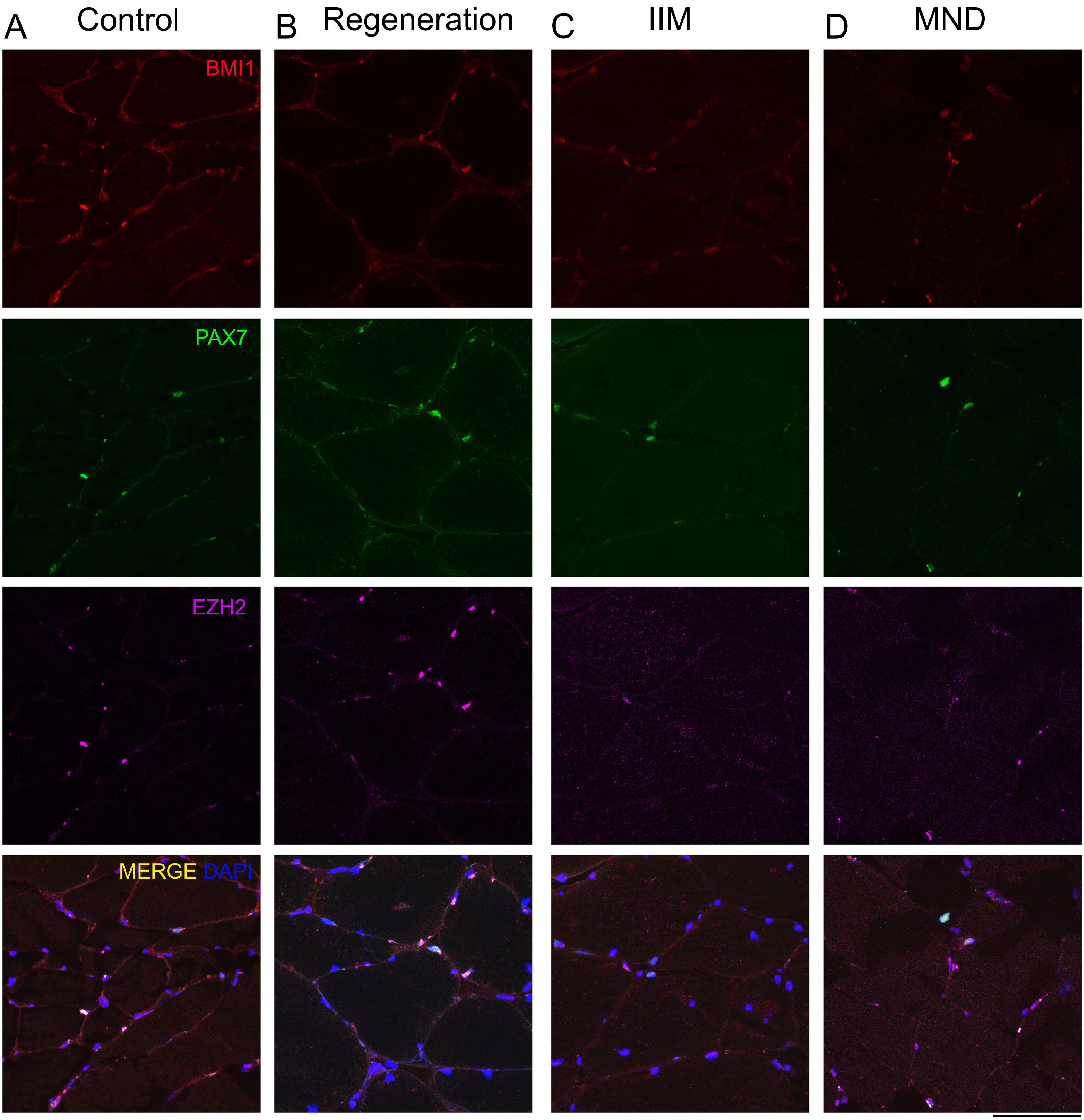

Dibenedetto et al. Figure S6

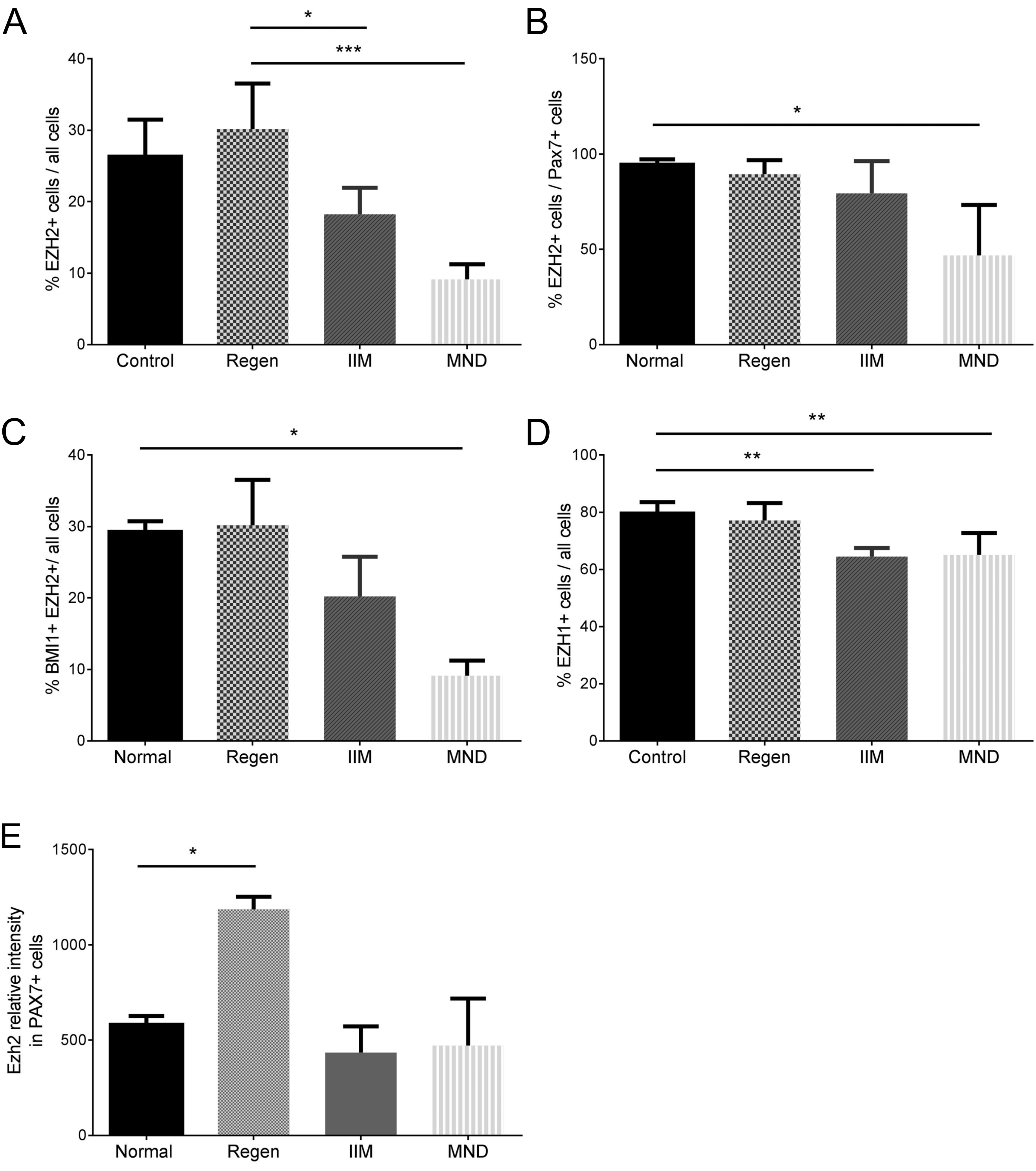

## Supplementary Figure Legends

**Fig S1.Depletion of EZH1+ cells in DMD patients.**

A) Triple immunostaining for BMI1, EZH1 and PAX7 on frozen muscle transverse sections of DMD patients (n=4 patients <5 year old; n=4 patients >5 year old) and aged matched controls (n=4 patients <5 year old; n=4 patients >5 year old). Representative images of the staining on >5 year old DMD and control muscles are shown. B) Quantification of EZH1+ cells over the total number of nuclei. C) Quantification of BMI1+;EZH1+ cells over the total number of nuclei. D) Quantification of EZH1+ cells over the total number of PAX7- cells and E) PAX7+ cells (mean ± SD; *, p <0.05; **, p<0.01). Quantification of the number of +ve cells was carried out on at least 5 high power fields (40x) for each case. Scale bar is 125μm

**Fig S2. Increased proliferation and differentiation in normal human myoblasts overexpressing BMI1.**

Control human myoblasts are infected with a *GFP* or *BMI1* encoding lentiviral particles and with a SCR or *BMI1*-shRNA lentiviral particles. (A) Representative images of EdU and MyHC staining on human myoblasts in proliferation medium, 48 and 96hrs after induction of differentiation. Quantification of the percentage of positive cells for EdU (B) and MyHC over total number of nuclei at 48hrs is shown in (C). Differentiation rate at 96hrs after induction of differentiation is expressed as percentage of fusion index (D) (mean ± SD of three independent experiments; *, p<0.05 **, p<0.01; ***,p<0.001). Scale bar is 250μm.

**Fig. S3. Predicted molecular networks in BMI1$^{Over}$ myoblasts.**

(A) Quantification of the band intensity of western blot shown in Fig.3D. (B-C) Predicted networks linking BMI1 and PRDX2, genes in grey are deregulated in our dataset. (D) Network of PDRX2 downstream target genes, genes in grey are deregulated in our dataset.

**Fig S4. BMI1 overexpression has no impact on mitochondrial biogenesis and it enhances mitochondrial respiration and ATP production in non immortalised DMD myoblasts.**

(A) Myoblasts control (black) and DMD myoblasts (red) were seeded in a Seahorse XF-24 analyzer and real-time OCR was determined during sequential treatments with oligomycin (ATP-synthase inhibitor), FCCP (mitochondria uncoupler) and rotenone (ETC inhibitors). A normalization of data generated by the XF24 was performed to observe the loss of the spare respiratory capacity (SRC). Data are representive of three independent experiments in which each data point represents the mean ± SD of 12 replicates for each condition.. (B) qRT-PCR showing the level of mtDNA copy number in DMD myoblasts upon BMI1 overexpression (red) as compared to the DMD control (black) (mean ± SD of three independent preparations;). Non immortalised DMD myoblasts are infected with a GFP (black bar) or BMI1 (red bar) encoding lentiviral particles and with a SCR (black bar) or BMI1-shRNA (green bar) lentiviral particles. Level of expression of BMI1 upon BMI1 overexpression or BMI1 knockdown was assessed by qRT-PCR (C) (mean ± SD of three independent preparations; *, p<0.05; ***,p<0.001) and by Western Blot (D), quantification of band intensity is shown (E). (F) ATP content measured with a luciferase assay and normalised against protein concentration in DMD ni GFP (black bar) or DMD ni BMI1$^{Over}$ (red bar) and DMD ni SCR (black bar) or DMD ni Bmi1kd (green bar) myoblasts (rlu/prot) (mean ± SD of three independent experiments;

***,p<0.001). (G,H) Real-time OCR determined with XF24 on DMD ni upon BMI1 overexpression (F) or BMI1kd (G) and compared to respective controls. Data are representive of three independent experiments in which each data point represents the mean ± SD of 6 replicates for each condition

**Fig S5. Characterisation of EZH2 expression in human chronic neuromuscular disorders.**

Representative images of a triple immunostaining for BMI1, PAX7 and EZH2 on frozen muscle transverse sections from morphologically normal muscle (n=6), normal muscle biopsies with evidence of regeneration (n=5), Idiopathic Inflammatory Myopathy (IIM) (n=5) and motorneurone disease (MND) (n=4). Scale bar is 125μm.

**FigS6. Reduction of EZH2 and EZH1 expression in human chronic neuromuscular disorders.**

(A) Quantification of EZH2+ cells over the total number of nuclei and (B) amongst PAX7+ satellite cells. (C) Percentage of BMI1, EZH2 double positive cells over the total number of nuclei. (D) Percentage of EZH1+ cells over the total number of nuclei. (mean ± SD; *, p<0.005; **, p<0.001; ***, p<0.0001). Quantification of the number of +ve cells was carried out on at least 5 high power fields (40x) for each case.

**Supplementary Experimental Procedures**

**Production of BMI1over and shRNA-BMI1 lentiviral vector**

pLox-CWBmi1 (#12240) and pLox-CWGFP (#12241) (used as control) (both from Addgene) lentiviral vectors were used to assess the impact of BMI1 overexpression in the myoblasts culture. A pGIPZ-lentiviral shRNA vector containing a hairpin sequence targeting *BMI1* (ThermoFisher, Clone Id: V3LHS_302124) was used to achieve *BMI1* knock-down. A pGIPZ lentiviral vector containing a scramble sequence was used as control. The lentiviral vectors were co-transfected with lentiviral packaging, using the calcium phosphate method into 293T cells. The medium was refreshed and viruses were harvested 48 hours after transfection, passed through 0.45-µm filters, concentrated by PEG precipitation, and stored at -80°C. The infectious titer was determined by FACS analysis of GFP positive 293T cells.

**Lentiviral mediated BMI1overexpression and knockdown**

Human myoblasts were seeded in multiple well plates and infected with BMI1 and GFP control (10 MOI) or shBMI1 and Scramble (30 MOI) lentivirus in proliferation medium. The medium was replaced on the second day and the transduction efficiency was analysed ninety-six hrs after infection by qPCR and WB.

**Immunohistochemistry**

All myoblast cultures and muscle sections were fixed with 4% PFA for 10 min.

For immuno-labelling primary antibodies were applied for one hour or overnight at room temperature or 4°C respectively for myoblasts or muscle section respectively; appropriate fluorescent secondary antibodies were used and the myogenic cultures or sections were mounted using Vectashield mounting medium with DAPI (Vector Laboratory).

**Peroxidase-based immunohistochemistry**

Sections were air dried and then blocked with normal horse serum for 10 min. (Vectastain kit). For the detection of fibers of human origin, sections were incubated for 1hour at RT with hSpectrin (1:100, VP-5283, Vector Laboratories) and hLamin A/C (1:100, NCL-LAM-A/C, Novacastra), followed by 30 min incubation in universal biotinylated serum (Vectastain). Sections were then treated for 20 min with Elite ABC complex (Vectastain) and for 10 min with liquid DAB (BioGenex). Finally sections were counterstain in Gill's haematoxylin, digitised to give 8-bit colour images at x40 magnification using a Leica SCN400F whole slide scanner, and the number and CSA of engrafted fibers was analysed with Definiens Developer.

Tissue areas were identified based on the lowest (darkest) pixel value at each pixel from the composite RGB image layers. The mean pixel value from these 'darkest' pixels was calculated and adjusted by -15 (this ensures only stained tissue is selected) was then used to separate tissue from background. The blue and brown components of the images were then identified using the HSD model (van Der Laak et al., 2000) which provides a direct measure of each colour. Brown regions were identified as spectrin, then spectrin regions were grown into intensely blue regions to close gaps in sarcolemma identification. Regions of positive fibres were then manually selected.

A combination of morphological measurements and object cutting processes were then used to optimise the identification and separation of positive fibres and their associated nuclei. Some nuclei associated with the sarcolemma have a high degree of overlap with the sarcolemma so cannot be identified as separate objects. These nuclei were inferred from the presence of a relatively dark spots associated with a

widening of the sarcolemma that satisfy inclusion criteria.  The area and width was then exported for each fibre.

**Antibodies**

The following primary antibodies were used: goat anti-BMI1 (Santa Cruz Biotechnology, sc-8906,1:100), Click-iT TM EdU Imaging Kits (Invitrogen, C-10340), rabbit anti-Laminin (Sigma-Aldrich, L-9393, 1:1000), rabbit anti-Myf5 (C-20) (Santa Cruz Biotechnology, sc-302, 1:100), mouse anti-pan myosin heavy chain (A4.1025), mouse anti myosin heavy chain type1(BA-F8), mouse anti myosin heavy chain type IIA (SC-71), mouse anti myosin heavy chain type IIX (6H1) (all from Developmental Studies Hybridoma Bank, 1:10), mouse anti-Pax7 (Developmental Studies Hybridoma Bank, 1:1), Rabbit anti-alpha sarcoglycan (EPR14773) (Abcam, ab189254,1:200), rabbit anti-γH2AX (phosphor S139)-DNA double-strand break marker (Abcam, ab11174,1:200), mouse anti-hSpectrin (Vector Laboratories, VP-5283, 1:100), mouse anti-hLamin A/C (Novacastra, NCL-LAM-A/C, 1:100), rabbit anti-Ezh1 (Millipore, ABE281, 1:100), rabbit anti-Ezh2 (Cell Signalling, D2C9,1:100). The following secondary antibodies were used: donkey anti-mouse IgG Alexa 488 (R37114, 1:1000), goat anti-mouse IgG1 Alexa 546 (A-21123, 1:1000), goat anti-mouse IgG1 Alexa 488 (A-21121, 1:1000), goat anti-mouse IgG2b  Alexa 555 (A-21147, 1:1000), goat anti-mouse IgM Alexa 488 (A-21042, 1:1000), donkey anti-rabbit IgG Alexa 488 (A-21206, 1:1000), donkey anti-rabbit IgG Alexa 647 (A-31573, 1:1000), donkey anti-rabbit IgG Alexa 546 (A-10040, 1:1000), donkey anti-goat Alexa 568 (A-11057, 1:1000) (all from ThermoFisher Scientific).

**Microscopy and quantification**

Fluorescent and bright field image capture was performed using a Leica epifluorescent microscope or Zeiss Meta 510 LSM or Zeiss LSM 710 confocal. In

myoblast cultures, all DAPI positive cells and antigen positive cells were counted and the percentage of Antigen$^+$ cells was calculated. Data from ≥3 cultures were pooled to give a population mean ± SD

For the quantification of the intensity staining all samples were stained simultaneously and the pictures were acquired with a Zeiss LSM 710 confocal at the same ratio of emissions. For the analysis in ImageJ software, the shape of each Pax7$^+$ cell and hLamin$^+$ cells was drawn around the nucleus and the intensity of antigen staining calculated for each cell as mean of Integrated Density (Mean Intensity*Area of the cell). The average Integrated Density was calculated among all cells per each field acquired.

**Measurement of cellular oxygen consumption rate and extracellular acidification rate**

An extracellular flux (XF) analyzer (Seahorse Bioscience, North Billerica, MA) was used to determine metabolic phenotype of cells. XF Analyzer simultaneously monitors oxygen consumption rate (OCR) and extracellular acidification rate (ECAR), which are indicators of mitochondrial respiration and aerobic glycolysis (lactate production), respectively, of cultured cells.

XF-24 cell culture microplates were coated with 3.4 mg/mL BD Cell-TakTM tissue adhesive solution (BD Bioscience 354240) according to manufacturer’s instruction. 43µl of the Cell-Tak solution was added to each well of a XF-24 cell culture plate and incubated for 20 min a room temperature.

Immortalised myoblasts were plated at densities of 50,000 cell per well in XF-24 cell culture plates and incubated overnight in 37°C incubators.

Non immortalised myoblasts (50,000 cell per well) were attached to Seahorse XF-24 plates pre-coated with Cell-TAK on the same day of the analysis. Microplates

containing the cell suspension were centrifuged at 700×g for 5 min and then incubated at 37°C for at least 1h to allow attachment.

Before measurements, the growth medium was replaced with 600 µl assay medium (Seahorse Bioscience), a low buffered DMEM containing no bicarbonate, and incubated for 45 minutes in a 37°C non-CO2 incubator. Basal oxygen consumption rate and extracellular acidification rate was determined using XF24 Extracellular Flux analyzer. Oxygen-consumption rate (OCR) was determined in response to sequential treatment with the ATPase inhibitor oligomycin, the uncoupling agent FCCP and the electron-transport-chain inhibitors rotenone. Oligomycin, carbonyl cyanide p-trifluoromethoxyphenylhydrazone (FCCP) and rotenone were obtained from Sigma, and stock solutions were prepared following the manufacturer's instruction.

**ATP measurement**

Human myoblasts were plated at a density of 60,000 cells/well in a 6 well plate. Cells were harvested 24hrs later by centrifugation at 1000rpm 180 g for 5 min to remove extracellular ATP. Harvested cells were suspended in 100µL cold water and sonicate for 5 min. Next, 50 µL of cell suspension was mixed with 50µL CellTiter-Glo Luminescent Cell Viability Assay solution (Promega, Madison, WI, USA), and incubated for 10 min. The luminescence intensity was measured using a Synergy HT Multi-Mode Microplate Reader. 2 µL of cell suspension was used to measured protein levels. All the readings were express as rlu (relative luminescence unit) /mg of proteins.

Conoidin A ( Cayman Chemical) was used as inhibitor of PRDX2. Non immortalised cells were treated with 2uM of Conoidin A for 24 hours at 37ºC. ATP level was measured as described above and compared to DMSO treated control cells.

For ATP measurement on mouse muscles, sections were scrapped off the slides and resuspended with 100 µl of cold PBS and then processed as described above.

**GSH/GSSG ratios**

Cells were plated in a 96 well plate at 5,000 cells per well density the day before the test. Reduced and oxidized Glutathione ratio was measured by using the GSH/GSSG-Glo assay kit (Promega) according to the manufacturer's protocol. Assay reagents were added directly to cells cultured in the multiwell plates. The GSH/GSSG-Glo™ Assay is a luminescence-based system for the detection and quantification of GSH/GSSG ratios in cultured cells. The luminescence intensity was measured using a Synergy HT Multi-Mode Microplate Reader and normalised against protein concentration.

**DNA damage measurement**

Cells were treated for 10 min at RT with 50µM $H_2O_2$. Medium was then replaced with fresh one and cells were incubated for 45 min in a 37ºC incubator to allow for DNA repair process to occur. Cells were then washed with PBS, fix for 10 min with 4% PFA and then immunostained with γH2AX antibody. Cell images were acquired using an InCell 1000 automated microscope (GE), and then analysed using InCell Developer Toolbox software (GE) to determine the mean area of foci and mean intensity of the staining. Data were averaged for the triplicate technical replicates and compared to the untreated wells.

**DNA, RNA extraction and qPCR analysis**

DNA extraction was performed using the DNeasy Blood and Tissue kit (Qiagen). RNA extraction was carried out using the RNeasy Micro purification kit (Qiagen) and DNase digestion was performed to remove genomic DNA. cDNA synthesis was performed with SuperScript III Reverse Transcriptase Kit (Invitrogen). qRT-PCR

analysis was performed with Taqman assays (AB Applied Biosystems) with FAM labeled probes in 96 well plates using the Applied Biosystems 7500 RT PCR machine according to the manufacturer's instructions (*BMI1*:Hs00180411_m1; *MT1*: Hs01938284_g1; *PRDX2*: Hs00853603_s1; *GPX3*: Hs01078668_m1; *MT-ND1*: Hs02596873_s1). The cDNA content was normalized against the expression of the housekeeping gene *GAPDH* (Hs02758991_g1) (technical duplicates for each culture and ≥3 independently derived cultures or biological samples were analysed).

**Western blot**

Myoblasts were lysed for one hour on ice with RIPA buffer (1% Igepal CA-630, 2 M TrisHCl pH 8.0, 0.5% Na-deoxycholate, 0.1% SDS, 2 mM EDTA, 150 mM NaCl including Mini protease inhibitor complete cocktail (Sigma Aldrich, 11836153001)) followed by max speed centrifugation (10 mins). The supernatants were collected and the protein concentration was measured using BCA Protein Assay Kit (Pierce). Twenty µg of protein per lane were separated on a NuPage 4-12% Bis-Tris Gel (Invitrogen) for 2hr at 100mA and electroblotted onto nitrocellulose PROTRAN (Amersham) 1 hr at 100V. After blocking 1hr at RT in TBST buffer (25 mMTrisHCl, 137 mMNaCl, 0.1% Tween 20, pH 7.5) containing 5% skimmed milk, immunodetection of proteins was performed with mouse anti-BMI1 (Clone F6)(Millipore, 06-637, 1:2000), rabbit anti Peroxiredoxin 2 (Abcam, ab59539, 1:2000), rabbit anti $SO_3$ Peroxiredoxin (Abcam, ab16830, 1:2000) ON at 4º C followed by HRP-conjugated anti-mouse (NA931) and anti-rabbit (NA934) IgG secondary antibody (1:5000; Amersham for 1hr) at room temperature. Enhanced chemoluminescence (ECL Plus; Amersham) was used for detection of the bands. Mouse anti-α-tubulin (Sigma, T5168, 1:5000) and Mouse anti vinculin (Sigma, V4505, 1:5000) were used as a control for gel loading.

**Proliferation and differentiation assays**

Cells were plated in growth medium on 13 cm diameter coverglass coated with Matrigel (90µg/ml) in 24 well plates at 20,000 cell density per well. The day after medium was replaced with differentiation medium and proliferation rate was assessed via EdU incorporation 24hrs after induction of differentiation. Cells were treated for 2hrs 30min with 10uM EdU solution (Click-iT EdU Imaging kit, TermoFisher, C-9393), then washed with PBS and fixed with 4% PFA for 10 min. Cells were permeabilized for 20 min with 0,3% Triton-PBS and then incubated for 30 min in the dark with the Click-iT reaction cocktail (Click-iT EdU Imaging kit, TermoFisher) to detect EdU incorporation.

To evaluate the differentiation rate, cells were plated and maintained as above. Cells were fixed with 4% PFA at 48 and 96 hrs after induction of differentiation and immunostained for MyHC. Coverglass were then mounted with Vectashield mounting medium plus Dapi and pictures acquired with Leica Epifluorescent microscope. Proliferation was assessed as percentage of EdU positive nuclei over the total number of nuclei. Differentiation was assessed as percentage of MyHC positive cells (either single cells at 48hrs or myotubes at 96hrs) over the total number of cells. Fusion index was expressed as percentage of the sum of nuclei in multinucleated fibers over the total number of nuclei.

**RNASeq analysis**

Three independently prepared normal and DMD myoblasts cell cultures infected with GFP and BMI1$^{Over}$ lentiviral particles and induced to differentiate for 2 days were analysed by RNA sequencing (RNASeq).

One microgram of total RNA was used for library preparation, according to the Illumina TruSeq mRNA sample preparation protocol (Illumina, San Diego). Libraries

were sequenced using the Illumina NextSeq platform and TruSeq protocol. Adaptor and poor quality sequences were removed using trim galore (v 0.3.7) software. Trimmed sequences were aligned to the human reference genome (GRCh37) using TopHat2 (v2.0.13)/bowtie2(v2.2.3). The read counts were calculated using the software HTSeq-counts. All the analyses described above were implemented using standard protocols.

Differential expression was calculated for i) differences between DMD vs DMD BMI1$^{Over}$ and ii) differences between Ctr vs Ctr BMI1$^{Over}$. Genes with zero counts were removed prior to the statistical analyses. Generalized linear models from DESeq2 (Love et al., 2014) were applied to test for differential expression. To account for the paired cell lines used in the experiment between Ctr and BMI1$^{Over}$ we included the cell line as a fixed effect in the design matrix of the linear model applied (i.e. formula(~cell + condition). Pathway analysis was performed using Ingenuity Pathway Analysis (Qiagen Redwood city, CA). Datasets are available at GEO, E-MTAB-5846.

**Treadmill exercise**

The dystrophic animals *mdx;*Pax7$^{Bmi1}$, *mdx* control littermates and C57BL/6-BL/10 mice (2-4 month) used here were those described in (Di Foggia et al., 2014). In brief, mice were injected with Tamoxifen to conditionally activate Bmi1 overexpression in satellite cells. Three days after the last injection, the treadmill exercise regime was started. Each mouse was put in a lane of the treadmill and forced to run at the speed of 12m/min for 30 min twice a week, for 6 consecutive weeks. Six days after the last treadmill session the mice were sacrificed. One forelimb was embedded in OCT and frozen in isopentane cooled at the liquid nitrogen temperature.
